# Involvement of the TNF-α/SATB2 axis in the induced apoptosis and inhibited autophagy of osteoblasts by the antipsychotic Risperidone

**DOI:** 10.1186/s10020-022-00466-9

**Published:** 2022-05-03

**Authors:** Shuyao Zhang, Wei He, Aiguo Li, Chengkuan Zhao, Yun Chen, Chengcheng Xu, Qiuzhen Zhang, Danling Zheng, Meini Chen, Haixiong Miao, Yihui Huang

**Affiliations:** 1 Department of Pharmacy, Guangzhou Red Cross Hospital, Jinan University, Guangzhou, 510220 China; 2 Department of Orthopaedics, Guangzhou Red Cross Hospital, Jinan University, Guangzhou, 510220 China; 3 Department of Pharmacology, Guangzhou Red Cross Hospital, Jinan University, Guangzhou, 510220 China; 4 Department of Pediatrics, Shantou University Medical College, Shantou, 515041 China; 5 Department of Pediatrics, Guangzhou Red Cross Hospital, Jinan University, No. 396, Tongfuzhong Road, Haizhu District, Guangzhou, 510220 Guangdong China

**Keywords:** Risperidone, Tumor necrosis factor-α, SATB2, Osteogenic differentiation, Differentiation, Autophagy, Apoptosis

## Abstract

**Background:**

Risperidone, an atypical antipsychotic, impedes serotonin and dopamine receptor systems. Meanwhile, tumor necrosis factor-α (TNF-α) is known to participate in regulating osteoblast functions. Consequently, the current study aimed to investigate whether the influences of Risperidone on osteoblast functions are associated with TNF-α and special AT-rich sequence-binding protein (SATB2).

**Methods:**

Firstly, we searched the DGIdb, MEM and GeneCards databases to identify the critical factors involved in the effects of Risperidone on osteoblasts, as well as their interactions. Afterwards, osteoblast cell line MC3T3-E1 was transduced with lentivirus carrying si-TNF-α, si-SATB2 or both and subsequently treated with Risperidone. Various abilities including differentiation, autophagy and apoptosis of osteoblasts were examined after different treatments. Finally, animal experiments were performed with Risperidone alone or together with lentivirus to verify the function of Risperidone in vivo and the mechanism.

**Results:**

It was found that Risperidone might promote TNF-α expression, thereby inhibiting the expression of SATB2 to affect the autophagy and apoptosis in osteoblasts. Furthermore, as shown by our experimental findings, Risperidone treatment inhibited the differentiation and autophagy, and promoted the apoptosis of osteoblasts, as evidenced by elevated levels of OPG, p62, cleaved PARP1, cleaved caspase-3, cleaved caspase-8, and cleaved caspase-9, and reduced levels of LC3 II/I, Beclin1, collagen I, and RANKL. In addition, Risperidone was also found to elevate the expression of TNF-α to down-regulate SATB2, thereby inhibiting the differentiation and autophagy and enhancing the apoptosis of osteoblasts in vitro and in vivo.

**Conclusions:**

Collectively, our findings indicated that Risperidone affects the differentiation of osteoblasts by inhibiting autophagy and enhancing apoptosis via TNF-α-mediated down-regulation of SATB2.

**Supplementary Information:**

The online version contains supplementary material available at 10.1186/s10020-022-00466-9.

## Background

Despite comprising a mere 4—6% of the total resident cells in the bone, osteoblasts exert major functions in acquiring and maintaining appropriate bone mass through tight-regulation with other bone cells, including osteoclasts and osteocytes (Capulli et al. 2014). Inherently, osteoblasts originating from multipotential mesenchymal stem cells possess the ability to differentiate into adipocytes, myoblasts, chondrocytes and fibroblasts, which play crucial and diverse roles in bone formation (Tamma and Zallone 2012). Moreover, failure to recruit osteoblasts to the resorption site can elicit bone diseases featured by enhanced fracture risk and bone loss, such as osteoporosis (Kristensen et al. 2014). In addition, the process of osteoblastic differentiation is further known to play important roles in bone formation, regeneration and repair (Lo et al. 2012). On a separate note, autophagy, a critical catabolic process of eukaryotic cells, recycles and deteriorates impaired organelles and macromolecules, while autophagy in osteoblasts is associated with bone homeostasis and mineralization (Nollet et al. 2014). Moreover, recent studies have also illustrated that autophagy exerts a prominent effect on the differentiation of osteoblasts on titanium-based surfaces (Kaluderovic et al. 2014), which highlights the possibility of potential interaction between osteoblasts and autophagy.

Risperidone, a widely-known second-generation antipsychotic, is used for treating irritability in children and adolescents diagnosed with autistic disorder (Kent et al. 2013). Furthermore, Risperidone is adopted for the treatment of attention deficit disorders, and in adolescents and adults affected by bipolar disorders and schizophrenia. Meanwhile, Risperidone, together with amisulpride and other older antipsychotics, is associated with a potential mechanism of bone loss due to blockade of dopamine receptors to induce hyperprolactinemia and consequent hypothalamic hypogonadism (Motyl et al. 2012; Jones 2014). It is also noteworthy that Risperidone-treated mature dendritic cells are known to exhibit elevated expression of tumor necrosis factor-α (TNF-α) and enhanced apoptosis of neutrophils (Chen et al. 2012). TNF-α is a multipotent cytokine that is capable of inducing various stimuli in multiple pathological and physiological environments, while also being actively implicated in cellular processes of dendritic cells, B cells, and T cells (Postal and Appenzeller 2011). More importantly, different concentrations of TNF-α are known to confer important roles in the osteogenic differentiation of bone marrow mesenchymal stem cells (BMSCs) (Wang et al. 2015). Recent studies have further indicated TNF-α as an inhibitor of osteoblast differentiation due to its ability to inhibit the expression of special AT-rich sequence-binding protein (SATB2) (Zuo et al. 2018). The SATB2 protein was also indicated to serve as a strong transcription factor associated with bone regeneration and osteoblastogenesis (Zhang et al. 2011). Additionally, SATB2 is further recognized as an important biomarker for osteogenic differentiation, which plays a pivotal role in osteoblast maturation and differentiation (He et al. 2017). Based on the above-mentioned published findings, we hypothesized that Risperidone may be implicated in the cellular progression and functions of osteoblasts through regulation of TNF-α and SATB2. Therefore, the current study set out to elucidate the mechanism of Risperidone in osteoblasts related to TNF-α and SATB2, and to provide more data for strengthening the understanding of related osteopathy.

## Methods

### Ethics statement

Animal experimentation protocols were approved by the Institutional Animal Care and Use Committee of Guangzhou Red Cross Hospital, Jinan University and conformed to the Guide for the Care and Use of Laboratory Animals published by the US National Institutes of Health. Extensive efforts were made to minimize the number and suffering of the included animals.

### Bioinformatics analysis

First, the Drug Gene Interaction Database was retrieved to screen candidate genes that might interact with Risperidone. In addition, the GeneCards database (Score > 5) was employed to predict the genes related to osteoblast autophagy with “osteoblasts” and “autophagy” serving as the key words. The intersection of the aforementioned genes was then obtained using the jvenn tool. Subsequently, the downstream genes of the critical factor were predicted with the help of the MEM database (https://biit.cs.ut.ee/mem/index.cgi), where the robust rank aggregation (RRA) algorithm was utilized to rank the candidate genes, with *p* < 2.24E−06 serving as the threshold (Kolde et al. 2012). The jvenn tool was adopted again to predict the intersection of the candidate genes from the MEM database and the genes related to osteoblast autophagy from the GeneCards database. Finally, the GeneMANIA tool was applied to analyze the co-expression relationship among the candidate genes, with the critical target genes further screened based on co-expression.

### Experimental animals

Six-week-old female C57BL/6J mice were procured from Beijing Charles River Experimental Animal Technology Co., Ltd. (Beijing, China) and fed with drinking water and diet, which met the (AIN)93G criteria as recommended by the American Society for Nutrition. At the age of 8 weeks old, the mice were randomly divided into 5 groups (6 mice in each group). All the mice were housed in a standard controlled animal care cage (6 mice per cage) and consecutively fed with a wet-mash containing Risperidone at different doses (0, 0.3, 0.45, 0.6 and 0.75 mg/kg) for a duration of 4 weeks. The dose of Risperidone was determined according to preliminary pharmacokinetics where the plasma concentration of Risperidone was assessed by administration of various doses of Risperidone by oral tube feeding, and a dosage of 0.75 mg/kg was found to reach the maximum of Risperidone plasma and its metabolites, similar to that in patients administrated with Risperidone. Furthermore, in female C57BL/6J mice, Risperidone at this dosage led to trabecular bone loss without affecting the adipose tissue weight.

### Micro-computed tomography (CT)

After 4 weeks, the mice were euthanized by cervical dislocation and the femurs were isolated from soft tissues and stored in 70% ethanol at 4℃. Next, scancoμct-35 (Scanco, Brutissellen, Switzerland) was adopted to analyze the specimens by means of cone-beam microfocus X-ray CT. The images were captured at 55 kvp, with an integration time of 500 ms and an isometric voxel of 6 mm. The femurs in the marrow and soft tissues were isolated with a constrained gaussian filter (support = 1; voxel window, 3 × 3 × 3; σ = 0.8). The density thresholds of 250 and 420 were applied to the trabecula and cortex chamber of the femur. An area for trabecula analysis was selected within the endosseous boundary at the distant metaphysis of the femur, which included the secondary sponge localized at 1 mm of growth late with 1 mm near-end extension. The quantity, volume and average cortex morphology were measured in 233 successive cross-Sections (1.4 mm) at the center of backbone midpoint between the near-end and far-end growth plates. Bone trabecular structures were then observed, including determination of bone volume fraction (BVF, no unit), trabecular thickness 2 d/3 d (TbTh, μm), spoon 2 d/3 d (trabecular distance, μm), trabecular number 2 d/3 d (TbN, 1/mm), trabecular bone mode factor (TbPf, no unit), bone surface area 2 d/3 d (BSA, mm^2^), and connection density (Conn., 1/mm^3^). In addition, after the phantom object calibration was corrected using hydroxyapatite, trabecular bone mineralization was quantified as bone mineral content (BMC, mg) and bone mineral density (BMD, mg/cm^3^).

### Hematoxylin–eosin (HE) staining

Following feeding with a wet-mash containing Risperidone (Hebei Cangzhou Enke Pharmaceutical Technology Co., Ltd., Hebei, China) at different doses, the mice were euthanized by cervical dislocation to collect left femur tissues and the excess tissues were removed. The obtained tissues were then fixed in 10% neutral buffered formalin at room temperature for 48 h, decalcified with 10% ethylene diamine tetraacetic acid (EDTA) at room temperature for 2 weeks, dehydrated with 70% ethanol and paraffin-embedded. Subsequently, the paraffin-embedded tissues were sectioned at a thickness of 5 μm using a slicer and stained with HE Staining kits (C0105, Beyotime, Shanghai, China) as per the manufacturer’s instructions. Three sections from 5 mice in each group were selected and observed under an inverted microscope (IX73, Olympus Optical Co., Ltd., Tokyo, Japan), with the representative images regarded as the experimental results.

### Western blot analysis

Following feeding with a wet-mash containing Risperidone at different doses, the mice were euthanized by cervical dislocation to collect femur tissues. The obtained tissues were ground into powder and then mixed with cell lysis buffer, followed by total protein extraction. Next, the protein concentration was determined using bicinchoninic acid kits (BCA) (Pierce, Rockford, IL, USA). Subsequently, the proteins was subjected to separation using 4% concentration gel and 10% spacer gel and electroblotted onto a membrane. Following sealing with 0.5% bovine serum albumin (BSA), the membrane was probed with the following diluted primary antibodies: light chain 3 (LC3)A/B (dilution ratio of 1:2000, ab128025, Abcam Inc., Cambridge, UK), Beclin1 (dilution ratio of 1:2000, ab207612, Abcam), p62 (dilution ratio of 1:1000, ab109012, Abcam), cleaved-poly(ADP-ribose) polymerase 1 (PARP1) (dilution ratio of 1:1000, ab32064, Abcam), cleaved caspase-3 (dilution ratio of 1:500, ab214430, Abcam), cleaved caspase-8 (dilution ratio of 1:1000, 8592S, Cell Signaling Technologies [CST], Beverly, MA,USA), cleaved caspase-9 (dilution ratio of 1:1000, 9507S, CST), osteoprotegerin (OPG; dilution ratio of 1:1000, ab183910, Abcam), Collagen 1 (dilution ratio of 1:1000, ab260043, Abcam), receptor activator of nuclear factor (NF)-kB-ligand (RANKL) (dilution ratio of 1:2000, PA5-110268, Invitrogen Inc., Carlsbad, CA, USA), TNF-α (ab183218, Abcam), and SATB2 (dilution ratio of 1:2000, ab92446, Abcam). Afterwards, the membrane was re-probed with horseradish peroxidase (HRP)-labeled goat secondary antibody goat anti-rabbit IgG (ab6721, Abcam) or goat anti-mouse IgG (ab6789, Abcam) for 2 h at room temperature. Later, the membrane was developed with 3,3'-diaminobenzidine tetrahydrochloride (DAB) solution and photographed using a Gel Imager (GelDocXR, Bio-Rad, Hercules, CA, USA). The ratio of the gray value of the target band to that of glyceraldehyde-3-phosphate dehydrogenase (GAPDH) was representative of the relative protein expression.

### Double-labeled immunofluorescence assay

Paraffin sections of femur tissues were assigned into three groups. One portion of the paraffin sections were dewaxed, treated with gradient alcohol, immersed in 0.01 mol/L citric acid buffer at 95℃ for 30 min, and cooled at room temperature for 10 min, followed by antigen retrieval. These sections were then treated with PBS containing 0.3% Triton X-100 (Sigma-Aldrich Chemical Company, St Louis, MO, USA) at room temperature for 5 min and blocked with 1% goat serum albumin for 2 h. Next, the sections were immunostained with diluted primary antibodies against RUNX2 (dilution ratio of 1:100, 232902, Novus), LC3A (dilution ratio of 1:100, PA5-22990, Thermo Fisher Scientific Inc., Waltham, MA, USA), LC3B (dilution ratio of 1:250, ab63817, Abcam), Beclin1 (dilution ratio of 1:200, ab217179, Abcam) and p62 (1:100, ab155686, Abcam) overnight at 4℃. After PBS rinsing, the sections were re-immunostained with secondary antibody Alexa Fluor® 647-labled goat anti-rat (dilution ratio of 1:2000, ab150167, abcam) and Alexa Fluor® 488-labled goat anti-rabbit (dilution ratio of 1:2000, ab150077, Abcam) for 1 h. Co-localization of LC3, Beclin1, and p62 with RUNX2 was detected using double-labeled immunofluorescence. The nuclei were stained with 4',6-diamidino-2-phenylindole (DAPI) and observed under a Leica fluorescence microscope. The primary antibody was substituted with the non-immune isotype antibody as the negative control (NC). The fluorescence intensity was measured with the help of the Image-J 1.48v software (http://imagej.nih.gov/ij/) and expressed as integral density (IntDen) for single channel and co-labeling (red + green = yellow). IntDen is the average fluorescence normalized to the number of cells in the selected region. For each experimental condition, at least three slides were prepared and fluorescence in 100–200 cells in a total of six to ten randomly selected microscope fields was quantified.

### Cell culture and treatment

MC3T3-E1 osteoblasts [American Type Culture Collection (ATCC), Manassas, VA, USA] were incubated in α-minimal essential medium (MEM) supplemented with 10% fetal bovine serum (FBS) and 1% streptomycin at 37℃ with 5% CO_2_ in air. Upon reaching 80–90% confluence, the cells were passaged continuously. Subsequently, the cells were washed twice with D-Hank solution and detached with 1 mL of 0.25% trypsin–EDTA (Gibco, Carlsbad, California, USA). Next, the medium containing FBS was added to halt the detachment and cells were cultured in several T25 flasks. Afterwards, 10 mM stock solution was prepared in dimethyl sulfoxide (DMSO) solution.

In order to evaluate the effects of Risperidone on the differentiation, autophagy and apoptosis of osteoblasts, the cells were divided into a control group and a Risperidone group. For the cells in the Risperidone group, an osteogenic differentiation medium (Cyagen, Guangzhou, China) was adopted to induce osteogenic differentiation according to the manufacturer’s instructions. In short, MC3T3-E1 cells and BMSCs were seeded in 24-well plates and cultured in the above-mentioned osteogenic differentiation medium. Published literature has reported that Risperidone affects osteoblasts in a dose-dependent manner. Thereafter, 150 μmol/L of Risperidone was added to the cells in the Risperidone group and allowed to react for 48 h. The osteogenic differentiation medium was changed every day. After 2 days of culture, follow-up experiments were carried out. The cells in the control group were not treated with Risperidone. Three replicates were set for each group.

### Cell transduction

Upon reaching 80–90% confluence, the MC3T3-E1 cells were transduced with lentivirus (Genomeditech, Shanghai, China) (http://www.genomeditech.com) carrying small interfering RNA-NC (si-NC), si-TNF-α, si-SATB2, or combined with the treatment with Risperidone (Risperidone + si-NC, Risperidone + si-TNF-α, Risperidone + si-SATB2, and Risperidone + si-TNF-α + si-SATB2 groups). All siRNA sequences are listed in Additional file 2: Table S1. Following transduction, the cells were cultured at 37 °C with 5% CO_2_ and saturated humidity. After 6 h, the medium containing the transduction solution was discarded, whereupon the cells were cultured in a CO_2_ incubator (Thermo Fisher Scientific) with 10% FBS and 1% penicillin–streptomycin solution at 37 °C for 72 h. To avoid nonspecific targeting, each target gene was designed to contain two siRNA sequences. The transfection reagents were purchased from Shanghai GenePharma Co., Ltd. (Shanghai, China).

### Culture and staining of osteoclasts

BMSCs were isolated from femur and tibia of 8-week-old female C56BL/6J using centrifugation. The obtained cells were seeded in 96-well plates at a density of 4.5 × 10^5^ cells/well, and then cultured in 200 μL osteoclast medium with αMEM, 10% FBS, 1% PS and 30 ng/mL macrophage colony-stimulating factor (M-CSF) and 100 ng/mL of RANKL (PeproTech (Rocky Hill, NJ, USA). Meanwhile, osteoclasts in the Risperidone group were treated with 150 μmol/L Risperidone and the medium and treatment methods were changed on day 3 and day 6. Between day 5 and day 7, the cells were identified as osteoclasts, fixed with 2.5% glutaraldehyde and stained with tartaric acid phosphatase (TRAP; #387A, Sigma-Aldrich). The TRAP-positive multinucleated (four or more nuclei) cells were regarded as osteoclasts and counted under a light microscope. The total number of osteoclasts per well was calculated, and at least 6–8 wells per treatment were calculated from three independent experiments. All results were expressed as mean ± standard deviation.

### Alkaline phosphatase (ALP) staining

MC3T3-E1 cells (1 × 10^5^ cells/mL) were rinsed twice with phosphate buffer saline (PBS) containing 4% neutral formalin at room temperature (5 min per rinse), and then washed twice with 0.5% Tris-buffered saline with Tween-20 (TBST). Afterwards, the cells were stained with ALP in conditions void of light for 30 min at 37 °C (D001-1, NanJing JianCheng Bioengineering Institute, Nanjing, China), and then observed under a microscope.

### Alizarine red S staining

MC3T3-E1 cells were rinsed thrice with PBS, fixed with 4% paraformaldehyde, and then stained with 0.1% alizarin red S in distilled water (pH 4.2) at 20 °C for 1 h. Following three washes with distilled water, the stained cells were observed under an inverted phase contrast microscope. In order to quantify the results of alizarin red S, the precipitate was dissolved in a solution of 20% methanol and 10% acetic acid, and measured at 450 nm by means of spectrophotometry.

### Flow cytometry

After 48 h of transduction, the cells were detached with 0.25% trypsin without EDTA (YB15050057, Shanghai Yubo Biotechnology Co., Ltd., Shanghai, China), and then collected in a flow tube and centrifuged, with the supernatant discarded. Following three rinses with cold PBS, the cells were centrifuged and the supernatant was removed. According to the instructions of the Annexin-V-fluorescein isothiocyanate (FITC) cell apoptosis detection kit (K201-100, BioVision, Milpitas, CA, USA), Annexin-V-FITC, propidium iodide (PI) and N-2-hydroxyethyl-piperazine-N'-2-ethanesulfonic acid (HEPES) buffer solution were mixed with the Annexin-V-FITC/PI staining solution at a ratio of 1:2:50. Next, the cells were resuspended at a final density of 1 × 10^6^ cells per 100 μL of dye solution and mixed by shaking. After incubation at room temperature for 15 min, 1 mL of HEPES buffer solution (PB180325, Wuhan Procell Life Science & Technology Co., Ltd., Wuhan, China) was added to the cells and mixed. Afterwards, both FITC and PI were excited at 488 nm, and detected at 525 and 620 nm, respectively.

After treatment with Risperidone or lentivirus for 1 day, cell apoptosis was analyzed with the help of Annexin-V-FITC cell apoptosis detection kits (BD Pharmingen, Franklin lakes, NJ, USA). After rinsing with cold PBS, the mixture of 100 μL of PI (2 μg/mL) and Annexin-V (2 μL) was used to stain the cells in conditions void of light for 15 min at room temperature. After adding 1 × binding buffer (300 μL) into each tube, cell apoptosis was quantified using flow cytometry.

### Reverse transcription quantitative polymerase chain reaction (RT-qPCR)

Total RNA content was extracted from the tissues using RNeasy Mini kits (Qiagen, Valencia, CA, USA), and then reverse-transcribed into complementary DNA (cDNA) with reverse transcription kits (RR047A, Takara, Tokyo, Japan). Next, RT-qPCR was conducted using the SYBR Premix EX Taq kit (RR420A, Takara, Tokyo, Japan) on an ABI 7500 instrument (Applied Biosystems, Foster City, CA, USA). Three duplicate wells were set for each sample. All primers were synthesized by Shanghai Sangon Biotechnology Co., Ltd. (Shanghai, China), with the sequences shown in Additional file 3: Table S2. With GAPDH serving as the internal reference, the relative expression of the target genes was calculated using the 2^−ΔΔCt^ method.

### Immunofluorescence

MC3T3-E1 cells were rinsed with 0.01 mol/L of PBS, fixed in 4% paraformaldehyde for 15 min and then blocked with 3% BSA for 2 h. Following PBS rinsing, the cells were immunostained with primary antibody against LC3-II (dilution ratio of 1: 1000, ab48394, Abcam) at 4℃ overnight, and then with green fluorescence-labeled secondary antibody at room temperature for 2 h. Afterwards, the cells were stained with DAPI (1 μg/mL) for 5 min and mounted before observation under a fluorescence microscope (Olympus). DAPI was employed as a nuclear specific marker.

### In vivo transfection experiment

A total of 21 six-week-old healthy female C57BL/6 mice were selected and injected with lentivirus expressing siRNA or combined with Risperidone (medium + si-NC, medium + si-TNF-α, medium + si-SATB2, Risperidone + si-NC, Risperidone + si-TNF-α, Risperidone + si-SATB2, and Risperidone + si-TNF-α + si-SATB2), with 3 mice per treatment protocol. After acclimation for one week in the facility, the mice in each group were injected with the corresponding titer of lentivirus via tail vein at a volume of 100 μL per mouse. If the mice died after the injection, healthy female mice of the same age were randomly supplemented.

Afterwards, the mice were fixed with tail vein injection fixator, and the tail of mice was wiped with 75% alcohol to make the tail vein fully dilated. The lentivirus diluted with 100 μL of PBS was extracted using an insulin syringe and injected into the mice via tail vein, at a slow speed and a uniform manner. One week after the injection, the femur tissues were extracted for further experimentation.

### Serum collection

Blood samples were collected by heart puncture following mouse euthanasia induced by inhalation of isoflurane. Afterwards, the samples were allowed to stand at room temperature to coagulate, and then centrifuged at 1500 × *g* for 15 min, with the serum collected and stored at -80℃ until determination.

### Terminal deoxynucleotidyl transferase-mediated dUTP-biotin nick end labeling (TUNEL) assay

MC3T3-E1 cells were incubated in a 6-well plate at a density of 5 × 10^3^ cells/well for 24 h, and then incubated with antimicrobial-free (ABF) at different concentrations for 12, 24 and 36 h. Afterwards, the MC3T3-E1 cells were fixed in 4% paraformaldehyde, infiltrated by 0.1% Triton X-100, incubated with TUNEL mixture, stained with DAPI in dark conditions and observed under a fluorescence microscope (DMI8, Leica, Wetzlar, Germany). The apoptosis of cells in mouse knee femur tissues was detected using TACS XL Apoptosis Detection kits (Trevigen, Gaithersburg, MD, USA). Osteoblasts with positive nuclei were counted and expressed as a percentage of the total number of osteoblasts per bone. The positive control included slides incubated with nuclease. Five trabecular regions were examined in each mouse, and the total number of osteoblasts in each bone was between 20 and 100.

### Immunohistochemistry

The fixed knee joint tissues of mice were sectioned into 5 μm slices, dewaxed, hydrated with gradient ethanol, immersed in 3% H_2_O_2_ for 10 min, and then subjected to antigen retrieval. After being sealed with 100 μL 5% BSA at 37 °C for 30 min, the slices were incubated with diluted primary antibody against 100 μL LC3 II (dilution ratio of 1: 1000, ab48394, Abcam Inc., Cambridge, MA, USA) at 4℃ overnight, and then incubated with the biotin-labeled secondary antibody goat anti-rabbit HY90046 (Shanghai Hengyuan Biological Technology Co., Ltd., Shanghai, China) at 37 °C for 30 min. Subsequently, the slices were incubated with a streptavidin-peroxidase solution (Beijing Zhongshan Biotechnology Co., Ltd., Beijing, China) at 37 °C for 30 min and visualized by DAB. The conventional steps were previously described (Kelkar et al. 2017).

### Enzyme-linked immunosorbent assay (ELISA)

After euthanasia, an appropriate amount of femur tissues of knee joint were collected and rinsed with precooled PBS (0.02 mol/L, pH 7.0–7.2) to remove blood. After being weighed, the tissues were cut and transferred to a glass homogenizer. PBS was added to the tissues according to the recommended mass volume ratio of 1:5 and ground on ice. The homogenate was broken by means of ultrasonic and cooled in an ice bath. The prepared homogenate was then centrifuged at 5000 × *g* for 5 min, and the supernatant was extracted for further detection. TNF-α levels in the supernatant were determined in accordance to the instructions of the ELISA kit (A106111-48 T, Shanghai Fusheng Biotechnology Co., Ltd., Shanghai, China). Carbonate-coated buffer solution (pH 9.6) was utilized to dilute the known antigen to 1–10 μg/mL, which was added to a 96-well plate (0.1 mL diluent each well) for incubation at 4℃ overnight. Each well was incubated with 0.1 mL supernatant of diluted specimens at 37℃ for 1 h. Blank, negative and positive wells were set as controls. Subsequently, each well was incubated with newly diluted enzyme-labeled antibody (0.1 mL) at 37℃ for 35—40 min, and then washed with ddH_2_O. Afterwards, each well was visualized with 0.1 mL freshly prepared tetramethylbenzidine (TMB) substrate (EL0001, InnoReagents Biotechnology Co., Ltd., Huzhou, Zhejiang, China) at 37 °C for 10–30 min and added with 2 M sulfuric acid (0.05 mL). A microplate reader (BS-1101, Detie Experimental Equipment Co., Ltd., Nanjing, Jiangsu, China) was utilized to detect the OD values in each well under a wavelength of 450 nm after the blank control well was zeroed. In addition, according to the above-mentioned method, the serum was collected, in which the levels of TNF-α, IL-6 and IL-10 were all measured. Each sample was determined twice to obtain the mean value.

### Statistical analysis

Statistical analyses were processed using the SPSS 21.0 statistical software (IBM Corp., Armonk, NY, USA). Measurement data were described as mean ± standard deviation. One-way analysis of variance (ANOVA) with Tukey's test was adopted to compare multi-group data of animal experiments, cell transduction experiments and in vivo transfection experiments. Unpaired *t-*test was applied to analyze data obeying normal distribution and homogeneity of variance between the control group and the Risperidone group in cell culture experiments. A value of *p* < *0.05* was considered to be indicative of statistical significance.

## Results

### Bioinformatics analysis predicts the relevant factors of Risperidone influencing osteoblasts

**Fig. 1:**
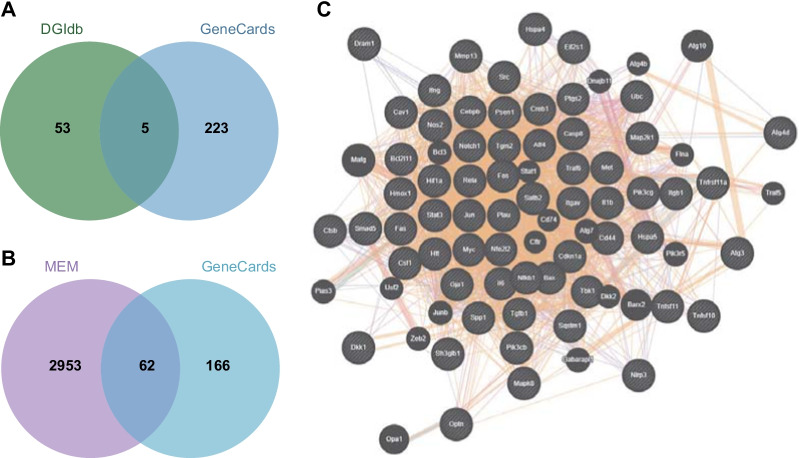
Bioinformatics analysis suggests a TNF-α/SATB2 axis in osteoblast autophagy. **A** Venn diagram of Risperidone-interacted genes obtained from the DGIdb database and osteoblast autophagy-associated genes from the GeneCards database. **B** Venn diagram of genes related to TNF-α from the MEM database and osteoblast autophagy-associated genes. **C** co-expression network of candidate genes in panel B

We first explored the DGIdb database to explore the critical factors involved in Risperidone regulating osteoblasts, which reared a total 58 potential genes interacting with Risperidone. We then screened autophagy-related genes in osteoblasts with GeneCards and 228 genes were obtained. By intersecting the genes from the two databases, five genes were found to be present in both the gene sets, namely, TNF-α, AKT1, TNFRSF11A, LEP and HTT (Fig. 1A), of which TNF-α exhibited the closest correlation to autophagy of osteoblasts with a score of 16.01. In addition, we further predicted the downstream genes of TNF-α at MEM database, wherein 3015 genes associated with TNF-α were listed and 62 of which were found to be involved in osteoblast autophagy (Fig. 1B). Subsequently, we adopted the GeneMANIA tool to establish a co-expression network of the 62 genes potentially involved in TNF-α-mediated effects of Risperidone on osteoblast autophagy (Fig. 1C). According to the co-expression network, SATB2 ranked the first with a point of 0.86 (Additional file 4: Table S3). These findings suggested that TNF-α may influence the autophagy of osteoblasts by modulating SATB2. Intriguingly, TNF-α has been previously reported to inhibit the differentiation of mesenchymal cell line (C2C12) to osteoblasts, and further significantly reduce the BMP-2 induced SATB2 expression (Zuo et al. 2018). Collectively, these findings suggested that Risperidone might influence autophagy of osteoblasts by regulating TNF-α and SATB2.

### Risperidone inhibits cell autophagy and promotes apoptosis in mouse femur of mice

**Fig. 2:**
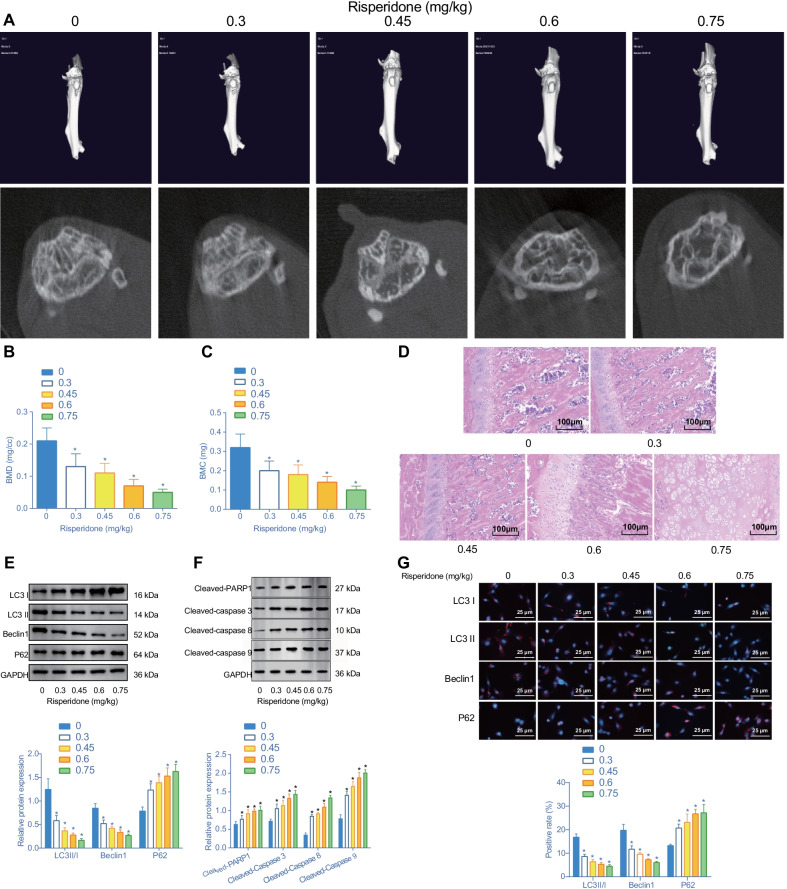
Risperidone causes an inhibition of autophagy and a promotion of apoptosis in femur tissues of mice. Mice were treated with 0, 0.3, 0.45, 0.6, 0.75 mg/kg of Risperidone. **A** representative micro-CT 3D and plan scan images of cross-sectional area of femur tissues. **B** statistical analysis of BMD in femur tissues of mice assessed by micro-CT. **C** statistical analysis of BMC in femur tissues of mice. **D** representative images of HE staining in femur tissues of mice. **E** Western blot analysis of autophagy-related proteins (LC3 II/I, Beclin1, and p62) expression in femur tissues of mice. The band intensity was assessed. **F** Western blot analysis of apoptosis-related proteins (cleaved PARP1, cleaved caspase-3, cleaved caspase-8 and cleaved caspase-9). Different numbers represent different concentrations of Risperidone (mg/kg). **G** co-localization of LC3 II (red), LC3 I (red), Beclin1 (red), and p62 (red) with the osteoblast marker RUNX2 (green) by double-labeled immunofluorescence, wherein the nucleus is labeled by DAPI (blue). *p < 0.05, compared with the treatment of 0 mg/kg of Risperidone. The experiments are repeated 3 times

To further elucidate the effect of Risperidone on femur tissues of mice, the mice were fed a diet containing Risperidone at different doses (0, 0.3, 0.45, 0.6 and 0.75 mg/kg) for a duration of 4 weeks. Afterwards, micro-CT was performed on the cross-sectional area of femur tissues. Subsequent results illustrated that bone loss in the femur of mice was increased (Fig. 2A), and BMD and BMC were gradually decreased in response to increased concentration of Risperidone, with the lowest BMD and BMC obtained at a concentration of 0.75 mg/kg (Fig. 2B, C). Meanwhile, the results of HE staining analysis demonstrated that trabecular bone was lost and destroyed in femur tissues of mice treated with Risperidone, in addition to increased apoptosis of osteoblasts (Fig. 2D).

Additionally, we assessed the effect of Risperidone on autophagy in femur tissues of mice. A Western blot analysis was performed to determine the expression patterns of autophagy-related proteins (LC3 II/I, Beclin1, and p62) in the femur tissues of mice. The obtained results showed that with an increase in Risperidone concentration, the protein expression levels of LC3 II/I and Beclin1 were all reduced and those of p62 were significantly elevated in the femur tissues of mice (Fig. 2E), indicating that Risperidone could inhibit autophagy in the femur tissues.

Additionally, we further detected the expression patterns of apoptosis-related proteins (cleaved PARP1, cleaved caspase-3, cleaved caspase-8 and cleaved caspase-9) to evaluate the effect of Risperidone on cell apoptosis in femur tissues of mice. Subsequent results illustrated that increasing the Risperidone concentration elevated the levels of cleaved PARP1, cleaved caspase-3, cleaved caspase-8 and cleaved caspase-9 in the femur tissues of mice, with the maximum attained at a dosage of 0.75 mg/kg Risperidone (Fig. 2F).

To further clarify the types of cells that affect autophagy and apoptosis, we detected the co-localization of LC3 II/I, Beclin1, and p62 with the osteoblast marker RUNX2 by means of double-labeled immunofluorescence. The obtained results illustrated that increasing the Risperidone concentration resulted in fewer LC3II/I^+^ and Beclin1^+^ osteoblasts (as RUNX^+^), but elevated p62^+^ proportion (Fig. 2G).

Taken together, these findings indicated that Risperidone repressed autophagy and promoted apoptosis of osteoblasts in femur tissues. When the concentration reached 0.75 mg/kg, the expression levels of the apoptosis-related proteins were the highest, while those of BMD and BMC as well as autophagy-related proteins were the lowest. Therefore, a dosage of 0.75 mg/kg of Risperidone was used for subsequent experimentation.

### Risperidone inhibits differentiation and autophagy of MC3T3-E1 cells while enhancing their apoptosis

**Fig. 3:**
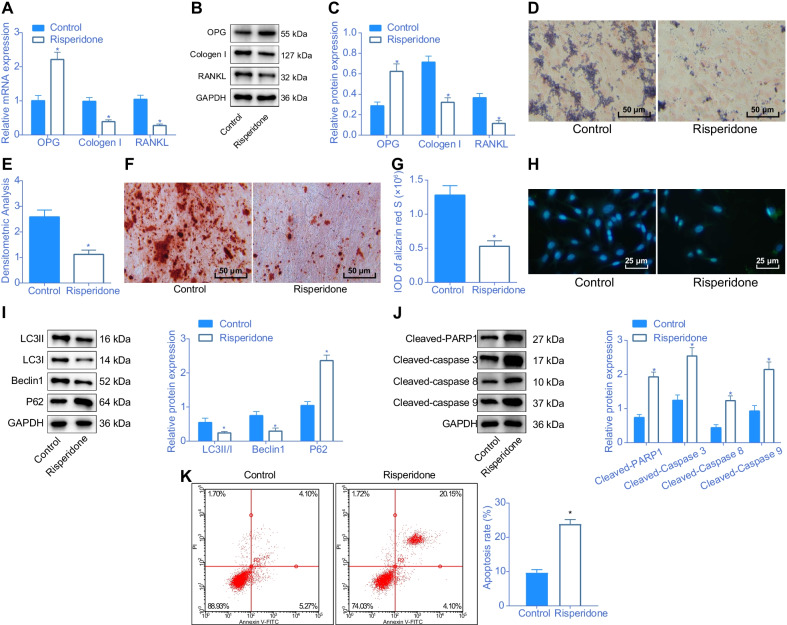
Risperidone confers an inhibitory effect on differentiation and autophagy and a promotive effect on apoptosis of osteoblasts in vitro. **A** the mRNA expression of OPG, collagen I and RANKL in MC3T3-E1 cells determined by RT-qPCR. **B**–**C** Western blot analysis of differentiation-related protein (OPG, collagen I and RANKL) expression in MC3T3-E1 cells. The band intensity was assessed. **D** representative images of ALP staining in MC3T3-E1 cells. E, statistical results of ALP positive rate in MC3T3-E1 cells. **F** representative images of alizarin red S staining in MC3T3-E1 cells. **G** statistical results of integrated optical density (IOD) of alizarin red S in MC3T3-E1 cells. **H** representative images of autophagosome in MC3T3-E1 cells assessed by immunofluorescence. **I** Western blot analysis of autophagy-related protein (LC3 II/I, Beclin1, and p62) expression in MC3T3-E1 cells. The band intensity was assessed. **J** Western blot analysis of apoptosis-related protein (cleaved PARP1, cleaved caspase-3, cleaved caspase-8 and cleaved caspase-9) expression in MC3T3-E1 cells. **K** apoptosis rate in MC3T3-E1 cells evaluated by flow cytometry. *p < 0.05, compared with MC3T3-E1 cells treated with normal culture medium. The cell experiments are repeated 3 times

To further investigate the effect of Risperidone on osteoblast differentiation, we carried out a series of in vitro experiments, including RT-qPCR, Western blot analysis, ALP staining and alizarin red S staining, to detect the expression patterns of differentiation-related proteins (OPG, collagen I and RANKL), ALP positive rate and cell differentiation, respectively. Subsequent results illustrated that Risperidone could induce an increase of OPG expression and decreases in the collagen I and RANKL expression levels, ALP positive rate and cell differentiation (Fig. 3A–G), indicating that Risperidone could inhibit the differentiation of MC3T3-E1 cells.

Additionally, to further evaluate the effect of Risperidone on the autophagy of MC3T3-E1 cells, immunofluorescence and Western blot analysis were performed to detect the formation of autophagosome and the protein expression patterns of LC3 II/I, Beclin1, and p62. The obtained results demonstrated that Risperidone significantly increased the levels of p62 in autophagosomes and proteins and markedly decreased those of LC3 II/I and Beclin1 (Fig. 3H, I), altogether indicating that Risperidone could suppress the autophagy of MC3T3-E1 cells.

Moreover, a Western blot analysis and flow cytometer were carried out to further examine the effect of Risperidone on apoptosis of MC3T3-E1 cells. It was observed that Risperidone could evidently elevate the levels of cleaved PARP1, cleaved caspase-3, cleaved caspase-8, and cleaved caspase-9 and increase the apoptosis rate of MC3T3-E1 cells (Fig. 3J, K). Taken together, these findings suggested that Risperidone repressed the differentiation and autophagy and promoted the apoptosis of osteoblasts.

### Risperidone promotes the expression of TNF-α to inhibit SATB2 expression in osteoblasts

**Fig. 4:**
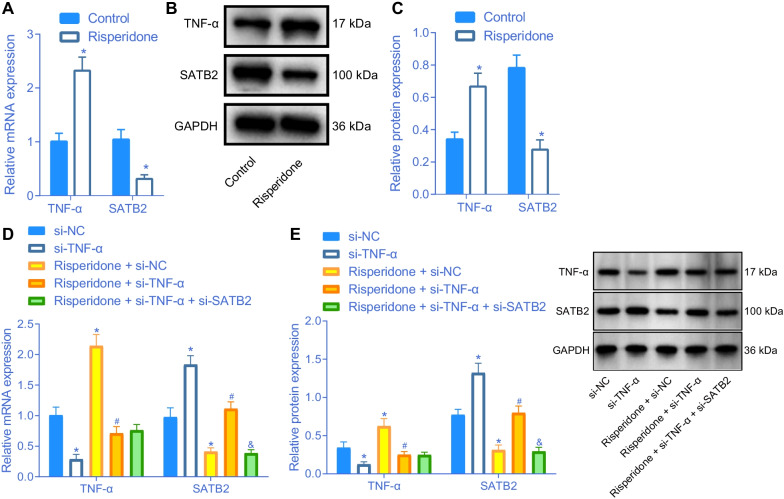
Risperidone induces an increase of TNF-α expression and a decrease of SATB2 expression. **A** relative mRNA expression of TNF-α and SATB2 determined by RT-qPCR in control and Risperidone-treated MC3T3-E1 cells. **B**–**C** Western blot analysis of TNF-α and SATB2 protein expression in control and Risperidone-treated MC3T3-E1 cells. The band intensity was assessed. **D** relative mRNA expression of TNF-α and SATB2 was detected by RT-qPCR in MC3T3-E1 cells. **E** Western blot analysis of TNF-α and SATB2 protein expression in MC3T3-E1 cells. *p < 0.05, compared with control cells or MC3T3-E1 cells treated with si-NC. ^#^*p* < 0.05, compared with MC3T3-E1 cells treated with Risperidone + si-NC. ^&^*p* < 0.05, compared with MC3T3-E1 cells treated with Risperidone + si-TNF-α. The experiments are repeated 3 times

Additionally, we carried out RT-qPCR and Western blot analysis to determine the expression of TNF-α and SATB2 in MC3T3-E1 cells treated with Risperidone, in an effort to evaluate whether Risperidone affected the expressions of TNF-α and SATB2. Subsequent results illustrated that the expression levels of TNF-α were elevated, while those of SATB2 were decreased in MC3T3-E1 cells after Risperidone treatment (Fig. 4A–C), indicating that Risperidone could enhance the expression of TNF-α and inhibit the expression of SATB2.

To further evaluate the effects of Risperidone, TNF-α, and SATB2 on osteoblasts, MC3T3-E1 cells were transduced with lentivirus and treated with Risperidone. According to the results of RT-qPCR and Western blot analysis, the expression levels of TNF-α were decreased, while those of SATB2 were increased following transduction with lentivirus expressing si-TNF-α. Meanwhile, treatment with Risperidone led to increased TNF-α expression and reduced SATB2 expression levels. However, TNF-α silencing was found to counter the reduction of SATB2 expression induced by Risperidone treatment. Additionally, SATB2 was found to be down-regulated by lentivirus expressing si-SATB2 in Risperidone-treated TNF-α-deficient MC3T3-E1 cells (Fig. 4D, E). Altogether, these findings indicated that Risperidone could increase the expression of TNF-α and inhibit the expression of SATB2 in MC3T3-E1 cells.

### Risperidone inhibits osteoblast differentiation through the TNF-α/SATB2 axis

**Fig. 5:**
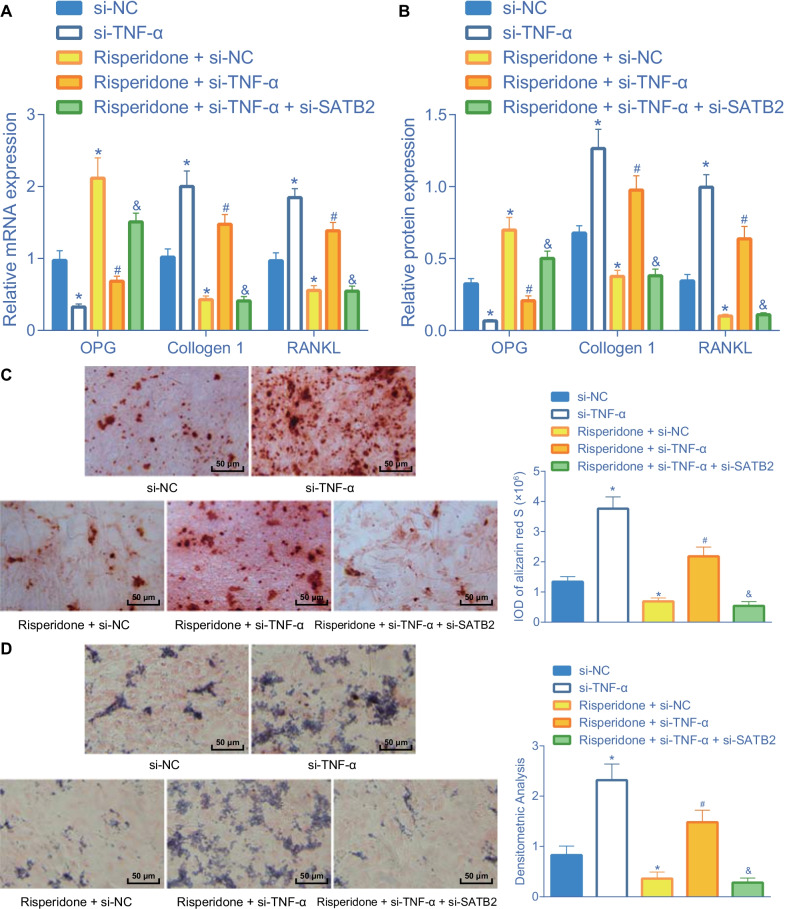
Risperidone inhibits osteoblast differentiation through the TNF-α/SATB2 axis. **A** the mRNA expression of OPG, collagen I and RANKL in MC3T3-E1 cells determined by RT-qPCR. **B** Western blot analysis of OPG, collagen I and RANKL expression in MC3T3-E1 cells. The protein band was assessed. **C** repesentative images and statistical analysis of positive Alizarin red S staining in MC3T3-E1 cells. **D** repesentative images and statistical analysis of positive ALP staining in MC3T3-E1 cells. *p < 0.05, compared with MC3T3-E1 cells treated with si-NC. ^#^*p* < 0.05, compared with MC3T3-E1 cells treated with Risperidone + si-NC. ^&^*p* < 0.05, compared with MC3T3-E1 cells treated with Risperidone + si-TNF-α. The cell experiments are repeated 3 times

We further sought to further verify the influence of Risperidone through the TNF-α/SATB2 axis on osteoblast differentiation. The results of RT-qPCR and Western blot analysis showed that the mRNA and protein expression levels of OPG were decreased, while those of collagen I and RANKL were elevated following TNF-α silencing. In addition, individual treatment with Risperidone brought about promotion of OPG mRNA and protein expression and inhibition of collagen I and RANKL mRNA and protein expression levels, the effect of which was abolished by additional TNF-α silencing. Meanwhile, additional SATB2 silencing also induced the promotion of OPG expression and inhibition of collagen I and RANKL expression levels in the Risperidone-treated TNF-α-deficient MC3T3-E1 cells (Fig. 5A, B, Additional file 1: Fig. S1A).

Additionally, the results of alizarin red S staining illustrated that treatment with si-TNF-α increased the alizarin red S-positive cells, while the treatment with Risperidone decreased the alizarin red S-positive cells, suggesting that Risperidone inhibited the differentiation of osteoblasts and reduced the deposition of calcified matrix. Moreover, combined treatment with Risperidone and si-TNF-α was found to increase the alizarin red S-positive cells, which could be diminished upon further SATB2 silencing (Fig. 5C). Furthermore, analysis using the ALP staining revealed an increase in ALP-positive cells upon treatment of si-TNF-α, while undergoing a reduction of ALP-positive cells upon Risperidone treatment. Meanwhile, combined treatment with Risperidone and si-TNF-α resulted in increased ALP-positive cells, while SATB2 silencing countered this trend (Fig. 5D). Collectively, these findings indicated that Risperidone could promote the TNF-α expression and repress SATB2 expression, thus inhibiting the differentiation of MC3T3-E1 cells.

### Risperidone represses autophagy and promotes apoptosis of osteoblasts via the TNF-α/SATB2 axis

**Fig. 6:**
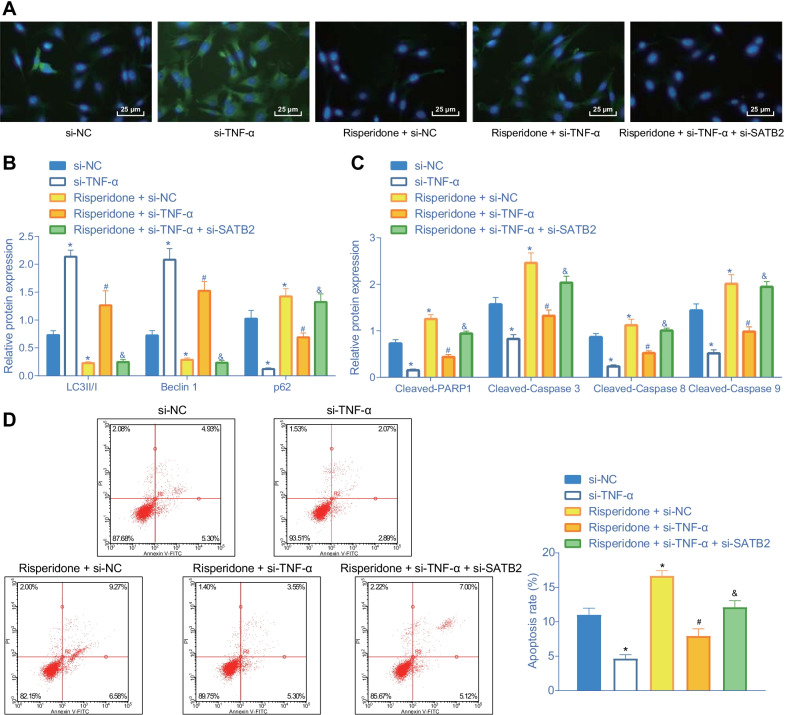
Risperidone inhibits autophagy and accelerates apoptosis of osteoblasts via the TNF-α/SATB2 axis. **A** representative images of autophagosome in MC3T3-E1 cells assessed by immunofluorescence. **B** Western blot analysis of autophagy-related protein (LC3 II/I, Beclin1, and p62) expression in MC3T3-E1 cells. The band intensity was quantified. **C** Western blot analysis of apoptosis-related proteins (cleaved PARP1, cleaved caspase-3, cleaved caspase-8 and cleaved caspase-9). The protein band was quantified. **D** apoptosis rate of MC3T3-E1 cells determined by flow cytometry. *p < 0.05, compared with MC3T3-E1 cells treated with si-NC. ^#^*p* < 0.05, compared with MC3T3-E1 cells treated with Risperidone + si-NC. ^&^*p* < 0.05, compared with MC3T3-E1 cells treated with Risperidone + si-TNF-α. The experiments are repeated 3 times

Additionally, to verify whether Risperidone, TNF-α, and SATB2 affected autophagy and apoptosis of MC3T3-E1 cells, we carried out a series of in vitro experiments including immunofluorescence, Western blot analysis and flow cytometry to determine the number of autophagosome and the expression patterns of autophagy-related proteins and apoptosis-related proteins, respectively. Initially, the results of immunofluorescence showed that the number of autophagosome was decreased following the introduction of Risperidone and si-NC, suggesting that Risperidone could inhibit the autophagy of MC3T3-E1 cells. However, silencing of TNF-α was found to rescue the formation of autophagosome suppressed by Risperidone, while this trend could be negated upon additional silencing of SATB2 (Fig. 6A).

In addition, the result of Western blot analysis showed that the introduction of Risperidone and si-NC decreased the protein levels of LC3 II/I and Beclin1 and increased those of p62 relative to the matched controls, suggesting that Risperidone could inhibit the autophagy of MC3T3-E1 cells. Meanwhile, TNF-α silencing elevated the protein levels of LC3 II/I and Beclin1 and reduced the expression levels of p62. Furthermore, additional TNF-α silencing counteracted the effects of Risperidone treatment, while SATB2 knockdown brought about decreased protein levels of LC3 II/I and Beclin1 and increased protein levels of p62 in the Risperidone-treated TNF-α-deficient MC3T3-E1 cells (Fig. 6B, Additional file 1: Fig. S1B), which indicated that Risperidone induced TNF-α up-regulation and SATB2 down-regulation, resulting in decreased autophagy of MC3T3-E1 cells.

Additional Western blot analysis illustrated that the protein expression levels of cleaved PARP1, cleaved caspase-3, cleaved caspase-8, and cleaved caspase-9 were all diminished following the introduction of si-TNF-α, while the opposing trends were documented upon Risperidone treatment. However, the protein expression levels of cleaved PARP1, cleaved caspase-3, cleaved caspase-8 and cleaved caspase-9 were found to be down-regulated in response to both Risperidone treatment and TNF-α silencing as compared to Risperidone treatment alone, while a promotion in the aforementioned levels was observed upon further SATB2 silencing (Fig. 6C, Additional file 1: Fig. S1C). Meanwhile, flow cytometric analysis of apoptotic cells also revealed consistent results (Fig. 6D). Taken together, these findings indicated that Risperidone induced TNF-α expression to repress the expression of SATB2, thus promoting the apoptosis of MC3T3-E1 cells.

### Risperidone induces TNF-α expression and thus represses SATB2 in vivo

**Fig. 7:**
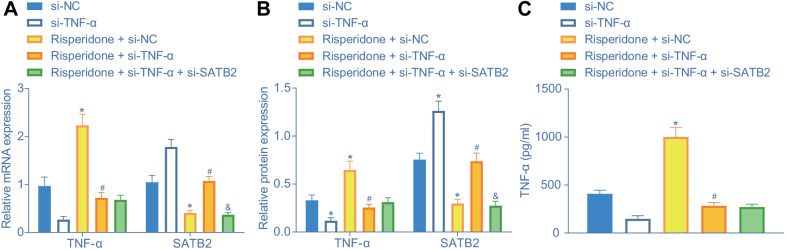
Risperidone promotes TNF-α expression to inhibit SATB2 expression in vivo*.* A, the mRNA expression of TNF-α and SATB2 in femur tissues of mice determined by RT-qPCR. B, Western blot analysis of TNF-α and SATB2 expression in femur tissues of mice. The band intensity was quantified. C, the expression of TNF-α in the serum of mice was evaluated by ELISA. *p < 0.05, compared with mice introduced with lentivirus expressing si-NC. ^#^*p* < 0.05, compared with mice introduced with cRisperidone + lentivirus expressing si-NC. ^&^*p* < 0.05, compared with mice introduced with Risperidone + lentivirus expressing si-TNF-α. The experiments are repeated 3 times *n* = 6

Furthermore, we validated the in vitro effect of Risperidone on the TNF-α on SATB2 in vivo. The results of RT-qPCR and Western blot analysis showed that treatment with lentivirus expressing si-TNF-α successfully decreased the expression levels of TNF-α, while increasing those of SATB2. Conversely, the introduction of Risperidone brought about the opposite trends. Additionally, combined treatment with Risperidone and lentivirus expressing si-TNF-α induced a decline in TNF-α expression and an increase in SATB2 expression levels, and this could be reversed upon further treatment with lentivirus expressing si-SATB2 (Fig. 7A, B, Additional file 1: Fig. S1D) Meanwhile, the results of TNF-α expression patterns determined by ELISA (Fig. 7C) was consistent with those of RT-qPCR and Western blot analysis. Together, these findings suggested that Risperidone promoted TNF-α expression and inhibited SATB2 expression in femur tissues of mice.

### Risperidone inhibits differentiation and autophagy while promoting apoptosis via the TNF-α/SATB2 axis in femur tissues of mice in vivo

**Fig. 8:**
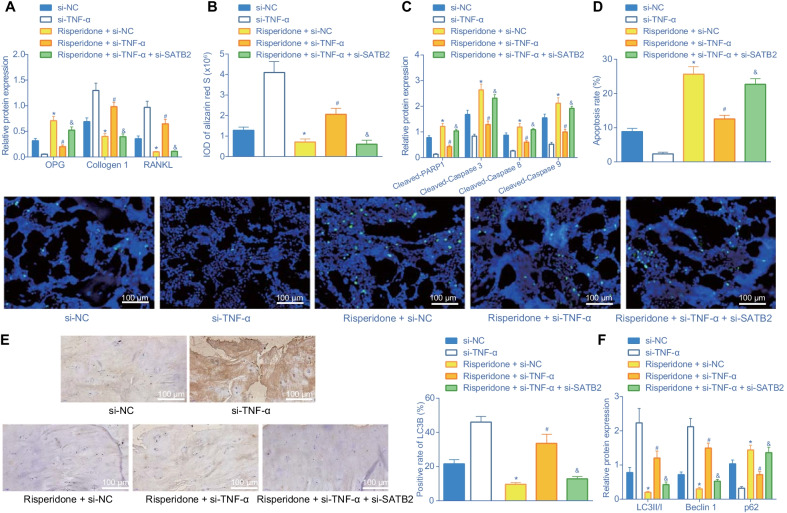
Risperidone represses differentiation and autophagy while enhancing apoptosis via the TNF-α/SATB2 axis in vivo. A, Western blot analysis of differentiation-related proteins (OPG, collagen I and RANKL) expression in femur tissues of mice. The band intensity was assessed. B, statistical results of alizarin red S staining in femur tissues of mice. C, Western blot analysis of apoptosis-related proteins (cleaved PARP1, cleaved caspase-3, cleaved caspase-8 and cleaved caspase-9) in femur tissues of mice. The protein band was assessed. D, apoptosis rate in femur tissues in mice assessed by TUNEL staining. E, representative images of femur tissues in mice stained by immunohistochemistry and statistical results of LC3B positive rate. F, Western blot analysis of autophagy-related proteins (LC3 II/I, Beclin1, and p62) in femur tissues of mice. The band intensity was quantified and analyzed. *p < 0.05, compared with mice introduced with lentivirus expressing si-NC. ^#^*p* < 0.05, compared with mice introduced with Risperidone + lentivirus expressing si-NC. ^&^*p* < 0.05, compared with mice introduced with Risperidone + lentivirus expressing si-TNF-α. The experiments are repeated 3 times *n* = 6

Lastly, to verify the influence of Risperidone on differentiation, autophagy and apoptosis in vivo, we treated mice with Risperidone, lentivirus expressing si-TNF-α and lentivirus expressing si-SATB2 either alone or in combination. Subsequently, a panel of experiments including Western blot analysis, alizarin red S staining, TUNEL, and immunohistochemistry were carried out to evaluate the differentiation, autophagy and apoptosis in femur tissues of mice, respectively. The results of Western blot analysis showed that Risperidone treatment elevated the OPG protein expression and down-regulated the collagen I and RANKL protein expression levels, while combined treatment with Risperidone and TNF-α silencing countered the effect of Risperidone treatment alone. Additionally, OPG protein expression levels were found to be up-regulated, while collagen I and RANKL protein expression levels were down-regulated in femur tissues of Risperidone-treated mice upon simultaneous silencing of TNF-α and SATB2 versus those upon individual TNF-α silencing (Fig. 8A), Besides, the results of alizarin red S were consistent with those of Western blot analysis. More red fat droplet represented better lipogenesis, while less fat droplet represented less osteogenesis (Fig. 8B, Additional file 1: Fig. S1E), indicating that Risperidone could augment the expression of TNF-α and inhibit SATB2 expression, thus suppressing the differentiation of mouse femur tissues.

Furthermore, the results of Western blot analysis and TUNEL revealed that Risperidone treatment elevated the protein expression levels of cleaved PARP1, cleaved caspase-3, cleaved caspase-8, and cleaved caspase-9 as well as apoptosis rate in femur tissues of mice, while these trends were abrogated upon combined treatment with Risperidone and TNF-α silencing. Additionally, simultaneous silencing of TNF-α and SATB2 augmented the expression levels of apoptosis-related proteins along with apoptosis rate in femur tissues of Risperidone-treated mice compared to individual TNF-α silencing (Fig. 8C, D, Additional file 1: Fig. S1F). Moreover, the results of Western blot analysis showed that the treatment of Risperidone increased the protein expression levels of p62, and decreased those of LC3 II/I and Beclin1 in femur tissues of mice. However, additional TNF-α silencing negated the aforementioned effect of Risperidone treatment. In response to simultaneous silencing of TNF-α and SATB2, the femur tissues of Risperidone-treated mice exhibited a decline in protein expression levels of LC3 II/I and Beclin1, yet an enhancement in those of p62. Besides, the results of LC3 immunohistochemistry (Fig. 8E) were consistent with those of Western blot analysis (Fig. 8F, Additional file 1: Fig. S1G).

Collectively, these findings indicated that Risperidone elevated TNF-α expression, and inhibited SATB2 expression, thus repressing the differentiation and autophagy and promoting the apoptosis of femur tissues in mice.

## Discussion

Osteoblasts are well-known to play critical roles in the process of bone formation, while a lack of osteoblasts is associated with damaged fracture repair (Dirckx et al. 2013). Meanwhile, autophagy in osteoblasts is known to induce morphological changes linked to osteocyte formation as well as skeletal homeostasis (Piemontese et al. 2016). What’s interesting is that Risperidone, an antipsychotic, has attracted much attention due to the possibility that it can induce osteoporosis. Moreover, Risperidone is used as a neuroleptics for depressed patients, however, it’s long-term use may cause BMD loss (Laekeman et al. 2008). In lieu of this, the current study set out to explore the unfounded role of Risperidone in osteoblast functions and cellular mechanism. Consequently, the obtained findings revealed that Risperidone inhibited autophagy and differentiation and promoted apoptosis of osteoblasts by enhancing TNF-α and repressing SATB2.

Initially, our findings revealed that Risperidone augmented TNF-α levels to repress the expression of the SATB2 protein in osteoblasts. Similarly, Chen et al*.* documented that Risperidone elevated the production of TNF-α and further led to cell apoptosis directed by TNF-α in neutrophils (Chen et al. 2012). More recently, another study illustrated the ability of TNF-α to suppress osteoblast differentiation and trigger osteoclast differentiation (Wang et al. 2016). Furthermore, an investigation by Zuo et al*.* demonstrated that TNF-α exerted its repressive role in osteoblast differentiation by decreasing the expression SATB2 via the NF-κB and MAPK pathways (Zuo et al. 2018). Also, in line with our findings, another study previously indicated that TNF-α diminishes the expression of SATB2 by rescuing the microRNA-33a-5p expression, thus inhibiting osteogenic differentiation in human BMSCs (Mi et al. 2016). In addition, TNF-α can reduce the expression of SATB2 and RUNX2 and promote osteogenic differentiation of BMSCs by decreasing microRNA-31 expression in ethanol-induced osteonecrosis (Yu et al. 2019). Collectively, these findings and data support that Risperidone elevated TNF-α levels to decrease the SATB2 expression.

Subsequent experimentation in our study further illustrated that Risperidone elevated the protein expressions of OPG, p62, cleaved PARP1, cleaved caspase-3, cleaved caspase-8, and cleaved caspase-9, and decreased those of LC3 II/I, Beclin1, collagen I and RANKL, all of which resulted in retarded autophagy and differentiation and enhanced apoptosis of osteoblasts. There is a plethora of evidence that highlights of association of Cleaved PARP1, cleaved caspase-3, cleaved caspase-8, and cleaved caspase-9 with the apoptosis of cells (Casao et al. 2015; Du et al. 2017). Moreover, elevated cleaved caspase-3 and cleaved caspase-9 were previously illustrated to be associated with osteoblast death induced by alkylphenol through the apoptotic pathway dependent in mitochondria (Sabbieti et al. 2011). Cleaved caspase-3 and PARP are recognized as substrates of caspase-3, which plays a key role in MIN6 cell apoptosis mediated by IFN-γ and TNF-α through the NF-κB and Bcl-2 pathways (Cao et al. 2013). On the other hand, LC3, Beclin1, and p62 are well-established as proteins related to cell autophagy (Masuda et al. 2016). In addition, another study has found that the expression levels of LC3 and p62 are associated with the autophagy and differentiation of osteoblasts (Ha et al. 2014). Meanwhile, OPG is regarded as a cytokine associated with the modulation of interaction between osteoblast and osteoclast and bone mass maintenance (Mrak et al. 2007), whereas the expression of RANKL was previously illustrated to be linked to human osteoblast differentiation (Atkins et al. 2003). Besides, a prior study has indicated that both OPG and RANKL exert a prominent effect on the process of bone remodeling (Jurado et al. 2010). Consistently, our findings indicate that Risperidone inhibited the autophagy and differentiation and promoted the apoptosis of osteoblasts.

Furthermore, our study also provided evidence demonstrating that Risperidone repressed the differentiation and autophagy and enhanced the apoptosis of osteoblasts by increasing TNF-α levels and decreasing the expression of SATB2. It is also noteworthy that the study performed by Jones et al*.* suggested that Risperidone was associated with the progression of osteoporosis (Jones 2014). In addition, Risperidone is known to contribute to BMD reduction in female patients with premenopausal schizophrenia (Becker et al. 2003). Similarly, Motyl et al*.* illustrated that Risperidone treatment caused negative skeletal reactions through indirect non-cell autonomous effects and a direct increase of osteoclast activity (Motyl et al. 2012). Meanwhile, a prior study illustrated that TNF-α was capable of inducing apoptosis of osteoblast-like MG-63 cells in high-glucose conditions (Sun et al. 2016). Besides, TNF-α was previously documented to exert a suppressive effect on osteoblast differentiation induced by bone morphogenetic protein through the activation of the SAPK/JNK signaling pathway (Mukai et al. 2007). The third focus of our study, the SATB protein is heralded as a critical regulator of osteoblast differentiation and bone generation (Kim et al. 2012). Moreover, SATB2 silencing was recently suggested to inhibit osteogenic differentiation and down-regulate autophagy-related genes in BMSCs (Dong et al. 2015). In addition, a prior study uncovered that depletion of SATB2 promoted the menadione-induced apoptosis of osteoblast-like MG63 cells (Wei et al. 2012). More recently, TNF-α was revealed to induce the inhibition of SATB2 and RUNX2 expression through microRNA-31 whereby inhibiting the osteogenic differentiation of BMSCs in ethanol-induced osteonecrosis (Yu et al. 2019). Additionally, TNF-α and SATB2 depletion can depresses osteogenic differentiation in human BMSCs under the regulation of microRNA-33a-5p (Mi et al. 2016). Consistent with these evidences, our findings indicated that Risperidone repressed the differentiation of osteoblasts by enhancing TNF-α and suppressing SATB2. However, in-depth investigation is warranted in the future for verification of the direct or indirect mechanism by which TNF-α mediates SATB2.

## Conclusion

**Fig. 9:**
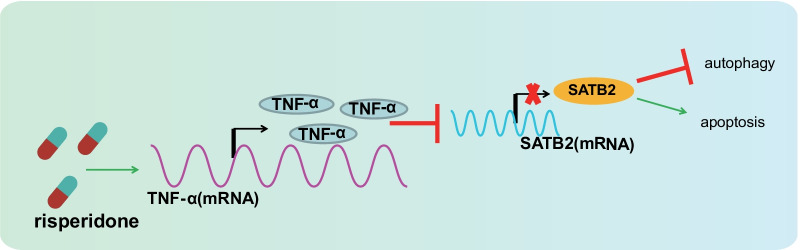
Schematic diagram of the mechanism by which Risperidone affects the autophagy and apoptosis of osteoblasts. Risperidone promotes TNF-α expression to represses SATB2 expression, thus inhibiting autophagy and enhancing apoptosis of osteoblasts, ultimately arresting osteoblast differentiation

In summary, we uncovered that Risperidone enhanced TNF-α levels and weakened the SATB2 expression, repressing autophagy and accelerating the apoptosis of osteoblasts in the process, and eventually preventing the differentiation of osteoblasts (Fig. 9). Our findings shed a new light on the involvement and mechanism of Risperidone in osteoblast differentiation and provided theoretical basis for the potential mechanism underlying osteoblast differentiation mediated by antipsychotic drugs.

Nevertheless, our study merely incorporated cell and mouse models, while the role and mechanism of Risperidone in clinic settings remains to be well-elucidated. In future, clinical trials with specimens from patients with bone diseases should be performed to validate our findings. Besides, the underlying mechanism of TNF-α inhibiting the expression of SATB2 and whether it acted through a certain mechanism or via direct regulation requires further elaboration. Longer-term studies are also required to assess the adverse effects and metabolic changes associated with Risperidone therapy as long-term use of Risperidone or high concentration of Risperidone may cause osteoporosis.

## Supplementary Information

**Additional file 1: Fig. S1** Representative images of Western blots. **A** representative Western blot images of OPG, collagen I and RANKL proteins in MC3T3-E1 cells. **B** representative Western blot images of autophagy-related protein (LC3 II/I, Beclin1, and p62) in MC3T3-E1 cells. **C** representative Western blot images of apoptosis-related proteins (cleaved PARP1, cleaved caspase-3, cleaved caspase-8 and cleaved caspase-9). **D** representative Western blot images of TNF-α and SATB2 proteins in femur tissues of mice. **E** representative Western blot images of differentiation-related proteins (OPG, collagen I and RANKL) in femur tissues of mice. **F** representative Western blot images of apoptosis-related proteins (cleaved PARP1, cleaved caspase-3, cleaved caspase-8 and cleaved caspase-9) in femur tissues of mice. G, representative Western blot images of autophagy-related proteins (LC3 II/I, Beclin1, and p62) in femur tissues of mice.

**Additional file 2: Table S1.** siRNA sequences for TNF-α and SATB2 knockdown.

**Additional file 3:** **Table S2.** Primer sequences for RT-qPCR

**Additional file 4: Table S3. **Gene co-expression scores of 62 genes obtained through the GeneMANIA website.

## Data Availability

The datasets generated or analyzed during the current study are available upon reasonable request from the corresponding author.

## Abbreviations

TNF-α: Tumor necrosis factor-α
SATB2: Sequence-binding protein
CT: Micro-computed tomography
BVF: Bone volume fraction
TbTh: Trabecular thickness
TbN: Trabecular number
TbPf: Trabecular bone mode factor
BSA: Bone surface area
BMC: Bone mineral content
BMD: Bone mineral density
HE: Hematoxylin–eosin
EDTA: Ethylene diamine tetraacetic acid
DAB: 3,3'-Diaminobenzidine tetrahydrochloride
GAPDH: Glyceraldehyde-3-phosphate dehydrogenase
NC: Negative control
ATCC: American Type Culture Collection
MEM: α-Minimal essential medium
FBS: Fetal bovine serum
BCA: Bicinchoninic acid
BSA: Bovine serum albumin
LC3: Light chain 3
PARP1: Poly(ADP-ribose) polymerase 1
CST: Cell Signaling Technologies
OPG: Osteoprotegerin
HRP: Horsereddish peroxidase
RANKL: Receptor activator of nuclear factor (NF)-kB-ligand
si/siRNA: Small interfering RNA
M-CSF: Macrophage colony-stimulating factor
TRAP: Tartaric acid phosphatase
HEPES: N-2-hydroxyethyl-piperazine-N'-2-ethanesulfonic acid
RT-qPCR: Reverse transcription quantitative polymerase chain reaction
cDNA: Complementary DNA
TUNEL: Terminal deoxynucleotidyl transferase-mediated dUTP-biotin nick end labeling
DMSO: Dimethyl sulfoxide
ALP: Alkaline phosphatase
PBS: Phosphate buffer saline
TBST: Tris-buffered saline with Tween-20
FITC: Fluorescein isothiocyanate
PI: Propidium iodide
ABF: Antimicrobial-free
DAPI: 4',6-Diamidino-2-phenylindole
ELISA: Enzyme-linked immunosorbent assay
TMB: Tetramethylbenzidine
ANOVA: Analysis of variance
