# Supplementary material for: Involvement of the TNF-α/SATB2 axis in the induced apoptosis and inhibited autophagy of osteoblasts by the antipsychotic Risperidone
Source: Mol Med. 2022 May 3;28:46. doi: 10.1186/s10020-022-00466-9 (PMC9066868; doi:10.1186/s10020-022-00466-9)
Supplement: Supplementary file 2 — Additional file 2: Table S1. siRNA sequences for TNF-α and SATB2 knockdown. [file 10020_2022_466_MOESM2_ESM.docx]

**Additional file 2: Table S1** siRNA sequences for TNF-α and SATB2 knockdown

| Gene | Sequence |
| --- | --- |
| si-NC | 5'-UUCUCCGAACGUGUCACGUTT-3' |
| si-SATB2-1 | 5'-GUCAGAGAUGAGCUGAAGATT-3' |
| si-SATB2-2 | 5'-CCAAACACACCAUCAUCAAGU-3' |
| si-TNF-α-1 | 5′-GACAACCAACTAGTGGTGC-3′ |
| si-TNF-α -2 | 5’-CACGTTTTCCGTGAAAACAGAGC-3’ |
