# Supplementary material for: Involvement of the TNF-α/SATB2 axis in the induced apoptosis and inhibited autophagy of osteoblasts by the antipsychotic Risperidone
Source: Mol Med. 2022 May 3;28:46. doi: 10.1186/s10020-022-00466-9 (PMC9066868; doi:10.1186/s10020-022-00466-9)
Supplement: Supplementary file 3 — Additional file 3: Table S2. Primer sequences for RT-qPCR [file 10020_2022_466_MOESM3_ESM.docx]

**Additional file 3: Table S2** Primer sequences for RT-qPCR

| Gene | Sequence (5’-3’) |
| --- | --- |
| TNF-α | F: CAGGCGGTGCCTATGTCTC |
|  | R: CGATCACCCCGAAGTTCAGTAG |
| SATB2 | F: CCGCAGGCAGAGCAATAGAT |
|  | R: TGTGCAACAGAGGAATGCCA |
| OPG | F: CCTTGCCCTGACCACTCTTAT |
|  | R: CACACACTCGGTTGTGGGT |
| collagen I | F: TGGCAAGAATGGCGACC |
|  | R: ACCGTTGAGTCCATCTTTGC |
| RANKL | F: AGCCGAGACTACGGCAAGTA |
|  | R: AAAGTACAGGAACAGAGCGATG |
| GAPDH | F: ACCACAGTCCATGCCATCAC |
|  | R: TCCACCACCCTGTTGCTGTA |

Notes: F, forward; R, reverse; RT-qPCR, reverse transcription quantitative polymerase chain reaction; TNF-α, tumor necrosis factor-α; SATB2, special AT-rich sequence-binding protein; OPG, osteoprotegerin; RANKL, receptor activator of nuclear factor-kappaB ligand; GAPDH, glyceraldehyde-3-phosphate dehydrogenase.
