# Supplementary material for: Involvement of the TNF-α/SATB2 axis in the induced apoptosis and inhibited autophagy of osteoblasts by the antipsychotic Risperidone
Source: Mol Med. 2022 May 3;28:46. doi: 10.1186/s10020-022-00466-9 (PMC9066868; doi:10.1186/s10020-022-00466-9)
Supplement: Supplementary file 4 — Additional file 4: Table S3. Gene co-expression scores of 62 genes obtained through the GeneMANIA website. [file 10020_2022_466_MOESM4_ESM.docx]

**Supplementary Table 3** Gene co-expression scores of 62 genes obtained through the GeneMANIA website

| Symbol | Description | Score | Functions | Links |
| --- | --- | --- | --- | --- |
| Satb2 |  | 0.8606418292722848 | cartilage development, chromatin, connective tissue development, embryonic organ morphogenesis, neuron migration, nuclear chromatin, nuclear chromosome part, ossification, osteoblast differentiation | http://www.ncbi.nlm.nih.gov/sites/entrez?db=gene&cmd=search&term=212712 |
| Atg4d |  | 0.8409043028896519 | autophagy, cysteine-type endopeptidase activity, cysteine-type peptidase activity, endopeptidase activity | http://www.ncbi.nlm.nih.gov/sites/entrez?db=gene&cmd=search&term=235040 |
| Dram1 |  | 0.8320744066115957 | execution phase of apoptosis | http://www.ncbi.nlm.nih.gov/sites/entrez?db=gene&cmd=search&term=71712 |
| Atg3 |  | 0.8172166502516648 | apoptotic mitochondrial changes, autophagic vacuole assembly, autophagy, cellular response to external stimulus, cellular response to extracellular stimulus, cellular response to nutrient levels, cellular response to starvation, macroautophagy, mitochondrial fission, mitochondrial fragmentation involved in apoptotic process, mitochondrion organization, organelle assembly, response to extracellular stimulus, response to nutrient levels, response to starvation, vacuole organization | http://www.ncbi.nlm.nih.gov/sites/entrez?db=gene&cmd=search&term=67841 |
| Optn |  | 0.7848249308937885 | cytokine secretion, establishment of protein localization to membrane, establishment of protein localization to organelle, Golgi organization, negative regulation of neuron apoptotic process, negative regulation of neuron death, neuron apoptotic process, neuron death, protein C-terminus binding, protein homooligomerization, protein localization to membrane, protein secretion, protein targeting, regulation of neuron apoptotic process, regulation of neuron death, single-organism cellular localization, single-organism localization | http://www.ncbi.nlm.nih.gov/sites/entrez?db=gene&cmd=search&term=71648 |
| Nlrp3 |  | 0.7670934441256259 | activation of cysteine-type endopeptidase activity, activation of cysteine-type endopeptidase activity involved in apoptotic process, acute inflammatory response, cellular protein complex assembly, cellular response to biotic stimulus, cellular response to lipid, cellular response to lipopolysaccharide, cellular response to molecule of bacterial origin, cytokine secretion, defense response to other organism, establishment of protein localization to organelle, I-kappaB kinase/NF-kappaB signaling, interleukin-1 beta production, interleukin-1 beta secretion, interleukin-1 production, interleukin-1 secretion, negative regulation of cytokine production, negative regulation of intracellular transport, negative regulation of secretion, NF-kappaB import into nucleus, nuclear import, positive regulation of cysteine-type endopeptidase activity, positive regulation of cysteine-type endopeptidase activity involved in apoptotic process, positive regulation of cytokine production, positive regulation of cytokine secretion, positive regulation of endopeptidase activity, positive regulation of interleukin-1 beta production, positive regulation of interleukin-1 beta secretion, positive regulation of interleukin-1 production, positive regulation of interleukin-1 secretion, positive regulation of NF-kappaB transcription factor activity, positive regulation of peptidase activity, positive regulation of protein secretion, positive regulation of protein transport, positive regulation of secretion, positive regulation of sequence-specific DNA binding transcription factor activity, protein import, protein import into nucleus, protein localization to nucleus, protein secretion, regulation of acute inflammatory response, regulation of cysteine-type endopeptidase activity, regulation of cysteine-type endopeptidase activity involved in apoptotic process, regulation of cytokine secretion, regulation of endopeptidase activity, regulation of I-kappaB kinase/NF-kappaB signaling, regulation of inflammatory response, regulation of interleukin-1 beta secretion, regulation of interleukin-1 secretion, regulation of intracellular protein transport, regulation of NF-kappaB import into nucleus, regulation of nucleocytoplasmic transport, regulation of protein import into nucleus, regulation of protein localization to nucleus, regulation of protein secretion, regulation of transcription factor import into nucleus, response to bacterium, response to lipopolysaccharide, response to molecule of bacterial origin, response to virus, single-organism nuclear import, transcription factor import into nucleus, zymogen activation | http://www.ncbi.nlm.nih.gov/sites/entrez?db=gene&cmd=search&term=216799 |
| Hspa4 |  | 0.7621218478250702 | activating transcription factor binding, cellular protein complex assembly, establishment of protein localization to membrane, establishment of protein localization to mitochondrion, establishment of protein localization to organelle, lipid particle, mitochondrial membrane organization, mitochondrial transport, mitochondrion organization, negative regulation of binding, negative regulation of DNA binding, negative regulation of phosphorylation, negative regulation of protein phosphorylation, positive regulation of binding, protein import, protein localization to membrane, protein targeting, regulation of binding, regulation of DNA binding, regulation of protein binding, RNA polymerase II transcription factor binding, single-organism cellular localization, single-organism localization | http://www.ncbi.nlm.nih.gov/sites/entrez?db=gene&cmd=search&term=15525 |
| Pik3cb |  | 0.7448663994419618 | 1-phosphatidylinositol-3-kinase activity, blood coagulation, calcium ion homeostasis, cell-cell adhesion, cell-matrix adhesion, cell-substrate adhesion, cellular calcium ion homeostasis, cellular divalent inorganic cation homeostasis, coagulation, glycerophospholipid biosynthetic process, hemostasis, phosphatidylinositol 3-kinase activity, phosphatidylinositol 3-kinase complex, phosphatidylinositol kinase activity, phosphatidylinositol phosphate kinase activity, phosphatidylinositol phosphorylation, phosphatidylinositol-3-phosphate biosynthetic process, regulation of body fluid levels, regulation of cell adhesion, regulation of cell-matrix adhesion, regulation of cell-substrate adhesion, wound healing | http://www.ncbi.nlm.nih.gov/sites/entrez?db=gene&cmd=search&term=74769 |
| Tnfsf11 |  | 0.7003871323552222 | acid secretion, activation of MAPK activity, activation of protein kinase activity, acute inflammatory response, acute-phase response, amide transport, anatomical structure homeostasis, bone remodeling, bone resorption, calcium ion homeostasis, carboxylic acid transport, cell chemotaxis, cell-cell adhesion, cellular response to tumor necrosis factor, cytokine activity, cytokine receptor binding, cytokine-mediated signaling pathway, epithelial cell proliferation, ERK1 and ERK2 cascade, fatty acid derivative transport, fatty acid transport, fever generation, gland development, heat generation, homotypic cell-cell adhesion, hormone secretion, hormone transport, I-kappaB kinase/NF-kappaB signaling, icosanoid secretion, icosanoid transport, JNK cascade, leukocyte chemotaxis, leukocyte migration, leukocyte proliferation, lipid transport, lymph node development, mammary gland alveolus development, mammary gland development, mammary gland epithelial cell proliferation, mammary gland epithelium development, mammary gland lobule development, monocarboxylic acid transport, multicellular organismal homeostasis, myeloid cell differentiation, myeloid leukocyte differentiation, myeloid leukocyte migration, organic acid transport, organic anion transport, ossification, osteoclast differentiation, peptide secretion, peptide transport, positive regulation of acute inflammatory response, positive regulation of cell activation, positive regulation of cell adhesion, positive regulation of cell-cell adhesion, positive regulation of defense response, positive regulation of ERK1 and ERK2 cascade, positive regulation of fatty acid transport, positive regulation of heat generation, positive regulation of homeostatic process, positive regulation of I-kappaB kinase/NF-kappaB signaling, positive regulation of icosanoid secretion, positive regulation of inflammatory response, positive regulation of ion transport, positive regulation of JUN kinase activity, positive regulation of leukocyte activation, positive regulation of leukocyte differentiation, positive regulation of lipid transport, positive regulation of lymphocyte activation, positive regulation of MAP kinase activity, positive regulation of myeloid cell differentiation, positive regulation of myeloid leukocyte differentiation, positive regulation of NF-kappaB transcription factor activity, positive regulation of organic acid transport, positive regulation of osteoclast differentiation, positive regulation of prostaglandin secretion, positive regulation of protein kinase B signaling, positive regulation of protein serine/threonine kinase activity, positive regulation of response to external stimulus, positive regulation of secretion, positive regulation of sequence-specific DNA binding transcription factor activity, positive regulation of T cell activation, positive regulation of tissue remodeling, prostaglandin secretion, prostaglandin transport, protein homooligomerization, protein kinase B signaling, regulation of acute inflammatory response, regulation of anion transport, regulation of bone remodeling, regulation of cell adhesion, regulation of cell-cell adhesion, regulation of ERK1 and ERK2 cascade, regulation of fatty acid transport, regulation of fever generation, regulation of heat generation, regulation of homeostatic process, regulation of homotypic cell-cell adhesion, regulation of hormone secretion, regulation of I-kappaB kinase/NF-kappaB signaling, regulation of icosanoid secretion, regulation of inflammatory response, regulation of JNK cascade, regulation of JUN kinase activity, regulation of leukocyte differentiation, regulation of lipid transport, regulation of lymphocyte activation, regulation of MAP kinase activity, regulation of myeloid cell differentiation, regulation of myeloid leukocyte differentiation, regulation of organic acid transport, regulation of osteoclast differentiation, regulation of peptide secretion, regulation of peptide transport, regulation of prostaglandin secretion, regulation of protein kinase B signaling, regulation of stress-activated MAPK cascade, regulation of stress-activated protein kinase signaling cascade, regulation of T cell activation, regulation of tissue remodeling, response to tumor necrosis factor, stress-activated MAPK cascade, stress-activated protein kinase signaling cascade, temperature homeostasis, tissue homeostasis, tissue remodeling, tumor necrosis factor receptor superfamily binding, tumor necrosis factor-mediated signaling pathway | http://www.ncbi.nlm.nih.gov/sites/entrez?db=gene&cmd=search&term=21943 |
| Ubc |  | 0.6997746929112189 | protease binding | http://www.ncbi.nlm.nih.gov/sites/entrez?db=gene&cmd=search&term=22190 |
| Dkk1 |  | 0.6922863772144472 | anterior/posterior pattern specification, appendage development, appendage morphogenesis, BMP signaling pathway, canonical Wnt signaling pathway, cardiac muscle cell differentiation, cardiac muscle tissue development, cardiocyte differentiation, cell fate commitment, cell fate specification, cell surface receptor signaling pathway involved in heart development, embryonic appendage morphogenesis, embryonic limb morphogenesis, endoderm development, epidermis development, forebrain development, hair cycle, hair cycle process, hair follicle development, limb development, limb morphogenesis, lipoprotein particle receptor binding, low-density lipoprotein particle receptor binding, molting cycle, molting cycle process, muscle cell differentiation, muscle organ development, negative regulation of BMP signaling pathway, negative regulation of canonical Wnt signaling pathway, negative regulation of muscle cell differentiation, negative regulation of muscle organ development, negative regulation of muscle tissue development, negative regulation of ossification, negative regulation of peptidyl-serine phosphorylation, negative regulation of phosphorylation, negative regulation of protein phosphorylation, negative regulation of receptor activity, negative regulation of striated muscle cell differentiation, negative regulation of striated muscle tissue development, negative regulation of transmembrane receptor protein serine/threonine kinase signaling pathway, negative regulation of Wnt signaling pathway, ossification, peptidyl-serine modification, peptidyl-serine phosphorylation, receptor internalization, receptor metabolic process, regulation of BMP signaling pathway, regulation of canonical Wnt signaling pathway, regulation of cardiac muscle tissue development, regulation of embryonic development, regulation of endocytosis, regulation of heart morphogenesis, regulation of muscle organ development, regulation of muscle tissue development, regulation of organ morphogenesis, regulation of ossification, regulation of peptidyl-serine phosphorylation, regulation of protein complex assembly, regulation of skeletal muscle tissue development, regulation of striated muscle cell differentiation, regulation of striated muscle tissue development, regulation of transmembrane receptor protein serine/threonine kinase signaling pathway, regulation of vesicle-mediated transport, regulation of Wnt signaling pathway, segmentation, skeletal muscle tissue development, skin development, somite development, somitogenesis, striated muscle cell differentiation, transmembrane receptor protein serine/threonine kinase signaling pathway, Wnt signaling pathway | http://www.ncbi.nlm.nih.gov/sites/entrez?db=gene&cmd=search&term=13380 |
| Tnfsf10 |  | 0.690762888975919 | apoptotic mitochondrial changes, cytokine receptor binding, extrinsic apoptotic signaling pathway, mitochondrion organization, positive regulation of apoptotic signaling pathway, positive regulation of cysteine-type endopeptidase activity, positive regulation of cysteine-type endopeptidase activity involved in apoptotic process, positive regulation of endopeptidase activity, positive regulation of extrinsic apoptotic signaling pathway, positive regulation of mitochondrion organization, positive regulation of organelle organization, positive regulation of peptidase activity, positive regulation of release of cytochrome c from mitochondria, regulation of cysteine-type endopeptidase activity, regulation of cysteine-type endopeptidase activity involved in apoptotic process, regulation of endopeptidase activity, regulation of extrinsic apoptotic signaling pathway, regulation of mitochondrion organization, regulation of release of cytochrome c from mitochondria, release of cytochrome c from mitochondria, tumor necrosis factor receptor superfamily binding | http://www.ncbi.nlm.nih.gov/sites/entrez?db=gene&cmd=search&term=22035 |
| Mapk8 |  | 0.6831871782330317 | cell projection cytoplasm, cellular protein complex assembly, cellular response to biotic stimulus, cellular response to hydrogen peroxide, cellular response to inorganic substance, cellular response to lipid, cellular response to lipopolysaccharide, cellular response to molecule of bacterial origin, cellular response to nitrogen compound, cellular response to oxidative stress, cellular response to reactive oxygen species, dendrite cytoplasm, determination of bilateral symmetry, DNA replication, histone deacetylase binding, JNK cascade, necrotic cell death, negative regulation of binding, negative regulation of protein binding, ossification, peptidyl-serine modification, peptidyl-serine phosphorylation, peptidyl-threonine modification, peptidyl-threonine phosphorylation, positive regulation of apoptotic signaling pathway, positive regulation of cell migration, positive regulation of cell motility, positive regulation of cellular component movement, positive regulation of DNA metabolic process, positive regulation of DNA replication, positive regulation of microtubule polymerization, positive regulation of microtubule polymerization or depolymerization, positive regulation of organelle organization, positive regulation of protein complex assembly, programmed necrotic cell death, regulation of binding, regulation of DNA metabolic process, regulation of DNA replication, regulation of histone deacetylation, regulation of microtubule polymerization, regulation of protein binding, regulation of protein complex assembly, regulation of protein deacetylation, response to bacterium, response to cadmium ion, response to hydrogen peroxide, response to inorganic substance, response to light stimulus, response to lipopolysaccharide, response to metal ion, response to molecule of bacterial origin, response to oxidative stress, response to radiation, response to reactive oxygen species, response to UV, specification of symmetry, stress-activated MAPK cascade, stress-activated protein kinase signaling cascade | http://www.ncbi.nlm.nih.gov/sites/entrez?db=gene&cmd=search&term=26419 |
| Ctsb |  | 0.6808554474183148 | apical plasma membrane, caveola, cysteine-type endopeptidase activity, cysteine-type peptidase activity, decidualization, endopeptidase activity, external side of plasma membrane, female pregnancy, maternal placenta development, maternal process involved in female pregnancy, membrane raft, placenta development | http://www.ncbi.nlm.nih.gov/sites/entrez?db=gene&cmd=search&term=13030 |
| Pik3cg |  | 0.6706179963795121 | 1-phosphatidylinositol-3-kinase activity, acute inflammatory response, calcium ion homeostasis, cellular calcium ion homeostasis, cellular divalent inorganic cation homeostasis, cytosolic calcium ion homeostasis, fibroblast apoptotic process, glycerophospholipid biosynthetic process, negative regulation of catabolic process, negative regulation of lipid catabolic process, phosphatidylinositol 3-kinase activity, phosphatidylinositol 3-kinase complex, phosphatidylinositol kinase activity, phosphatidylinositol phosphate kinase activity, phosphatidylinositol phosphorylation, phosphatidylinositol-3-phosphate biosynthetic process, positive regulation of acute inflammatory response, positive regulation of cytosolic calcium ion concentration, positive regulation of defense response, positive regulation of inflammatory response, positive regulation of MAP kinase activity, positive regulation of protein kinase B signaling, positive regulation of protein serine/threonine kinase activity, positive regulation of response to external stimulus, protein kinase B signaling, regulation of acute inflammatory response, regulation of fibroblast apoptotic process, regulation of inflammatory response, regulation of lipid metabolic process, regulation of MAP kinase activity, regulation of protein kinase B signaling, vesicle localization | http://www.ncbi.nlm.nih.gov/sites/entrez?db=gene&cmd=search&term=30955 |
| Tnfrsf11a | | 0.6705034911037497 | acid secretion, acute inflammatory response, acute-phase response, adaptive immune response, carboxylic acid transport, cellular response to tumor necrosis factor, circadian rhythm, cytokine binding, cytokine-mediated signaling pathway, ERK1 and ERK2 cascade, external side of plasma membrane, fatty acid derivative transport, fatty acid transport, fever generation, gland development, heat generation, icosanoid secretion, icosanoid transport, JNK cascade, lipid transport, lymph node development, mammary gland alveolus development, mammary gland development, mammary gland lobule development, monocarboxylic acid transport, multicellular organismal homeostasis, myeloid cell differentiation, myeloid leukocyte differentiation, organic acid transport, organic anion transport, ossification, osteoclast differentiation, positive regulation of acute inflammatory response, positive regulation of defense response, positive regulation of ERK1 and ERK2 cascade, positive regulation of fatty acid transport, positive regulation of heat generation, positive regulation of homeostatic process, positive regulation of icosanoid secretion, positive regulation of inflammatory response, positive regulation of ion transport, positive regulation of JUN kinase activity, positive regulation of lipid transport, positive regulation of MAP kinase activity, positive regulation of NF-kappaB transcription factor activity, positive regulation of organic acid transport, positive regulation of prostaglandin secretion, positive regulation of protein serine/threonine kinase activity, positive regulation of response to external stimulus, positive regulation of secretion, positive regulation of sequence-specific DNA binding transcription factor activity, prostaglandin secretion, prostaglandin transport, regulation of acute inflammatory response, regulation of anion transport, regulation of ERK1 and ERK2 cascade, regulation of fatty acid transport, regulation of fever generation, regulation of heat generation, regulation of homeostatic process, regulation of icosanoid secretion, regulation of inflammatory response, regulation of JNK cascade, regulation of JUN kinase activity, regulation of lipid transport, regulation of MAP kinase activity, regulation of organic acid transport, regulation of prostaglandin secretion, regulation of stress-activated MAPK cascade, regulation of stress-activated protein kinase signaling cascade, response to bacterium, response to interleukin-1, response to lipopolysaccharide, response to molecule of bacterial origin, response to tumor necrosis factor, stress-activated MAPK cascade, stress-activated protein kinase signaling cascade, temperature homeostasis, tumor necrosis factor-mediated signaling pathway | http://www.ncbi.nlm.nih.gov/sites/entrez?db=gene&cmd=search&term=21934 |
| Cd44 |  | 0.661119701505929 | adaptive immune response, aminoglycan catabolic process, aminoglycan metabolic process, apical plasma membrane, B cell apoptotic process, basolateral plasma membrane, branching involved in ureteric bud morphogenesis, branching morphogenesis of an epithelial tube, cell-cell adhesion, cytokine binding, DNA damage response, signal transduction by p53 class mediator, epithelial tube morphogenesis, ERK1 and ERK2 cascade, external side of plasma membrane, gland development, gland morphogenesis, glycosaminoglycan catabolic process, glycosaminoglycan metabolic process, heterotypic cell-cell adhesion, homotypic cell-cell adhesion, hyaluronan metabolic process, inflammatory cell apoptotic process, intrinsic apoptotic signaling pathway, intrinsic apoptotic signaling pathway by p53 class mediator, intrinsic apoptotic signaling pathway in response to DNA damage, intrinsic apoptotic signaling pathway in response to DNA damage by p53 class mediator, leukocyte aggregation, leukocyte apoptotic process, leukocyte cell-cell adhesion, lymphocyte apoptotic process, morphogenesis of a branching epithelium, morphogenesis of a branching structure, myeloid cell apoptotic process, negative regulation of apoptotic signaling pathway, negative regulation of B cell apoptotic process, negative regulation of cysteine-type endopeptidase activity, negative regulation of cysteine-type endopeptidase activity involved in apoptotic process, negative regulation of DNA damage response, signal transduction by p53 class mediator, negative regulation of endopeptidase activity, negative regulation of intracellular signal transduction, negative regulation of intrinsic apoptotic signaling pathway, negative regulation of intrinsic apoptotic signaling pathway by p53 class mediator, negative regulation of intrinsic apoptotic signaling pathway in response to DNA damage, negative regulation of intrinsic apoptotic signaling pathway in response to DNA damage by p53 class mediator, negative regulation of leukocyte apoptotic process, negative regulation of lymphocyte apoptotic process, negative regulation of peptidase activity, negative regulation of signal transduction by p53 class mediator, peptidyl-serine modification, peptidyl-serine phosphorylation, peptidyl-tyrosine modification, peptidyl-tyrosine phosphorylation, phosphoprotein binding, positive regulation of adaptive immune response, positive regulation of cell adhesion, positive regulation of cell-cell adhesion, positive regulation of ERK1 and ERK2 cascade, positive regulation of immune response, positive regulation of leukocyte apoptotic process, positive regulation of peptidyl-serine phosphorylation, positive regulation of peptidyl-tyrosine phosphorylation, prostate gland morphogenesis, regulation of adaptive immune response, regulation of B cell apoptotic process, regulation of cell adhesion, regulation of cell-cell adhesion, regulation of cysteine-type endopeptidase activity, regulation of cysteine-type endopeptidase activity involved in apoptotic process, regulation of DNA damage response, signal transduction by p53 class mediator, regulation of endopeptidase activity, regulation of ERK1 and ERK2 cascade, regulation of heterotypic cell-cell adhesion, regulation of homeostatic process, regulation of homotypic cell-cell adhesion, regulation of intrinsic apoptotic signaling pathway, regulation of intrinsic apoptotic signaling pathway by p53 class mediator, regulation of intrinsic apoptotic signaling pathway in response to DNA damage by p53 class mediator, regulation of leukocyte apoptotic process, regulation of lymphocyte apoptotic process, regulation of peptidyl-serine phosphorylation, regulation of peptidyl-tyrosine phosphorylation, renal system development, reproductive structure development, reproductive system development, signal transduction by p53 class mediator, signal transduction in response to DNA damage, urogenital system development, Wnt signaling pathway, wound healing | http://www.ncbi.nlm.nih.gov/sites/entrez?db=gene&cmd=search&term=12505 |
| Itgav |  | 0.6594538431645164 | ameboidal cell migration, anatomical structure homeostasis, apoptotic cell clearance, bone remodeling, bone resorption, calcium ion homeostasis, calcium ion transport, calcium ion transport into cytosol, cell junction assembly, cell junction organization, cell-cell junction organization, cell-matrix adhesion, cell-substrate adhesion, cellular calcium ion homeostasis, cellular divalent inorganic cation homeostasis, cytosolic calcium ion homeostasis, cytosolic calcium ion transport, embryonic placenta development, endothelial cell migration, epithelial cell migration, ERK1 and ERK2 cascade, external side of plasma membrane, extrinsic apoptotic signaling pathway, extrinsic apoptotic signaling pathway in absence of ligand, foam cell differentiation, integrin complex, interaction with host, interspecies interaction between organisms, lipid storage, lipid transport, lipoprotein metabolic process, macrophage derived foam cell differentiation, maintenance of location, negative regulation of apoptotic signaling pathway, negative regulation of extrinsic apoptotic signaling pathway, negative regulation of sequestering of calcium ion, osteoblast proliferation, placenta development, positive regulation of cell adhesion, positive regulation of cell migration, positive regulation of cell motility, positive regulation of cellular component movement, positive regulation of cytosolic calcium ion concentration, protein kinase C binding, receptor biosynthetic process, receptor complex, receptor metabolic process, regulation of bone remodeling, regulation of cell adhesion, regulation of endocytosis, regulation of extrinsic apoptotic signaling pathway, regulation of homeostatic process, regulation of ion homeostasis, regulation of lipid transport, regulation of macrophage derived foam cell differentiation, regulation of multi-organism process, regulation of receptor biosynthetic process, regulation of sequestering of calcium ion, regulation of symbiosis, encompassing mutualism through parasitism, regulation of tissue remodeling, regulation of vesicle-mediated transport, release of sequestered calcium ion into cytosol, reproductive structure development, reproductive system development, response to bacterium, response to inorganic substance, response to molecule of bacterial origin, response to oxidative stress, response to reactive oxygen species, sex differentiation, signal transduction in absence of ligand, symbiosis, encompassing mutualism through parasitism, tissue homeostasis, tissue remodeling | http://www.ncbi.nlm.nih.gov/sites/entrez?db=gene&cmd=search&term=16410 |
| Tgm2 |  | 0.6589293605512521 | apoptotic cell clearance, blood vessel remodeling, branching involved in salivary gland morphogenesis, calcium ion homeostasis, cellular calcium ion homeostasis, cellular divalent inorganic cation homeostasis, cytosolic calcium ion homeostasis, gland development, gland morphogenesis, I-kappaB kinase/NF-kappaB signaling, morphogenesis of a branching epithelium, morphogenesis of a branching structure, muscle cell proliferation, positive regulation of cell adhesion, positive regulation of cytosolic calcium ion concentration, positive regulation of defense response, positive regulation of I-kappaB kinase/NF-kappaB signaling, positive regulation of inflammatory response, positive regulation of response to external stimulus, positive regulation of smooth muscle cell proliferation, protein homooligomerization, proteinaceous extracellular matrix, regulation of cell adhesion, regulation of I-kappaB kinase/NF-kappaB signaling, regulation of inflammatory response, regulation of smooth muscle cell proliferation, salivary gland morphogenesis, smooth muscle cell proliferation, tissue remodeling, tube formation | http://www.ncbi.nlm.nih.gov/sites/entrez?db=gene&cmd=search&term=21817 |
| Sh3glb1 |  | 0.653689344027694 | de novo' posttranslational protein folding, 'de novo' protein folding, apoptotic mitochondrial changes, establishment of protein localization to membrane, establishment of protein localization to mitochondrion, establishment of protein localization to organelle, glycerophospholipid biosynthetic process, mitochondrial membrane organization, mitochondrial outer membrane permeabilization, mitochondrial outer membrane permeabilization involved in programmed cell death, mitochondrion organization, positive regulation of mitochondrial membrane permeability, positive regulation of mitochondrial membrane permeability involved in apoptotic process, positive regulation of mitochondrion organization, positive regulation of organelle organization, positive regulation of protein complex assembly, positive regulation of protein oligomerization, protein insertion into membrane, regulation of mitochondrial membrane permeability, regulation of mitochondrial membrane permeability involved in apoptotic process, regulation of mitochondrion organization, regulation of protein complex assembly, regulation of protein oligomerization | http://www.ncbi.nlm.nih.gov/sites/entrez?db=gene&cmd=search&term=54673 |
| Itgb1 |  | 0.6417589031219946 | acrosomal vesicle, adherens junction, ameboidal cell migration, anatomical structure homeostasis, anchoring junction, anoikis, axon development, blood vessel endothelial cell migration, blood vessel morphogenesis, calcium ion homeostasis, cardiac muscle cell differentiation, cardiac muscle tissue development, cardiocyte differentiation, cell adhesion molecule binding, cell fate commitment, cell fate specification, cell junction assembly, cell junction organization, cell migration involved in sprouting angiogenesis, cell-cell adhesion, cell-cell junction organization, cell-matrix adhesion, cell-substrate adhesion, cellular calcium ion homeostasis, cellular divalent inorganic cation homeostasis, cellular process involved in reproduction in multicellular organism, developmental growth involved in morphogenesis, endothelial cell migration, epithelial cell migration, external side of plasma membrane, fibronectin binding, integrin complex, leukocyte cell-cell adhesion, membrane raft, multicellular organismal homeostasis, muscle cell development, muscle cell differentiation, negative regulation of anoikis, negative regulation of neuron differentiation, organelle assembly, peptidyl-tyrosine modification, peptidyl-tyrosine phosphorylation, positive regulation of cell adhesion, positive regulation of cell migration, positive regulation of cell motility, positive regulation of cell-substrate adhesion, positive regulation of cellular component movement, positive regulation of neuron differentiation, positive regulation of neuron projection development, positive regulation of peptidyl-tyrosine phosphorylation, protease binding, proteinaceous extracellular matrix, receptor complex, regulation of anoikis, regulation of cell adhesion, regulation of cell-substrate adhesion, regulation of endocytosis, regulation of peptidyl-tyrosine phosphorylation, regulation of vesicle-mediated transport, secretory granule, sperm part, striated muscle cell development, striated muscle cell differentiation, tight junction assembly, tissue homeostasis | http://www.ncbi.nlm.nih.gov/sites/entrez?db=gene&cmd=search&term=16412 |
| Bcl2l11 |  | 0.6400232369845911 | activation of cysteine-type endopeptidase activity, activation of cysteine-type endopeptidase activity involved in apoptotic process, apoptotic mitochondrial changes, apoptotic process involved in development, apoptotic process involved in morphogenesis, appendage development, appendage morphogenesis, B cell apoptotic process, B cell homeostasis, cell killing, cell-matrix adhesion, cell-substrate adhesion, developmental programmed cell death, disruption of cells of other organism, embryonic appendage morphogenesis, embryonic digit morphogenesis, embryonic limb morphogenesis, extrinsic apoptotic signaling pathway, extrinsic apoptotic signaling pathway in absence of ligand, fibroblast apoptotic process, gland development, homeostasis of number of cells, interaction with host, interspecies interaction between organisms, intrinsic apoptotic signaling pathway, intrinsic apoptotic signaling pathway in response to DNA damage, kidney development, killing of cells of other organism, leukocyte apoptotic process, leukocyte homeostasis, limb development, limb morphogenesis, lymphocyte apoptotic process, lymphocyte homeostasis, male gamete generation, mammary gland development, mitochondrial membrane organization, mitochondrial outer membrane permeabilization involved in programmed cell death, mitochondrial transport, mitochondrion organization, modification by symbiont of host morphology or physiology, modification of morphology or physiology of other organism, modification of morphology or physiology of other organism involved in symbiotic interaction, modulation by virus of host morphology or physiology, multi-organism cellular process, myeloid cell homeostasis, neuron apoptotic process, neuron death, odontogenesis, odontogenesis of dentin-containing tooth, pigmentation, positive regulation of apoptotic signaling pathway, positive regulation of cell cycle, positive regulation of cell killing, positive regulation of cysteine-type endopeptidase activity, positive regulation of cysteine-type endopeptidase activity involved in apoptotic process, positive regulation of endopeptidase activity, positive regulation of intrinsic apoptotic signaling pathway, positive regulation of mitochondrial membrane permeability, positive regulation of mitochondrial membrane permeability involved in apoptotic process, positive regulation of mitochondrion organization, positive regulation of multi-organism process, positive regulation of neuron apoptotic process, positive regulation of neuron death, positive regulation of organelle organization, positive regulation of peptidase activity, positive regulation of protein complex assembly, positive regulation of protein oligomerization, positive regulation of release of cytochrome c from mitochondria, post-embryonic development, post-embryonic morphogenesis, post-embryonic organ development, protein homooligomerization, regulation of cell killing, regulation of cysteine-type endopeptidase activity, regulation of cysteine-type endopeptidase activity involved in apoptotic process, regulation of developmental pigmentation, regulation of endopeptidase activity, regulation of fibroblast apoptotic process, regulation of intrinsic apoptotic signaling pathway, regulation of mitochondrial membrane permeability, regulation of mitochondrial membrane permeability involved in apoptotic process, regulation of mitochondrion organization, regulation of multi-organism process, regulation of neuron apoptotic process, regulation of neuron death, regulation of organ growth, regulation of protein complex assembly, regulation of protein homooligomerization, regulation of protein oligomerization, regulation of release of cytochrome c from mitochondria, regulation of symbiosis, encompassing mutualism through parasitism, release of cytochrome c from mitochondria, renal system development, reproductive structure development, reproductive system development, response to endoplasmic reticulum stress, response to virus, sex differentiation, signal transduction in absence of ligand, spermatogenesis, spleen development, symbiosis, encompassing mutualism through parasitism, T cell apoptotic process, T cell homeostasis, thymocyte apoptotic process, tube formation, urogenital system development, viral process, zymogen activation | http://www.ncbi.nlm.nih.gov/sites/entrez?db=gene&cmd=search&term=12125 |
| Mmp13 |  | 0.6310302640935193 | biomineral tissue development, bone mineralization, cartilage development, collagen metabolic process, connective tissue development, endopeptidase activity, fibronectin binding, lipoprotein particle receptor binding, low-density lipoprotein particle receptor binding, multicellular organismal macromolecule metabolic process, multicellular organismal metabolic process, ossification, proteinaceous extracellular matrix, receptor internalization, receptor metabolic process | http://www.ncbi.nlm.nih.gov/sites/entrez?db=gene&cmd=search&term=17386 |
| Creb1 |  | 0.6271718241905515 | activating transcription factor binding, axon development, body fluid secretion, cellular response to inorganic substance, cellular response to metal ion, cellular response to transforming growth factor beta stimulus, chromatin, circadian rhythm, cognition, double-stranded DNA binding, euchromatin, fat cell differentiation, forebrain development, gland development, histone acetyltransferase binding, hormone secretion, hormone transport, lactation, learning or memory, mammary gland development, memory, myeloid cell differentiation, myeloid leukocyte differentiation, nuclear chromatin, nuclear chromosome part, nuclear euchromatin, osteoclast differentiation, positive regulation of cytokine production, positive regulation of fat cell differentiation, positive regulation of leukocyte differentiation, positive regulation of lipid metabolic process, positive regulation of myeloid cell differentiation, positive regulation of myeloid leukocyte differentiation, positive regulation of osteoclast differentiation, positive regulation of secretion, positive regulation of transforming growth factor beta production, regulation of body fluid levels, regulation of fat cell differentiation, regulation of hormone secretion, regulation of leukocyte differentiation, regulation of lipid biosynthetic process, regulation of lipid metabolic process, regulation of myeloid cell differentiation, regulation of myeloid leukocyte differentiation, regulation of osteoclast differentiation, regulation of protein complex assembly, regulation of transforming growth factor beta production, response to drug, response to inorganic substance, response to metal ion, response to transforming growth factor beta, RNA polymerase II activating transcription factor binding, RNA polymerase II distal enhancer sequence-specific DNA binding, RNA polymerase II distal enhancer sequence-specific DNA binding transcription factor activity, RNA polymerase II regulatory region DNA binding, RNA polymerase II regulatory region sequence-specific DNA binding, RNA polymerase II transcription factor binding, secretion by tissue, sequence-specific DNA binding RNA polymerase II transcription factor activity, structure-specific DNA binding, transcription regulatory region sequence-specific DNA binding, transforming growth factor beta production, transforming growth factor beta receptor signaling pathway, transmembrane receptor protein serine/threonine kinase signaling pathway | http://www.ncbi.nlm.nih.gov/sites/entrez?db=gene&cmd=search&term=12912 |
| Htt |  | 0.6220864104986487 | acidic amino acid transport, aging, amide transport, amino acid transport, anterior/posterior pattern specification, autophagic vacuole, carboxylic acid transport, cell aging, cell projection cytoplasm, cognition, cytoskeleton-dependent intracellular transport, dicarboxylic acid transport, dopamine receptor signaling pathway, establishment of protein localization to organelle, extrinsic apoptotic signaling pathway, forebrain development, Golgi organization, hormone secretion, hormone transport, insulin secretion, iron ion homeostasis, L-glutamate transport, late endosome, learning, learning or memory, male gamete generation, microtubule-based transport, mitochondrial membrane organization, mitochondrial transport, mitochondrion organization, morphogenesis of embryonic epithelium, negative regulation of apoptotic signaling pathway, negative regulation of cysteine-type endopeptidase activity, negative regulation of endopeptidase activity, negative regulation of extrinsic apoptotic signaling pathway, negative regulation of neuron apoptotic process, negative regulation of neuron death, negative regulation of peptidase activity, neuron apoptotic process, neuron death, nuclear import, organelle transport along microtubule, organic acid transport, organic anion transport, organic hydroxy compound biosynthetic process, peptide hormone secretion, peptide secretion, peptide transport, protein import, protein import into nucleus, protein localization to nucleus, protein targeting, protein targeting to nucleus, regulation of cysteine-type endopeptidase activity, regulation of endopeptidase activity, regulation of extrinsic apoptotic signaling pathway, regulation of intracellular protein transport, regulation of mitochondrial membrane permeability, regulation of mitochondrial membrane potential, regulation of neuron apoptotic process, regulation of neuron death, regulation of nucleocytoplasmic transport, regulation of synaptic transmission, response to calcium ion, response to inorganic substance, response to light stimulus, response to metal ion, response to radiation, ribonucleoprotein granule, single-organism cellular localization, single-organism localization, single-organism nuclear import, spermatogenesis, telencephalon development, transition metal ion homeostasis, vesicle localization, vesicle membrane, vesicle transport along microtubule | http://www.ncbi.nlm.nih.gov/sites/entrez?db=gene&cmd=search&term=15194 |
| Csf1 |  | 0.6211963162702205 | anatomical structure homeostasis, branching involved in mammary gland duct morphogenesis, branching morphogenesis of an epithelial tube, cell-matrix adhesion, cell-substrate adhesion, connective tissue development, cytokine activity, cytokine receptor binding, developmental growth involved in morphogenesis, epithelial tube morphogenesis, foam cell differentiation, gland development, gland morphogenesis, homeostasis of number of cells, homeostasis of number of cells within a tissue, leukocyte proliferation, macrophage derived foam cell differentiation, macrophage differentiation, mammary gland development, mammary gland duct morphogenesis, mammary gland epithelium development, mammary gland morphogenesis, monocyte differentiation, mononuclear cell proliferation, morphogenesis of a branching epithelium, morphogenesis of a branching structure, multicellular organismal homeostasis, myeloid cell differentiation, myeloid leukocyte differentiation, odontogenesis, odontogenesis of dentin-containing tooth, ossification, osteoclast differentiation, positive regulation of cell adhesion, positive regulation of cell migration, positive regulation of cell motility, positive regulation of cell-substrate adhesion, positive regulation of cellular component movement, positive regulation of leukocyte differentiation, positive regulation of leukocyte proliferation, positive regulation of macrophage differentiation, positive regulation of mononuclear cell proliferation, positive regulation of myeloid cell differentiation, positive regulation of myeloid leukocyte differentiation, positive regulation of osteoclast differentiation, positive regulation of Ras protein signal transduction, receptor complex, regulation of cell adhesion, regulation of cell-matrix adhesion, regulation of cell-substrate adhesion, regulation of leukocyte differentiation, regulation of leukocyte proliferation, regulation of macrophage derived foam cell differentiation, regulation of macrophage differentiation, regulation of mononuclear cell proliferation, regulation of myeloid cell differentiation, regulation of myeloid leukocyte differentiation, regulation of odontogenesis, regulation of organ morphogenesis, regulation of ossification, regulation of osteoclast differentiation, tissue homeostasis | http://www.ncbi.nlm.nih.gov/sites/entrez?db=gene&cmd=search&term=12977 |
| Traf6 |  | 0.6187120784304584 | activation of immune response, activation of protein kinase activity, adaptive immune response, adaptive immune response based on somatic recombination of immune receptors built from immunoglobulin superfamily domains, anatomical structure homeostasis, antigen processing and presentation of exogenous antigen, antigen processing and presentation of exogenous peptide antigen, antigen processing and presentation of exogenous peptide antigen via MHC class II, antigen processing and presentation of peptide antigen via MHC class II, antigen processing and presentation of peptide or polysaccharide antigen via MHC class II, antigen receptor-mediated signaling pathway, bone remodeling, bone resorption, CD40 receptor complex, cellular response to biotic stimulus, cellular response to lipid, cellular response to lipopolysaccharide, cellular response to molecule of bacterial origin, cytokine biosynthetic process, cytokine metabolic process, cytokine production involved in immune response, cytokine-mediated signaling pathway, dendritic cell differentiation, embryonic epithelial tube formation, epithelial tube formation, epithelial tube morphogenesis, histone deacetylase binding, I-kappaB kinase/NF-kappaB signaling, immune response-activating signal transduction, immunoglobulin production, immunoglobulin secretion, interleukin-12 biosynthetic process, interleukin-12 production, interleukin-6 biosynthetic process, interleukin-6 production, JNK cascade, leukocyte mediated immunity, leukocyte proliferation, lipid particle, lipopolysaccharide-mediated signaling pathway, lymphocyte mediated immunity, lymphocyte proliferation, mitogen-activated protein kinase kinase kinase binding, mononuclear cell proliferation, morphogenesis of embryonic epithelium, multicellular organismal homeostasis, muscle cell proliferation, myeloid cell differentiation, myeloid dendritic cell activation, myeloid dendritic cell differentiation, myeloid leukocyte activation, myeloid leukocyte differentiation, neural tube closure, neural tube development, neural tube formation, odontogenesis, odontogenesis of dentin-containing tooth, ossification, osteoclast differentiation, positive regulation of adaptive immune response, positive regulation of binding, positive regulation of cell activation, positive regulation of cytokine biosynthetic process, positive regulation of cytokine production, positive regulation of cytokine production involved in immune response, positive regulation of I-kappaB kinase/NF-kappaB signaling, positive regulation of immune effector process, positive regulation of immune response, positive regulation of JUN kinase activity, positive regulation of leukocyte activation, positive regulation of leukocyte differentiation, positive regulation of leukocyte proliferation, positive regulation of lymphocyte activation, positive regulation of lymphocyte proliferation, positive regulation of MAP kinase activity, positive regulation of mononuclear cell proliferation, positive regulation of myeloid cell differentiation, positive regulation of myeloid leukocyte differentiation, positive regulation of NF-kappaB transcription factor activity, positive regulation of osteoclast differentiation, positive regulation of production of molecular mediator of immune response, positive regulation of protein serine/threonine kinase activity, positive regulation of response to external stimulus, positive regulation of sequence-specific DNA binding transcription factor activity, positive regulation of smooth muscle cell proliferation, positive regulation of T cell activation, positive regulation of T cell proliferation, primary neural tube formation, production of molecular mediator of immune response, protein N-terminus binding, protein secretion, receptor complex, regulation of adaptive immune response, regulation of binding, regulation of cytokine biosynthetic process, regulation of cytokine production involved in immune response, regulation of DNA binding, regulation of I-kappaB kinase/NF-kappaB signaling, regulation of immune effector process, regulation of immunoglobulin production, regulation of immunoglobulin secretion, regulation of interleukin-12 biosynthetic process, regulation of interleukin-12 production, regulation of interleukin-6 biosynthetic process, regulation of interleukin-6 production, regulation of JNK cascade, regulation of JUN kinase activity, regulation of leukocyte differentiation, regulation of leukocyte mediated immunity, regulation of leukocyte proliferation, regulation of lymphocyte activation, regulation of lymphocyte proliferation, regulation of MAP kinase activity, regulation of mononuclear cell proliferation, regulation of multi-organism process, regulation of myeloid cell differentiation, regulation of myeloid leukocyte differentiation, regulation of osteoclast differentiation, regulation of production of molecular mediator of immune response, regulation of protein secretion, regulation of smooth muscle cell proliferation, regulation of stress-activated MAPK cascade, regulation of stress-activated protein kinase signaling cascade, regulation of T cell activation, regulation of T cell proliferation, regulation of transcription regulatory region DNA binding, response to bacterium, response to interleukin-1, response to lipopolysaccharide, response to molecule of bacterial origin, small conjugating protein ligase binding, smooth muscle cell proliferation, stress-activated MAPK cascade, stress-activated protein kinase signaling cascade, T cell proliferation, thioesterase binding, tissue homeostasis, tissue remodeling, tube closure, tube formation, ubiquitin protein ligase binding | http://www.ncbi.nlm.nih.gov/sites/entrez?db=gene&cmd=search&term=22034 |
| Psen1 |  | 0.6165461786009563 | acidic amino acid transport, activation of immune response, activation of protein kinase activity, amino acid transport, anagen, anatomical structure maturation, anterior/posterior pattern specification, antigen receptor-mediated signaling pathway, apical plasma membrane, appendage development, appendage morphogenesis, autophagic vacuole assembly, autophagy, beta-catenin binding, cadherin binding, calcium ion homeostasis, calcium ion transport, carboxylic acid transport, cell activation involved in immune response, cell adhesion molecule binding, cell cortex, cell fate commitment, cell fate specification, cell-cell adhesion, cellular calcium ion homeostasis, cellular divalent inorganic cation homeostasis, cellular response to external stimulus, cellular response to extracellular stimulus, cellular response to nutrient levels, cellular response to starvation, cerebral cortex development, coagulation, cognition, determination of bilateral symmetry, determination of heart left/right asymmetry, determination of left/right symmetry, developmental maturation, dicarboxylic acid transport, embryonic appendage morphogenesis, embryonic heart tube development, embryonic heart tube morphogenesis, embryonic limb morphogenesis, embryonic organ morphogenesis, endopeptidase activity, endoplasmic reticulum calcium ion homeostasis, epidermis development, epithelial cell proliferation, epithelial tube morphogenesis, forebrain development, hair cycle, hair cycle process, hair follicle development, hair follicle maturation, heart looping, heart morphogenesis, hematopoietic progenitor cell differentiation, immune response-activating signal transduction, L-glutamate transport, learning or memory, leukocyte activation involved in immune response, limb development, limb morphogenesis, lymphocyte activation involved in immune response, macroautophagy, membrane protein ectodomain proteolysis, membrane protein proteolysis, membrane raft, memory, mitochondrial transport, molting cycle, molting cycle process, myeloid cell differentiation, myeloid leukocyte differentiation, negative regulation of apoptotic signaling pathway, negative regulation of cell development, negative regulation of kinase activity, negative regulation of neurogenesis, negative regulation of neuron apoptotic process, negative regulation of neuron death, negative regulation of phosphorylation, negative regulation of protein kinase activity, negative regulation of protein phosphorylation, negative regulation of receptor activity, negative regulation of transferase activity, neural tube development, neuron apoptotic process, neuron death, neuron migration, organelle assembly, organelle outer membrane, organic acid transport, organic anion transport, organic hydroxy compound transport, outer membrane, pallium development, peptidyl-tyrosine modification, peptidyl-tyrosine phosphorylation, positive regulation of catabolic process, positive regulation of cellular catabolic process, positive regulation of coagulation, positive regulation of immune response, positive regulation of MAP kinase activity, positive regulation of protein catabolic process, positive regulation of protein processing, positive regulation of protein serine/threonine kinase activity, positive regulation of proteolysis, post-embryonic development, receptor metabolic process, regulation of binding, regulation of cell morphogenesis involved in differentiation, regulation of coagulation, regulation of MAP kinase activity, regulation of neuron apoptotic process, regulation of neuron death, regulation of peptidyl-tyrosine phosphorylation, regulation of protein binding, regulation of protein catabolic process, regulation of protein ubiquitination, regulation of synaptic transmission, response to extracellular stimulus, response to nutrient levels, response to oxidative stress, response to starvation, segmentation, skin development, smooth endoplasmic reticulum, somite development, somitogenesis, specification of symmetry, T cell activation involved in immune response, telencephalon development, vacuole organization, vesicle localization | http://www.ncbi.nlm.nih.gov/sites/entrez?db=gene&cmd=search&term=19164 |
| Plau |  | 0.6159888119021589 | blood coagulation, blood vessel morphogenesis, cell-matrix adhesion, cell-substrate adhesion, coagulation, epithelial cell proliferation, hemostasis, muscle cell migration, neuron death, ovulation, positive regulation of cell migration, positive regulation of cell motility, positive regulation of cellular component movement, positive regulation of reactive oxygen species metabolic process, reactive oxygen species metabolic process, regeneration, regulation of blood coagulation, regulation of body fluid levels, regulation of cell adhesion, regulation of cell-matrix adhesion, regulation of cell-substrate adhesion, regulation of coagulation, regulation of epithelial cell proliferation, regulation of hemostasis, regulation of organ morphogenesis, regulation of reactive oxygen species metabolic process, regulation of wound healing, response to decreased oxygen levels, response to hypoxia, response to oxygen levels, skeletal muscle tissue regeneration, tissue regeneration, wound healing | http://www.ncbi.nlm.nih.gov/sites/entrez?db=gene&cmd=search&term=18792 |
| Notch1 |  | 0.6156280286755177 | acrosomal vesicle, ameboidal cell migration, anagen, anatomical structure maturation, anoikis, anterior/posterior pattern specification, apoptotic process involved in development, apoptotic process involved in morphogenesis, appendage development, appendage morphogenesis, astrocyte differentiation, axon development, blood vessel endothelial cell migration, blood vessel morphogenesis, BMP signaling pathway, branching morphogenesis of an epithelial tube, canonical Wnt signaling pathway, cardiac muscle cell differentiation, cardiac muscle tissue development, cardiocyte differentiation, cell chemotaxis, cell fate commitment, cell fate specification, cell migration involved in sprouting angiogenesis, cell surface receptor signaling pathway involved in heart development, cell-substrate adhesion, cellular response to hormone stimulus, cellular response to oxygen levels, core promoter binding, cytokine secretion, determination of bilateral symmetry, determination of heart left/right asymmetry, determination of left/right symmetry, developmental growth involved in morphogenesis, developmental maturation, developmental programmed cell death, embryonic appendage morphogenesis, embryonic digit morphogenesis, embryonic heart tube development, embryonic heart tube morphogenesis, embryonic limb morphogenesis, embryonic organ morphogenesis, endoderm development, endothelial cell chemotaxis, endothelial cell migration, epidermal cell differentiation, epidermis development, epithelial cell migration, epithelial cell proliferation, epithelial to mesenchymal transition, epithelial tube morphogenesis, extracellular matrix assembly, forebrain development, gland development, gland morphogenesis, glial cell differentiation, gliogenesis, hair cycle, hair cycle process, hair follicle development, hair follicle maturation, heart looping, heart morphogenesis, hematopoietic progenitor cell differentiation, hepaticobiliary system development, JAK-STAT cascade, keratinocyte differentiation, limb development, limb morphogenesis, liver development, male gamete generation, mesenchymal cell development, mesenchymal cell differentiation, mesenchyme development, molting cycle, molting cycle process, morphogenesis of a branching epithelium, morphogenesis of a branching structure, muscle cell development, muscle cell differentiation, muscle cell proliferation, muscle organ development, myotube differentiation, negative regulation of anoikis, negative regulation of blood vessel endothelial cell migration, negative regulation of BMP signaling pathway, negative regulation of canonical Wnt signaling pathway, negative regulation of cell adhesion, negative regulation of cell development, negative regulation of exocytosis, negative regulation of muscle cell differentiation, negative regulation of myoblast differentiation, negative regulation of neurogenesis, negative regulation of neuron differentiation, negative regulation of ossification, negative regulation of secretion, negative regulation of striated muscle cell differentiation, negative regulation of transmembrane receptor protein serine/threonine kinase signaling pathway, negative regulation of Wnt signaling pathway, neural precursor cell proliferation, neural tube development, neuroblast proliferation, ossification, osteoblast differentiation, positive regulation of astrocyte differentiation, positive regulation of cell development, positive regulation of cell migration, positive regulation of cell motility, positive regulation of cellular component movement, positive regulation of epithelial cell proliferation, positive regulation of epithelial to mesenchymal transition, positive regulation of glial cell differentiation, positive regulation of gliogenesis, positive regulation of JAK-STAT cascade, positive regulation of neuroblast proliferation, positive regulation of neurogenesis, positive regulation of stem cell proliferation, prostate gland morphogenesis, protein secretion, receptor complex, regulation of angiogenesis, regulation of anoikis, regulation of astrocyte differentiation, regulation of behavior, regulation of blood vessel endothelial cell migration, regulation of BMP signaling pathway, regulation of canonical Wnt signaling pathway, regulation of cardiac muscle cell proliferation, regulation of cardiac muscle tissue development, regulation of cell adhesion, regulation of cell migration involved in sprouting angiogenesis, regulation of cell morphogenesis involved in differentiation, regulation of cell-substrate adhesion, regulation of chemotaxis, regulation of DNA-templated transcription in response to stress, regulation of embryonic development, regulation of endothelial cell chemotaxis, regulation of endothelial cell migration, regulation of epithelial cell differentiation, regulation of epithelial cell migration, regulation of epithelial cell proliferation, regulation of glial cell differentiation, regulation of gliogenesis, regulation of heart morphogenesis, regulation of JAK-STAT cascade, regulation of muscle organ development, regulation of muscle tissue development, regulation of neural precursor cell proliferation, regulation of neuroblast proliferation, regulation of organ growth, regulation of organ morphogenesis, regulation of ossification, regulation of skeletal muscle tissue development, regulation of stem cell proliferation, regulation of striated muscle cell differentiation, regulation of striated muscle tissue development, regulation of transcription from RNA polymerase II promoter in response to stress, regulation of transmembrane receptor protein serine/threonine kinase signaling pathway, regulation of vasculature development, regulation of vesicle-mediated transport, regulation of Wnt signaling pathway, reproductive structure development, reproductive system development, response to decreased oxygen levels, response to hypoxia, response to muramyl dipeptide, response to oxygen levels, response to peptide, secretory granule, segmentation, skeletal muscle cell differentiation, skeletal muscle organ development, skeletal muscle tissue development, skin development, somitogenesis, specification of symmetry, sperm part, spermatogenesis, stem cell development, stem cell differentiation, stem cell proliferation, striated muscle cell development, striated muscle cell differentiation, structure-specific DNA binding, transmembrane receptor protein serine/threonine kinase signaling pathway, tube formation, urogenital system development, vasculogenesis, Wnt signaling pathway | http://www.ncbi.nlm.nih.gov/sites/entrez?db=gene&cmd=search&term=18128 |
| Ptgs2 |  | 0.6113174254217956 | acute inflammatory response, acute-phase response, ameboidal cell migration, anagen, anatomical structure maturation, biomineral tissue development, blood circulation, blood vessel endothelial cell migration, blood vessel morphogenesis, bone mineralization, calcium ion transport, caveola, cell migration involved in sprouting angiogenesis, cognition, decidualization, developmental maturation, endothelial cell migration, epidermal cell differentiation, epidermis development, epithelial cell migration, establishment of protein localization to organelle, fat cell differentiation, female pregnancy, fever generation, hair cycle, hair cycle process, hair follicle development, hair follicle maturation, heat generation, heme binding, I-kappaB kinase/NF-kappaB signaling, keratinocyte differentiation, learning, learning or memory, maternal placenta development, maternal process involved in female pregnancy, membrane raft, memory, molting cycle, molting cycle process, multicellular organismal homeostasis, muscle cell proliferation, negative regulation of calcium ion transport, negative regulation of cell cycle, negative regulation of ion transport, NF-kappaB import into nucleus, nitric oxide biosynthetic process, nitric oxide metabolic process, nuclear import, ossification, ovulation, oxidoreductase activity, acting on paired donors, with incorporation or reduction of molecular oxygen, placenta development, positive regulation of acute inflammatory response, positive regulation of blood vessel endothelial cell migration, positive regulation of cell migration, positive regulation of cell motility, positive regulation of cellular component movement, positive regulation of cytokine production, positive regulation of defense response, positive regulation of endothelial cell migration, positive regulation of epithelial cell migration, positive regulation of fat cell differentiation, positive regulation of heat generation, positive regulation of homeostatic process, positive regulation of inflammatory response, positive regulation of intracellular protein transport, positive regulation of intracellular transport, positive regulation of NF-kappaB import into nucleus, positive regulation of nitric oxide biosynthetic process, positive regulation of nucleocytoplasmic transport, positive regulation of protein import into nucleus, positive regulation of protein transport, positive regulation of response to external stimulus, positive regulation of smooth muscle cell proliferation, positive regulation of transcription factor import into nucleus, positive regulation of transforming growth factor beta production, positive regulation of vasoconstriction, positive regulation vascular endothelial growth factor production, prostaglandin biosynthetic process, prostaglandin metabolic process, prostanoid biosynthetic process, prostanoid metabolic process, protein import, protein import into nucleus, protein localization to nucleus, regulation of acute inflammatory response, regulation of angiogenesis, regulation of blood pressure, regulation of blood vessel endothelial cell migration, regulation of blood vessel size, regulation of calcium ion transport, regulation of cell migration involved in sprouting angiogenesis, regulation of endothelial cell migration, regulation of epithelial cell migration, regulation of fat cell differentiation, regulation of fever generation, regulation of heat generation, regulation of homeostatic process, regulation of I-kappaB kinase/NF-kappaB signaling, regulation of inflammatory response, regulation of intracellular protein transport, regulation of metal ion transport, regulation of NF-kappaB import into nucleus, regulation of nitric oxide biosynthetic process, regulation of nucleocytoplasmic transport, regulation of protein import into nucleus, regulation of protein localization to nucleus, regulation of smooth muscle cell proliferation, regulation of smooth muscle contraction, regulation of synaptic transmission, regulation of transcription factor import into nucleus, regulation of transforming growth factor beta production, regulation of tube size, regulation of vascular endothelial growth factor production, regulation of vasculature development, regulation of vasoconstriction, response to bacterium, response to lipopolysaccharide, response to molecule of bacterial origin, single-organism nuclear import, skin development, smooth muscle cell proliferation, smooth muscle contraction, temperature homeostasis, tetrapyrrole binding, transcription factor import into nucleus, transforming growth factor beta production, vascular endothelial growth factor production, vascular process in circulatory system, vasoconstriction | http://www.ncbi.nlm.nih.gov/sites/entrez?db=gene&cmd=search&term=19225 |
| Gja1 |  | 0.6100148172998516 | adherens junction, anchoring junction, apical plasma membrane, appendage development, appendage morphogenesis, biomineral tissue development, blood circulation, blood vessel morphogenesis, blood vessel remodeling, bone mineralization, bone remodeling, calcium ion homeostasis, calcium ion transport, cardiac muscle tissue development, cell junction assembly, cell junction organization, cell maturation, cell-cell junction organization, cellular calcium ion homeostasis, cellular divalent inorganic cation homeostasis, cytosolic calcium ion homeostasis, determination of bilateral symmetry, determination of heart left/right asymmetry, determination of left/right symmetry, developmental maturation, embryonic appendage morphogenesis, embryonic digit morphogenesis, embryonic heart tube development, embryonic heart tube morphogenesis, embryonic limb morphogenesis, embryonic organ morphogenesis, epithelial cell maturation, epithelial to mesenchymal transition, epithelial tube morphogenesis, heart contraction, heart looping, heart morphogenesis, heart process, I-kappaB kinase/NF-kappaB signaling, late endosome, limb development, limb morphogenesis, membrane depolarization, membrane raft, mesenchymal cell development, mesenchymal cell differentiation, mesenchyme development, mitochondrial outer membrane, multicellular organismal signaling, multivesicular body, muscle cell proliferation, muscle organ development, neuron migration, organelle outer membrane, organic anion transport, ossification, osteoblast differentiation, outer membrane, positive regulation of behavior, positive regulation of catabolic process, positive regulation of cytosolic calcium ion concentration, positive regulation of defense response, positive regulation of I-kappaB kinase/NF-kappaB signaling, positive regulation of protein catabolic process, positive regulation of vasoconstriction, positive regulation of vasodilation, regeneration, regulation of behavior, regulation of blood pressure, regulation of blood vessel size, regulation of body fluid levels, regulation of bone remodeling, regulation of calcium ion transport, regulation of cardiac muscle cell proliferation, regulation of cardiac muscle tissue development, regulation of heart contraction, regulation of homeostatic process, regulation of I-kappaB kinase/NF-kappaB signaling, regulation of ion homeostasis, regulation of membrane depolarization, regulation of metal ion transport, regulation of muscle organ development, regulation of muscle tissue development, regulation of organ growth, regulation of ossification, regulation of protein catabolic process, regulation of striated muscle tissue development, regulation of tissue remodeling, regulation of tube size, regulation of vasoconstriction, regulation of vasodilation, skeletal muscle tissue regeneration, specification of symmetry, stem cell development, stem cell differentiation, tight junction assembly, tissue regeneration, tissue remodeling, vascular process in circulatory system, vasoconstriction, vasodilation, wound healing | http://www.ncbi.nlm.nih.gov/sites/entrez?db=gene&cmd=search&term=14609 |
| Atf4 |  | 0.6057430432809993 | carbohydrate biosynthetic process, dendrite membrane, glucose metabolic process, hexose metabolic process, intrinsic apoptotic signaling pathway, monosaccharide metabolic process, negative regulation of ion transport, neuron apoptotic process, neuron death, positive regulation of neuron apoptotic process, positive regulation of neuron death, protein C-terminus binding, regulation of metal ion transport, regulation of neuron apoptotic process, regulation of neuron death, response to endoplasmic reticulum stress | http://www.ncbi.nlm.nih.gov/sites/entrez?db=gene&cmd=search&term=11911 |
| Eif2s1 |  | 0.6051250852096715 | ribonucleoprotein granule, ribosome binding | http://www.ncbi.nlm.nih.gov/sites/entrez?db=gene&cmd=search&term=13665 |
| Smad5 |  | 0.604791526550554 | blood circulation, BMP signaling pathway, cartilage development, cell fate commitment, cellular process involved in reproduction in multicellular organism, cellular response to organic cyclic compound, cellular response to transforming growth factor beta stimulus, connective tissue development, core promoter binding, heart contraction, heart process, homeostasis of number of cells, myeloid cell differentiation, myeloid cell homeostasis, ossification, osteoblast differentiation, regulation of ossification, response to transforming growth factor beta, RNA polymerase II regulatory region DNA binding, RNA polymerase II regulatory region sequence-specific DNA binding, sex differentiation, small conjugating protein ligase binding, transcription regulatory region sequence-specific DNA binding, transforming growth factor beta receptor signaling pathway, transmembrane receptor protein serine/threonine kinase signaling pathway, ubiquitin protein ligase binding | http://www.ncbi.nlm.nih.gov/sites/entrez?db=gene&cmd=search&term=17129 |
| Fos |  | 0.6036592551361541 | cellular response to calcium ion, cellular response to external stimulus, cellular response to extracellular stimulus, cellular response to inorganic substance, cellular response to metal ion, cellular response to oxidative stress, cellular response to reactive oxygen species, cellular response to transforming growth factor beta stimulus, double-stranded DNA binding, muscle organ development, myeloid cell differentiation, myeloid leukocyte differentiation, osteoclast differentiation, positive regulation of leukocyte differentiation, positive regulation of myeloid cell differentiation, positive regulation of myeloid leukocyte differentiation, positive regulation of osteoclast differentiation, R-SMAD binding, regulation of leukocyte differentiation, regulation of myeloid cell differentiation, regulation of myeloid leukocyte differentiation, regulation of osteoclast differentiation, response to calcium ion, response to drug, response to extracellular stimulus, response to inorganic substance, response to metal ion, response to oxidative stress, response to reactive oxygen species, response to transforming growth factor beta, skeletal muscle cell differentiation, skeletal muscle organ development, skeletal muscle tissue development, SMAD binding, SMAD protein signal transduction, structure-specific DNA binding, transforming growth factor beta receptor signaling pathway, transmembrane receptor protein serine/threonine kinase signaling pathway | http://www.ncbi.nlm.nih.gov/sites/entrez?db=gene&cmd=search&term=14281 |
| Met |  | 0.602878671645037 | activation of MAPK activity, activation of protein kinase activity, ameboidal cell migration, basolateral plasma membrane, beta-catenin binding, branching involved in salivary gland morphogenesis, branching morphogenesis of an epithelial tube, cell chemotaxis, cellular response to hydrogen peroxide, cellular response to oxidative stress, cellular response to reactive oxygen species, DNA replication, endothelial cell chemotaxis, endothelial cell migration, epithelial cell migration, epithelial tube morphogenesis, glucose transport, hepaticobiliary system development, hexose transport, liver development, membrane depolarization, mitosis, morphogenesis of a branching epithelium, morphogenesis of a branching structure, muscle cell differentiation, muscle cell migration, muscle organ development, myotube differentiation, negative regulation of cellular response to oxidative stress, negative regulation of phosphorylation, negative regulation of protein phosphorylation, neuron migration, nuclear division, peptidyl-serine modification, peptidyl-serine phosphorylation, peptidyl-threonine modification, peptidyl-threonine phosphorylation, peptidyl-tyrosine modification, peptidyl-tyrosine phosphorylation, placenta development, positive regulation of behavior, positive regulation of cell cycle, positive regulation of cell cycle process, positive regulation of cell development, positive regulation of cell migration, positive regulation of cell motility, positive regulation of cellular component movement, positive regulation of chemotaxis, positive regulation of DNA metabolic process, positive regulation of DNA replication, positive regulation of endothelial cell migration, positive regulation of epithelial cell migration, positive regulation of MAP kinase activity, positive regulation of neurogenesis, positive regulation of neuron projection development, positive regulation of organelle organization, positive regulation of peptidyl-serine phosphorylation, positive regulation of peptidyl-threonine phosphorylation, positive regulation of protein serine/threonine kinase activity, positive regulation of response to external stimulus, regulation of behavior, regulation of cell division, regulation of cell morphogenesis involved in differentiation, regulation of cellular response to oxidative stress, regulation of chemotaxis, regulation of DNA metabolic process, regulation of DNA replication, regulation of endothelial cell chemotaxis, regulation of endothelial cell migration, regulation of epithelial cell migration, regulation of glucose transport, regulation of MAP kinase activity, regulation of mitosis, regulation of mitotic cell cycle, regulation of morphogenesis of a branching structure, regulation of organ morphogenesis, regulation of peptidyl-serine phosphorylation, regulation of peptidyl-threonine phosphorylation, regulation of synaptic transmission, response to hydrogen peroxide, response to inorganic substance, response to oxidative stress, response to reactive oxygen species, skeletal muscle organ development, skeletal muscle tissue development, sperm part, striated muscle cell differentiation | http://www.ncbi.nlm.nih.gov/sites/entrez?db=gene&cmd=search&term=17295 |
| Ifng |  | 0.5978196003825157 | adaptive immune response, adaptive immune response based on somatic recombination of immune receptors built from immunoglobulin superfamily domains, alcohol biosynthetic process, alpha-beta T cell activation involved in immune response, alpha-beta T cell differentiation, alpha-beta T cell differentiation involved in immune response, amide transport, B cell activation, B cell activation involved in immune response, B cell mediated immunity, cell activation involved in immune response, cell chemotaxis, cell cycle arrest, cell killing, cellular response to biotic stimulus, cellular response to lipid, cellular response to lipopolysaccharide, cellular response to molecule of bacterial origin, cellular response to topologically incorrect protein, cellular response to unfolded protein, chemokine biosynthetic process, chemokine metabolic process, chemokine production, cytokine activity, cytokine biosynthetic process, cytokine metabolic process, cytokine secretion, defense response to bacterium, defense response to other organism, defense response to protozoan, disruption of cells of other organism, endoplasmic reticulum unfolded protein response, epithelial cell proliferation, ER-nucleus signaling pathway, establishment of protein localization to organelle, external side of plasma membrane, extrinsic apoptotic signaling pathway, fat-soluble vitamin biosynthetic process, fat-soluble vitamin metabolic process, granulocyte chemotaxis, granulocyte migration, heart contraction, heart process, homeostasis of number of cells, hormone secretion, hormone transport, immunoglobulin mediated immune response, immunoglobulin production, inflammatory cell apoptotic process, insulin secretion, interleukin-1 beta production, interleukin-1 beta secretion, interleukin-1 production, interleukin-1 secretion, interleukin-12 biosynthetic process, interleukin-12 production, interleukin-17 production, interleukin-6 biosynthetic process, interleukin-6 production, interspecies interaction between organisms, JAK-STAT cascade, kidney development, killing of cells of other organism, leukocyte activation involved in immune response, leukocyte apoptotic process, leukocyte chemotaxis, leukocyte homeostasis, leukocyte mediated immunity, leukocyte migration, leukocyte proliferation, lymphocyte activation involved in immune response, lymphocyte differentiation, lymphocyte mediated immunity, lymphocyte proliferation, membrane protein ectodomain proteolysis, membrane protein proteolysis, modification of morphology or physiology of other organism, mononuclear cell proliferation, multicellular organismal signaling, muscle cell apoptotic process, muscle cell proliferation, myeloid cell apoptotic process, myeloid cell differentiation, myeloid cell homeostasis, myeloid leukocyte differentiation, myeloid leukocyte migration, negative regulation of cell cycle, negative regulation of cell development, negative regulation of cytokine production, negative regulation of smooth muscle cell proliferation, neutrophil chemotaxis, neutrophil migration, nitric oxide biosynthetic process, nitric oxide metabolic process, nuclear import, organic hydroxy compound biosynthetic process, osteoclast differentiation, peptide hormone secretion, peptide secretion, peptide transport, peptidyl-serine modification, peptidyl-serine phosphorylation, peptidyl-tyrosine modification, peptidyl-tyrosine phosphorylation, positive regulation of B cell activation, positive regulation of catabolic process, positive regulation of cell activation, positive regulation of cell adhesion, positive regulation of cell killing, positive regulation of cellular catabolic process, positive regulation of cytokine biosynthetic process, positive regulation of cytokine production, positive regulation of cytokine secretion, positive regulation of DNA metabolic process, positive regulation of DNA recombination, positive regulation of immune effector process, positive regulation of interleukin-1 beta production, positive regulation of interleukin-1 beta secretion, positive regulation of interleukin-1 production, positive regulation of interleukin-1 secretion, positive regulation of isotype switching, positive regulation of JAK-STAT cascade, positive regulation of leukocyte activation, positive regulation of leukocyte differentiation, positive regulation of leukocyte proliferation, positive regulation of lipid metabolic process, positive regulation of lymphocyte activation, positive regulation of lymphocyte proliferation, positive regulation of membrane protein ectodomain proteolysis, positive regulation of mononuclear cell proliferation, positive regulation of monooxygenase activity, positive regulation of multi-organism process, positive regulation of myeloid cell differentiation, positive regulation of myeloid leukocyte differentiation, positive regulation of neuron differentiation, positive regulation of nitric oxide biosynthetic process, positive regulation of osteoclast differentiation, positive regulation of peptidyl-serine phosphorylation, positive regulation of peptidyl-tyrosine phosphorylation, positive regulation of protein catabolic process, positive regulation of protein processing, positive regulation of protein secretion, positive regulation of protein transport, positive regulation of proteolysis, positive regulation of secretion, positive regulation of stem cell proliferation, positive regulation of T cell activation, positive regulation of T cell proliferation, production of molecular mediator of immune response, protein import, protein import into nucleus, protein import into nucleus, translocation, protein localization to nucleus, protein secretion, protein targeting, protein targeting to nucleus, regulation of adaptive immune response, regulation of B cell activation, regulation of cell adhesion, regulation of cell killing, regulation of cell morphogenesis involved in differentiation, regulation of chemokine biosynthetic process, regulation of chemokine production, regulation of cytokine biosynthetic process, regulation of cytokine secretion, regulation of DNA metabolic process, regulation of epithelial cell differentiation, regulation of epithelial cell proliferation, regulation of heart contraction, regulation of hormone secretion, regulation of immune effector process, regulation of immunoglobulin production, regulation of insulin secretion, regulation of interleukin-1 beta secretion, regulation of interleukin-1 secretion, regulation of interleukin-12 biosynthetic process, regulation of interleukin-12 production, regulation of interleukin-17 production, regulation of interleukin-6 biosynthetic process, regulation of interleukin-6 production, regulation of isotype switching, regulation of JAK-STAT cascade, regulation of kidney development, regulation of leukocyte differentiation, regulation of leukocyte mediated immunity, regulation of leukocyte proliferation, regulation of lipid biosynthetic process, regulation of lipid metabolic process, regulation of lymphocyte activation, regulation of lymphocyte proliferation, regulation of membrane protein ectodomain proteolysis, regulation of metanephros development, regulation of mononuclear cell proliferation, regulation of monooxygenase activity, regulation of multi-organism process, regulation of muscle cell apoptotic process, regulation of myeloid cell differentiation, regulation of myeloid leukocyte differentiation, regulation of nitric oxide biosynthetic process, regulation of organ morphogenesis, regulation of osteoclast differentiation, regulation of oxidoreductase activity, regulation of peptide hormone secretion, regulation of peptide secretion, regulation of peptide transport, regulation of peptidyl-serine phosphorylation, regulation of peptidyl-tyrosine phosphorylation, regulation of production of molecular mediator of immune response, regulation of protein catabolic process, regulation of protein secretion, regulation of smooth muscle cell proliferation, regulation of stem cell proliferation, regulation of steroid biosynthetic process, regulation of steroid metabolic process, regulation of symbiosis, encompassing mutualism through parasitism, regulation of synaptic transmission, regulation of T cell activation, regulation of T cell proliferation, regulation of the force of heart contraction, regulation of tyrosine phosphorylation of STAT protein, regulation of vitamin metabolic process, response to bacterium, response to endoplasmic reticulum stress, response to lipopolysaccharide, response to molecule of bacterial origin, response to protozoan, response to topologically incorrect protein, response to unfolded protein, response to virus, single-organism nuclear import, smooth muscle cell proliferation, stem cell proliferation, steroid biosynthetic process, symbiosis, encompassing mutualism through parasitism, T cell activation involved in immune response, T cell differentiation, T cell differentiation involved in immune response, T cell proliferation, tyrosine phosphorylation of STAT protein, vitamin biosynthetic process, vitamin D biosynthetic process, vitamin D metabolic process, vitamin metabolic process | http://www.ncbi.nlm.nih.gov/sites/entrez?db=gene&cmd=search&term=15978 |
| Spp1 |  | 0.5947391530758838 | cell chemotaxis, cell-substrate adhesion, cytokine activity, granulocyte chemotaxis, granulocyte migration, leukocyte chemotaxis, leukocyte migration, myeloid leukocyte migration, neutrophil chemotaxis, neutrophil migration, positive regulation of cell adhesion, positive regulation of cell-substrate adhesion, regulation of cell adhesion, regulation of cell-substrate adhesion, response to alcohol, response to extracellular stimulus, response to nutrient, response to nutrient levels | http://www.ncbi.nlm.nih.gov/sites/entrez?db=gene&cmd=search&term=20750 |
| Map2k1 |  | 0.5923332833435819 | activation of MAPK activity, activation of protein kinase activity, adherens junction, aging, anchoring junction, cell aging, cell cortex, cell cycle arrest, cell projection cytoplasm, cell-cell adhesion, cellular senescence, cytoskeleton-dependent intracellular transport, dendrite cytoplasm, early endosome to late endosome transport, embryonic placenta development, epidermal cell differentiation, epidermis development, Golgi organization, homotypic cell-cell adhesion, keratinocyte differentiation, kinase regulator activity, late endosome, microtubule-based transport, mitogen-activated protein kinase kinase kinase binding, mitosis, negative regulation of cell adhesion, negative regulation of cell cycle, nuclear division, organelle transport along microtubule, pigmentation, placenta development, positive regulation of cell migration, positive regulation of cell motility, positive regulation of cellular component movement, positive regulation of MAP kinase activity, positive regulation of protein serine/threonine kinase activity, positive regulation of Ras protein signal transduction, protein kinase regulator activity, regulation of blood vessel size, regulation of cell adhesion, regulation of cell-cell adhesion, regulation of homotypic cell-cell adhesion, regulation of MAP kinase activity, regulation of smooth muscle contraction, regulation of stress-activated MAPK cascade, regulation of stress-activated protein kinase signaling cascade, regulation of transcription elongation from RNA polymerase II promoter, regulation of tube size, regulation of vasoconstriction, regulation of vesicle-mediated transport, response to axon injury, response to corticosteroid, response to glucocorticoid, response to oxidative stress, response to steroid hormone, skin development, smooth muscle contraction, stress-activated MAPK cascade, stress-activated protein kinase signaling cascade, vascular process in circulatory system, vasoconstriction, vesicle localization, vesicle transport along microtubule | http://www.ncbi.nlm.nih.gov/sites/entrez?db=gene&cmd=search&term=26395 |
| Nos2 |  | 0.591929222283204 | amide transport, beta-catenin binding, blood circulation, blood vessel remodeling, cadherin binding, cell adhesion molecule binding, cell cortex, cell killing, cellular response to biotic stimulus, cellular response to lipid, cellular response to lipopolysaccharide, cellular response to molecule of bacterial origin, defense response to bacterium, defense response to other organism, disruption of cells of other organism, heart contraction, heart process, heat shock protein binding, heme binding, hormone secretion, hormone transport, Hsp90 protein binding, innate immune response, insulin secretion, killing of cells of other organism, kinase regulator activity, modification of morphology or physiology of other organism, negative regulation of catabolic process, negative regulation of protein catabolic process, nitric oxide biosynthetic process, nitric oxide metabolic process, nitric-oxide synthase binding, oxidoreductase activity, acting on paired donors, with incorporation or reduction of molecular oxygen, oxidoreductase activity, acting on paired donors, with incorporation or reduction of molecular oxygen, NAD(P)H as one donor, and incorporation of one atom of oxygen, peptide hormone secretion, peptide secretion, peptide transport, positive regulation of cell killing, positive regulation of multi-organism process, positive regulation of vasodilation, protein kinase regulator activity, reactive oxygen species metabolic process, regulation of blood pressure, regulation of blood vessel size, regulation of cell killing, regulation of heart contraction, regulation of hormone secretion, regulation of insulin secretion, regulation of multi-organism process, regulation of peptide hormone secretion, regulation of peptide secretion, regulation of peptide transport, regulation of protein catabolic process, regulation of tube size, regulation of vasodilation, response to bacterium, response to decreased oxygen levels, response to hypoxia, response to lipopolysaccharide, response to molecule of bacterial origin, response to oxygen levels, response to tumor necrosis factor, superoxide metabolic process, tetrapyrrole binding, tissue remodeling, vascular process in circulatory system, vasodilation, vesicle membrane | http://www.ncbi.nlm.nih.gov/sites/entrez?db=gene&cmd=search&term=18126 |
| Cav1 |  | 0.591190950700888 | acrosomal vesicle, adherens junction, anchoring junction, anoikis, apical plasma membrane, basolateral plasma membrane, blood circulation, blood coagulation, blood vessel morphogenesis, BMP signaling pathway, body fluid secretion, calcium ion homeostasis, calcium ion transport, calcium ion transport into cytosol, canonical Wnt signaling pathway, caveola, cell cortex, cellular calcium ion homeostasis, cellular divalent inorganic cation homeostasis, cellular protein complex assembly, cellular response to external stimulus, cellular response to oxygen levels, coagulation, cytokine-mediated signaling pathway, cytosolic calcium ion homeostasis, cytosolic calcium ion transport, endothelial cell proliferation, epithelial cell proliferation, ERK1 and ERK2 cascade, extrinsic apoptotic signaling pathway, gland development, gland morphogenesis, heart contraction, heart process, hemostasis, intrinsic apoptotic signaling pathway, JAK-STAT cascade, lactation, lipid particle, lipid storage, lipid transport, maintenance of location, maintenance of location in cell, maintenance of protein location, maintenance of protein location in cell, mammary gland development, mammary gland morphogenesis, membrane depolarization, membrane raft, muscle cell apoptotic process, muscle cell proliferation, muscle organ development, negative regulation of anoikis, negative regulation of binding, negative regulation of BMP signaling pathway, negative regulation of canonical Wnt signaling pathway, negative regulation of endothelial cell proliferation, negative regulation of intracellular signal transduction, negative regulation of kinase activity, negative regulation of monooxygenase activity, negative regulation of muscle cell apoptotic process, negative regulation of neuron differentiation, negative regulation of oxidoreductase activity, negative regulation of peptidyl-serine phosphorylation, negative regulation of phosphorylation, negative regulation of protein binding, negative regulation of protein kinase activity, negative regulation of protein phosphorylation, negative regulation of smooth muscle cell proliferation, negative regulation of transferase activity, negative regulation of transmembrane receptor protein serine/threonine kinase signaling pathway, negative regulation of Wnt signaling pathway, nitric oxide biosynthetic process, nitric oxide metabolic process, nitric-oxide synthase binding, organic hydroxy compound transport, oxidoreductase activity, acting on paired donors, with incorporation or reduction of molecular oxygen, oxidoreductase activity, acting on paired donors, with incorporation or reduction of molecular oxygen, NAD(P)H as one donor, and incorporation of one atom of oxygen, peptidyl-serine modification, peptidyl-serine phosphorylation, peptidyl-tyrosine modification, peptidyl-tyrosine phosphorylation, positive regulation of apoptotic signaling pathway, positive regulation of canonical Wnt signaling pathway, positive regulation of cell activation, positive regulation of cytosolic calcium ion concentration, positive regulation of epithelial cell proliferation, positive regulation of extrinsic apoptotic signaling pathway, positive regulation of homeostatic process, positive regulation of intracellular transport, positive regulation of intrinsic apoptotic signaling pathway, positive regulation of ion transport, positive regulation of leukocyte activation, positive regulation of lymphocyte activation, positive regulation of microtubule polymerization, positive regulation of microtubule polymerization or depolymerization, positive regulation of organelle organization, positive regulation of peptidase activity, positive regulation of peptidyl-serine phosphorylation, positive regulation of protein complex assembly, positive regulation of T cell activation, positive regulation of vasoconstriction, positive regulation of Wnt signaling pathway, protein homooligomerization, receptor internalization, receptor metabolic process, regulation of anoikis, regulation of binding, regulation of blood coagulation, regulation of blood vessel size, regulation of BMP signaling pathway, regulation of body fluid levels, regulation of calcium ion transport, regulation of calcium ion transport into cytosol, regulation of canonical Wnt signaling pathway, regulation of coagulation, regulation of endocytosis, regulation of endothelial cell proliferation, regulation of epithelial cell differentiation, regulation of epithelial cell proliferation, regulation of ERK1 and ERK2 cascade, regulation of extrinsic apoptotic signaling pathway, regulation of heart contraction, regulation of hemostasis, regulation of homeostatic process, regulation of intrinsic apoptotic signaling pathway, regulation of ion homeostasis, regulation of JAK-STAT cascade, regulation of lipid metabolic process, regulation of lymphocyte activation, regulation of MAP kinase activity, regulation of metal ion transport, regulation of microtubule polymerization, regulation of monooxygenase activity, regulation of muscle cell apoptotic process, regulation of nitric oxide biosynthetic process, regulation of oxidoreductase activity, regulation of peptidyl-serine phosphorylation, regulation of peptidyl-tyrosine phosphorylation, regulation of protein binding, regulation of protein complex assembly, regulation of protein ubiquitination, regulation of smooth muscle cell proliferation, regulation of smooth muscle contraction, regulation of T cell activation, regulation of the force of heart contraction, regulation of transmembrane receptor protein serine/threonine kinase signaling pathway, regulation of tube size, regulation of tyrosine phosphorylation of STAT protein, regulation of vasoconstriction, regulation of vesicle-mediated transport, regulation of Wnt signaling pathway, regulation of wound healing, response to calcium ion, response to decreased oxygen levels, response to estrogen, response to hypoxia, response to inorganic substance, response to ketone, response to mechanical stimulus, response to metal ion, response to oxygen levels, response to steroid hormone, secretion by tissue, secretory granule, skeletal muscle organ development, skeletal muscle tissue development, smooth muscle cell proliferation, smooth muscle contraction, sperm part, tissue remodeling, transmembrane receptor protein serine/threonine kinase signaling pathway, tyrosine phosphorylation of STAT protein, vascular process in circulatory system, vasculogenesis, vasoconstriction, vesicle membrane, Wnt signaling pathway, wound healing | http://www.ncbi.nlm.nih.gov/sites/entrez?db=gene&cmd=search&term=12389 |
| Fas |  | 0.5902803000244414 | adaptive immune response, adaptive immune response based on somatic recombination of immune receptors built from immunoglobulin superfamily domains, apoptotic mitochondrial changes, B cell activation, B cell mediated immunity, cellular response to inorganic substance, cellular response to metal ion, cellular response to oxygen levels, cellular response to tumor necrosis factor, cytokine-mediated signaling pathway, external side of plasma membrane, extrinsic apoptotic signaling pathway, extrinsic apoptotic signaling pathway in absence of ligand, extrinsic apoptotic signaling pathway via death domain receptors, homeostasis of number of cells, immunoglobulin production, inflammatory cell apoptotic process, leukocyte apoptotic process, leukocyte homeostasis, leukocyte mediated immunity, lymphocyte apoptotic process, lymphocyte differentiation, lymphocyte homeostasis, lymphocyte mediated immunity, membrane raft, mitochondrion organization, myeloid cell differentiation, necroptotic process, necrotic cell death, negative regulation of cell activation, negative regulation of leukocyte activation, negative T cell selection, negative thymic T cell selection, neuron apoptotic process, neuron death, neuron projection regeneration, positive regulation of apoptotic signaling pathway, positive regulation of extrinsic apoptotic signaling pathway, positive regulation of extrinsic apoptotic signaling pathway in absence of ligand, positive regulation of mitochondrion organization, positive regulation of organelle organization, positive regulation of protein complex assembly, positive regulation of protein oligomerization, positive regulation of release of cytochrome c from mitochondria, production of molecular mediator of immune response, programmed necrotic cell death, protein homooligomerization, regeneration, regulation of B cell activation, regulation of extrinsic apoptotic signaling pathway, regulation of extrinsic apoptotic signaling pathway in absence of ligand, regulation of leukocyte differentiation, regulation of lymphocyte activation, regulation of lymphocyte differentiation, regulation of mitochondrion organization, regulation of myeloid cell differentiation, regulation of protein complex assembly, regulation of protein homooligomerization, regulation of protein oligomerization, regulation of release of cytochrome c from mitochondria, release of cytochrome c from mitochondria, response to corticosteroid, response to glucocorticoid, response to inorganic substance, response to metal ion, response to oxygen levels, response to steroid hormone, response to tumor necrosis factor, signal transduction in absence of ligand, spleen development, T cell apoptotic process, T cell differentiation, T cell differentiation in thymus, T cell homeostasis, T cell selection, thymic T cell selection, tumor necrosis factor-mediated signaling pathway | http://www.ncbi.nlm.nih.gov/sites/entrez?db=gene&cmd=search&term=14102 |
| Hspa5 |  | 0.5902002471575364 | cellular response to biotic stimulus, cellular response to external stimulus, cellular response to extracellular stimulus, cellular response to nutrient levels, cellular response to starvation, cellular response to topologically incorrect protein, cellular response to transforming growth factor beta stimulus, cellular response to unfolded protein, cerebellar Purkinje cell layer development, endoplasmic reticulum unfolded protein response, ER overload response, ER-nucleus signaling pathway, maintenance of location, maintenance of location in cell, maintenance of protein location, maintenance of protein location in cell, negative regulation of transmembrane receptor protein serine/threonine kinase signaling pathway, positive regulation of cell migration, positive regulation of cell motility, positive regulation of cellular component movement, regulation of embryonic development, regulation of protein ubiquitination, regulation of transmembrane receptor protein serine/threonine kinase signaling pathway, response to endoplasmic reticulum stress, response to extracellular stimulus, response to nutrient levels, response to starvation, response to topologically incorrect protein, response to transforming growth factor beta, response to unfolded protein, ribosome binding, small conjugating protein ligase binding, smooth endoplasmic reticulum, transforming growth factor beta receptor signaling pathway, transmembrane receptor protein serine/threonine kinase signaling pathway, ubiquitin protein ligase binding, unfolded protein binding | http://www.ncbi.nlm.nih.gov/sites/entrez?db=gene&cmd=search&term=14828 |
| Nfe2l2 |  | 0.589808993915431 | activating transcription factor binding, blood coagulation, cellular response to hydrogen peroxide, cellular response to oxidative stress, cellular response to reactive oxygen species, cellular response to topologically incorrect protein, cellular response to tumor necrosis factor, cellular response to unfolded protein, chromatin, coagulation, endoplasmic reticulum unfolded protein response, ER-nucleus signaling pathway, hemostasis, intrinsic apoptotic signaling pathway, negative regulation of apoptotic signaling pathway, negative regulation of cellular response to oxidative stress, negative regulation of intracellular signal transduction, negative regulation of intrinsic apoptotic signaling pathway, positive regulation of coagulation, positive regulation of reactive oxygen species metabolic process, reactive oxygen species metabolic process, regulation of blood coagulation, regulation of body fluid levels, regulation of cellular response to oxidative stress, regulation of coagulation, regulation of DNA-templated transcription in response to stress, regulation of embryonic development, regulation of hemostasis, regulation of intrinsic apoptotic signaling pathway, regulation of reactive oxygen species metabolic process, regulation of superoxide metabolic process, regulation of transcription from RNA polymerase II promoter in response to stress, regulation of wound healing, response to endoplasmic reticulum stress, response to hydrogen peroxide, response to inorganic substance, response to oxidative stress, response to reactive oxygen species, response to topologically incorrect protein, response to tumor necrosis factor, response to unfolded protein, RNA polymerase II activating transcription factor binding, RNA polymerase II transcription factor binding, superoxide metabolic process, wound healing | http://www.ncbi.nlm.nih.gov/sites/entrez?db=gene&cmd=search&term=18024 |
| Casp8 |  | 0.5868614681433935 | activation of cysteine-type endopeptidase activity, activation of cysteine-type endopeptidase activity involved in apoptotic process, blood vessel morphogenesis, cardiac muscle tissue development, cysteine-type endopeptidase activity, cysteine-type peptidase activity, cytokine receptor binding, embryonic epithelial tube formation, endopeptidase activity, epithelial tube formation, epithelial tube morphogenesis, execution phase of apoptosis, extrinsic apoptotic signaling pathway, extrinsic apoptotic signaling pathway via death domain receptors, I-kappaB kinase/NF-kappaB signaling, leukocyte apoptotic process, lymphocyte apoptotic process, macrophage differentiation, membrane raft, morphogenesis of embryonic epithelium, myeloid cell differentiation, myeloid leukocyte differentiation, necroptotic process, necrotic cell death, negative regulation of intracellular signal transduction, neural tube development, neural tube formation, positive regulation of apoptotic signaling pathway, positive regulation of cysteine-type endopeptidase activity, positive regulation of cysteine-type endopeptidase activity involved in apoptotic process, positive regulation of endopeptidase activity, positive regulation of extrinsic apoptotic signaling pathway, positive regulation of I-kappaB kinase/NF-kappaB signaling, positive regulation of leukocyte differentiation, positive regulation of macrophage differentiation, positive regulation of myeloid cell differentiation, positive regulation of myeloid leukocyte differentiation, positive regulation of peptidase activity, positive regulation of protein processing, positive regulation of proteolysis, programmed necrotic cell death, protease binding, regulation of cysteine-type endopeptidase activity, regulation of cysteine-type endopeptidase activity involved in apoptotic process, regulation of endopeptidase activity, regulation of extrinsic apoptotic signaling pathway, regulation of I-kappaB kinase/NF-kappaB signaling, regulation of leukocyte apoptotic process, regulation of leukocyte differentiation, regulation of lymphocyte apoptotic process, regulation of macrophage differentiation, regulation of myeloid cell differentiation, regulation of myeloid leukocyte differentiation, regulation of thymocyte apoptotic process, response to alcohol, response to tumor necrosis factor, small conjugating protein ligase binding, T cell apoptotic process, thymocyte apoptotic process, tube formation, tumor necrosis factor receptor binding, tumor necrosis factor receptor superfamily binding, ubiquitin protein ligase binding, zymogen activation | http://www.ncbi.nlm.nih.gov/sites/entrez?db=gene&cmd=search&term=12370 |
| Hmox1 |  | 0.5853600656136286 | blood circulation, blood vessel morphogenesis, caveola, cell activation involved in immune response, cellular response to inorganic substance, cellular response to metal ion, cellular response to oxidative stress, cytokine production involved in immune response, extrinsic apoptotic signaling pathway, extrinsic apoptotic signaling pathway via death domain receptors, heme binding, homeostasis of number of cells, I-kappaB kinase/NF-kappaB signaling, intrinsic apoptotic signaling pathway, intrinsic apoptotic signaling pathway in response to DNA damage, iron ion homeostasis, leukocyte activation involved in immune response, leukocyte mediated immunity, membrane raft, muscle cell apoptotic process, muscle cell proliferation, myeloid cell homeostasis, myeloid leukocyte activation, myeloid leukocyte cytokine production, negative regulation of apoptotic signaling pathway, negative regulation of binding, negative regulation of cell activation, negative regulation of cytokine production, negative regulation of cytokine production involved in immune response, negative regulation of DNA binding, negative regulation of exocytosis, negative regulation of extrinsic apoptotic signaling pathway, negative regulation of intracellular transport, negative regulation of leukocyte activation, negative regulation of muscle cell apoptotic process, negative regulation of neuron apoptotic process, negative regulation of neuron death, negative regulation of production of molecular mediator of immune response, negative regulation of secretion, negative regulation of smooth muscle cell proliferation, neuron apoptotic process, neuron death, oxidoreductase activity, acting on paired donors, with incorporation or reduction of molecular oxygen, positive regulation of I-kappaB kinase/NF-kappaB signaling, positive regulation of immune effector process, positive regulation of smooth muscle cell proliferation, production of molecular mediator of immune response, protein homooligomerization, regulation of angiogenesis, regulation of binding, regulation of blood pressure, regulation of cytokine production involved in immune response, regulation of DNA binding, regulation of DNA-templated transcription in response to stress, regulation of extrinsic apoptotic signaling pathway, regulation of I-kappaB kinase/NF-kappaB signaling, regulation of immune effector process, regulation of leukocyte mediated immunity, regulation of muscle cell apoptotic process, regulation of neuron apoptotic process, regulation of neuron death, regulation of production of molecular mediator of immune response, regulation of smooth muscle cell proliferation, regulation of transcription from RNA polymerase II promoter in response to stress, regulation of vasculature development, regulation of vesicle-mediated transport, response to cadmium ion, response to estrogen, response to hydrogen peroxide, response to inorganic substance, response to metal ion, response to oxidative stress, response to reactive oxygen species, response to steroid hormone, smooth muscle cell proliferation, tetrapyrrole binding, transition metal ion homeostasis, wound healing | http://www.ncbi.nlm.nih.gov/sites/entrez?db=gene&cmd=search&term=15368 |
| Cdkn1a |  | 0.5803601646324645 | aging, B cell activation, cell aging, cell cycle arrest, cellular response to external stimulus, cellular response to extracellular stimulus, cellular senescence, DNA biosynthetic process, DNA damage response, signal transduction by p53 class mediator, establishment of protein localization to organelle, fibroblast proliferation, intrinsic apoptotic signaling pathway, intrinsic apoptotic signaling pathway by p53 class mediator, intrinsic apoptotic signaling pathway in response to DNA damage, intrinsic apoptotic signaling pathway in response to DNA damage by p53 class mediator, kinase regulator activity, leukocyte proliferation, lymphocyte proliferation, mononuclear cell proliferation, negative regulation of cell cycle, negative regulation of kinase activity, negative regulation of phosphorylation, negative regulation of protein kinase activity, negative regulation of protein phosphorylation, negative regulation of transferase activity, nuclear import, positive regulation of B cell activation, positive regulation of cell activation, positive regulation of cell cycle arrest, positive regulation of cell cycle process, positive regulation of fibroblast proliferation, positive regulation of leukocyte activation, positive regulation of leukocyte proliferation, positive regulation of lymphocyte activation, positive regulation of lymphocyte proliferation, positive regulation of mononuclear cell proliferation, positive regulation of reactive oxygen species metabolic process, protein import, protein import into nucleus, protein import into nucleus, translocation, protein kinase regulator activity, protein localization to nucleus, reactive oxygen species metabolic process, regulation of B cell activation, regulation of DNA biosynthetic process, regulation of DNA metabolic process, regulation of fibroblast proliferation, regulation of intracellular protein transport, regulation of leukocyte proliferation, regulation of lymphocyte activation, regulation of lymphocyte proliferation, regulation of mitotic cell cycle, regulation of mononuclear cell proliferation, regulation of nucleocytoplasmic transport, regulation of protein import into nucleus, regulation of protein import into nucleus, translocation, regulation of protein localization to nucleus, regulation of reactive oxygen species metabolic process, response to extracellular stimulus, response to light stimulus, response to radiation, response to UV, signal transduction by p53 class mediator, signal transduction in response to DNA damage, single-organism nuclear import | http://www.ncbi.nlm.nih.gov/sites/entrez?db=gene&cmd=search&term=12575 |
| Il1b |  | 0.5774183779160216 | acid secretion, activation of MAPK activity, activation of protein kinase activity, acute inflammatory response, acute-phase response, aging, alcohol biosynthetic process, amide transport, amino acid transport, aminoglycan biosynthetic process, aminoglycan metabolic process, astrocyte differentiation, calcium ion homeostasis, carboxylic acid transport, cell chemotaxis, cell-cell adhesion, cellular calcium ion homeostasis, cellular divalent inorganic cation homeostasis, cellular process involved in reproduction in multicellular organism, cellular response to biotic stimulus, cellular response to hormone stimulus, cellular response to insulin stimulus, cellular response to lipid, cellular response to lipopolysaccharide, cellular response to molecule of bacterial origin, cellular response to nitrogen compound, cellular response to organic cyclic compound, cellular response to organonitrogen compound, cellular response to peptide, cellular response to peptide hormone stimulus, chemokine biosynthetic process, chemokine metabolic process, chemokine production, cognition, cytokine activity, cytokine biosynthetic process, cytokine metabolic process, cytokine-mediated signaling pathway, cytosolic calcium ion homeostasis, developmental programmed cell death, dicarboxylic acid transport, ERK1 and ERK2 cascade, establishment of protein localization to organelle, extrinsic apoptotic signaling pathway, extrinsic apoptotic signaling pathway in absence of ligand, fat-soluble vitamin biosynthetic process, fat-soluble vitamin metabolic process, fatty acid derivative transport, fatty acid transport, fever generation, glial cell differentiation, gliogenesis, glucose transport, glycosaminoglycan biosynthetic process, glycosaminoglycan metabolic process, granulocyte chemotaxis, granulocyte migration, heat generation, heterotypic cell-cell adhesion, hexose transport, homotypic cell-cell adhesion, hormone secretion, hormone transport, hyaluronan biosynthetic process, hyaluronan metabolic process, I-kappaB kinase/NF-kappaB signaling, icosanoid secretion, icosanoid transport, insulin receptor signaling pathway, insulin secretion, interleukin-1 beta production, interleukin-1 production, interleukin-6 biosynthetic process, interleukin-6 production, JNK cascade, learning or memory, leukocyte aggregation, leukocyte cell-cell adhesion, leukocyte chemotaxis, leukocyte migration, leukocyte proliferation, lipid storage, lipid transport, lipopolysaccharide-mediated signaling pathway, lymphocyte differentiation, lymphocyte proliferation, maintenance of location, membrane protein ectodomain proteolysis, membrane protein proteolysis, memory, mitosis, monocarboxylic acid transport, mononuclear cell proliferation, morphogenesis of a branching structure, multicellular organismal homeostasis, myeloid leukocyte migration, negative regulation of apoptotic signaling pathway, negative regulation of catabolic process, negative regulation of cell development, negative regulation of extrinsic apoptotic signaling pathway, negative regulation of glucose transport, negative regulation of insulin receptor signaling pathway, negative regulation of intracellular signal transduction, negative regulation of ion transport, negative regulation of kinase activity, negative regulation of lipid catabolic process, negative regulation of neural precursor cell proliferation, negative regulation of neurogenesis, negative regulation of neuron differentiation, negative regulation of phosphorylation, negative regulation of protein kinase activity, negative regulation of protein phosphorylation, negative regulation of secretion, negative regulation of transferase activity, neural precursor cell proliferation, neutrophil chemotaxis, neutrophil migration, NF-kappaB import into nucleus, nitric oxide biosynthetic process, nitric oxide metabolic process, nuclear division, nuclear import, organic acid transport, organic anion transport, organic hydroxy compound biosynthetic process, peptide hormone secretion, peptide secretion, peptide transport, positive regulation of acute inflammatory response, positive regulation of astrocyte differentiation, positive regulation of behavior, positive regulation of catabolic process, positive regulation of cell activation, positive regulation of cell adhesion, positive regulation of cell cycle, positive regulation of cell cycle process, positive regulation of cell development, positive regulation of cell migration, positive regulation of cell motility, positive regulation of cell-cell adhesion, positive regulation of cellular catabolic process, positive regulation of cellular component movement, positive regulation of chemokine production, positive regulation of chemotaxis, positive regulation of cytokine biosynthetic process, positive regulation of cytokine production, positive regulation of cytosolic calcium ion concentration, positive regulation of defense response, positive regulation of ERK1 and ERK2 cascade, positive regulation of fatty acid transport, positive regulation of glial cell differentiation, positive regulation of gliogenesis, positive regulation of granulocyte chemotaxis, positive regulation of heat generation, positive regulation of homeostatic process, positive regulation of I-kappaB kinase/NF-kappaB signaling, positive regulation of icosanoid secretion, positive regulation of inflammatory response, positive regulation of intracellular protein transport, positive regulation of intracellular transport, positive regulation of ion transport, positive regulation of JUN kinase activity, positive regulation of leukocyte activation, positive regulation of leukocyte proliferation, positive regulation of lipid metabolic process, positive regulation of lipid transport, positive regulation of lymphocyte activation, positive regulation of lymphocyte proliferation, positive regulation of MAP kinase activity, positive regulation of membrane protein ectodomain proteolysis, positive regulation of mononuclear cell proliferation, positive regulation of monooxygenase activity, positive regulation of neurogenesis, positive regulation of neutrophil chemotaxis, positive regulation of neutrophil migration, positive regulation of NF-kappaB import into nucleus, positive regulation of NF-kappaB transcription factor activity, positive regulation of nitric oxide biosynthetic process, positive regulation of nucleocytoplasmic transport, positive regulation of organelle organization, positive regulation of organic acid transport, positive regulation of prostaglandin secretion, positive regulation of protein catabolic process, positive regulation of protein import into nucleus, positive regulation of protein processing, positive regulation of protein serine/threonine kinase activity, positive regulation of protein transport, positive regulation of proteolysis, positive regulation of response to external stimulus, positive regulation of secretion, positive regulation of sequence-specific DNA binding transcription factor activity, positive regulation of T cell activation, positive regulation of T cell proliferation, positive regulation of transcription factor import into nucleus, positive regulation vascular endothelial growth factor production, prostaglandin secretion, prostaglandin transport, protein import, protein import into nucleus, protein kinase B signaling, protein localization to nucleus, regulation of acute inflammatory response, regulation of angiogenesis, regulation of anion transport, regulation of astrocyte differentiation, regulation of behavior, regulation of cell adhesion, regulation of cell division, regulation of cell-cell adhesion, regulation of cellular response to insulin stimulus, regulation of chemokine biosynthetic process, regulation of chemokine production, regulation of chemotaxis, regulation of cytokine biosynthetic process, regulation of ERK1 and ERK2 cascade, regulation of extrinsic apoptotic signaling pathway, regulation of extrinsic apoptotic signaling pathway in absence of ligand, regulation of fatty acid transport, regulation of fever generation, regulation of glial cell differentiation, regulation of gliogenesis, regulation of glucose transport, regulation of heat generation, regulation of heterotypic cell-cell adhesion, regulation of homeostatic process, regulation of hormone secretion, regulation of I-kappaB kinase/NF-kappaB signaling, regulation of icosanoid secretion, regulation of inflammatory response, regulation of insulin receptor signaling pathway, regulation of insulin secretion, regulation of interleukin-6 biosynthetic process, regulation of interleukin-6 production, regulation of intracellular protein transport, regulation of JNK cascade, regulation of JUN kinase activity, regulation of leukocyte differentiation, regulation of leukocyte proliferation, regulation of lipid biosynthetic process, regulation of lipid metabolic process, regulation of lipid transport, regulation of lymphocyte activation, regulation of lymphocyte differentiation, regulation of lymphocyte proliferation, regulation of MAP kinase activity, regulation of membrane protein ectodomain proteolysis, regulation of mitosis, regulation of mitotic cell cycle, regulation of mononuclear cell proliferation, regulation of monooxygenase activity, regulation of morphogenesis of a branching structure, regulation of neural precursor cell proliferation, regulation of neutrophil chemotaxis, regulation of neutrophil migration, regulation of NF-kappaB import into nucleus, regulation of nitric oxide biosynthetic process, regulation of nucleocytoplasmic transport, regulation of organic acid transport, regulation of oxidoreductase activity, regulation of peptide hormone secretion, regulation of peptide secretion, regulation of peptide transport, regulation of prostaglandin secretion, regulation of protein catabolic process, regulation of protein import into nucleus, regulation of protein localization to nucleus, regulation of steroid biosynthetic process, regulation of steroid metabolic process, regulation of stress-activated MAPK cascade, regulation of stress-activated protein kinase signaling cascade, regulation of T cell activation, regulation of T cell proliferation, regulation of transcription factor import into nucleus, regulation of vascular endothelial growth factor production, regulation of vasculature development, regulation of vitamin metabolic process, response to bacterium, response to drug, response to insulin, response to lipopolysaccharide, response to molecule of bacterial origin, response to organophosphorus, response to peptide, response to peptide hormone, response to purine-containing compound, secretory granule, signal transduction in absence of ligand, single-organism nuclear import, steroid biosynthetic process, stress-activated MAPK cascade, stress-activated protein kinase signaling cascade, T cell differentiation, T cell differentiation in thymus, T cell proliferation, temperature homeostasis, transcription factor import into nucleus, vascular endothelial growth factor production, vitamin biosynthetic process, vitamin D biosynthetic process, vitamin D metabolic process, vitamin metabolic process | http://www.ncbi.nlm.nih.gov/sites/entrez?db=gene&cmd=search&term=16176 |
| Il6 |  | 0.5716101578237777 | acute inflammatory response, adaptive immune response, adaptive immune response based on somatic recombination of immune receptors built from immunoglobulin superfamily domains, alpha-beta T cell activation involved in immune response, alpha-beta T cell differentiation, alpha-beta T cell differentiation involved in immune response, amide transport, anatomical structure homeostasis, B cell activation, branching involved in salivary gland morphogenesis, carbohydrate biosynthetic process, cell activation involved in immune response, cell fate commitment, cellular carbohydrate metabolic process, cellular response to biotic stimulus, cellular response to hydrogen peroxide, cellular response to interleukin-6, cellular response to lipid, cellular response to lipopolysaccharide, cellular response to molecule of bacterial origin, cellular response to oxidative stress, cellular response to reactive oxygen species, chemokine biosynthetic process, chemokine metabolic process, chemokine production, circadian rhythm, collagen metabolic process, cytokine activity, cytokine biosynthetic process, cytokine metabolic process, cytokine receptor binding, cytokine secretion, cytokine-mediated signaling pathway, defense response to other organism, defense response to protozoan, DNA replication, epithelial cell proliferation, ERK1 and ERK2 cascade, establishment of protein localization to organelle, external side of plasma membrane, gland development, gland morphogenesis, glucose metabolic process, hexose metabolic process, homeostasis of number of cells, hormone secretion, hormone transport, immunoglobulin production, immunoglobulin secretion, inflammatory cell apoptotic process, interleukin-6 production, interleukin-6-mediated signaling pathway, JAK-STAT cascade, leukocyte activation involved in immune response, leukocyte apoptotic process, leukocyte homeostasis, leukocyte proliferation, lymphocyte activation involved in immune response, lymphocyte differentiation, lymphocyte proliferation, mononuclear cell proliferation, monosaccharide metabolic process, morphogenesis of a branching epithelium, morphogenesis of a branching structure, multicellular organismal macromolecule metabolic process, multicellular organismal metabolic process, multicellular organismal signaling, muscle cell cellular homeostasis, muscle cell proliferation, muscle organ development, myeloid cell apoptotic process, myeloid cell homeostasis, negative regulation of carbohydrate metabolic process, negative regulation of cellular carbohydrate metabolic process, negative regulation of cysteine-type endopeptidase activity, negative regulation of cysteine-type endopeptidase activity involved in apoptotic process, negative regulation of cytokine biosynthetic process, negative regulation of endopeptidase activity, negative regulation of kinase activity, negative regulation of muscle organ development, negative regulation of neuron death, negative regulation of peptidase activity, negative regulation of phosphorylation, negative regulation of protein kinase activity, negative regulation of protein phosphorylation, negative regulation of secretion, negative regulation of transferase activity, neuron death, nitric oxide biosynthetic process, nitric oxide metabolic process, nuclear import, peptide hormone secretion, peptide secretion, peptide transport, peptidyl-serine modification, peptidyl-serine phosphorylation, peptidyl-tyrosine modification, peptidyl-tyrosine phosphorylation, positive regulation of acute inflammatory response, positive regulation of B cell activation, positive regulation of cell activation, positive regulation of chemokine production, positive regulation of cytokine production, positive regulation of defense response, positive regulation of DNA metabolic process, positive regulation of DNA replication, positive regulation of epithelial cell proliferation, positive regulation of ERK1 and ERK2 cascade, positive regulation of immune effector process, positive regulation of inflammatory response, positive regulation of intracellular protein transport, positive regulation of intracellular transport, positive regulation of JAK-STAT cascade, positive regulation of leukocyte activation, positive regulation of leukocyte differentiation, positive regulation of leukocyte proliferation, positive regulation of lymphocyte activation, positive regulation of lymphocyte proliferation, positive regulation of mononuclear cell proliferation, positive regulation of neuron differentiation, positive regulation of nitric oxide biosynthetic process, positive regulation of peptidyl-serine phosphorylation, positive regulation of peptidyl-tyrosine phosphorylation, positive regulation of protein kinase B signaling, positive regulation of protein secretion, positive regulation of protein transport, positive regulation of response to external stimulus, positive regulation of secretion, positive regulation of sequence-specific DNA binding transcription factor activity, positive regulation of smooth muscle cell proliferation, positive regulation of T cell activation, positive regulation of T cell proliferation, positive T cell selection, production of molecular mediator of immune response, protein import, protein import into nucleus, protein import into nucleus, translocation, protein kinase B signaling, protein localization to nucleus, protein secretion, receptor complex, regulation of acute inflammatory response, regulation of B cell activation, regulation of behavior, regulation of carbohydrate biosynthetic process, regulation of carbohydrate metabolic process, regulation of cellular carbohydrate metabolic process, regulation of chemokine biosynthetic process, regulation of chemokine production, regulation of collagen biosynthetic process, regulation of collagen metabolic process, regulation of cysteine-type endopeptidase activity, regulation of cysteine-type endopeptidase activity involved in apoptotic process, regulation of cytokine biosynthetic process, regulation of cytokine secretion, regulation of DNA metabolic process, regulation of DNA replication, regulation of endopeptidase activity, regulation of epithelial cell proliferation, regulation of ERK1 and ERK2 cascade, regulation of glucose metabolic process, regulation of hormone secretion, regulation of immune effector process, regulation of immunoglobulin production, regulation of immunoglobulin secretion, regulation of inflammatory response, regulation of interleukin-6 production, regulation of intracellular protein transport, regulation of JAK-STAT cascade, regulation of leukocyte differentiation, regulation of leukocyte proliferation, regulation of lymphocyte activation, regulation of lymphocyte differentiation, regulation of lymphocyte proliferation, regulation of mononuclear cell proliferation, regulation of muscle organ development, regulation of neuron death, regulation of nitric oxide biosynthetic process, regulation of nucleocytoplasmic transport, regulation of peptidyl-serine phosphorylation, regulation of peptidyl-tyrosine phosphorylation, regulation of production of molecular mediator of immune response, regulation of protein import into nucleus, regulation of protein import into nucleus, translocation, regulation of protein kinase B signaling, regulation of protein localization to nucleus, regulation of protein secretion, regulation of smooth muscle cell proliferation, regulation of T cell activation, regulation of T cell proliferation, regulation of type 2 immune response, regulation of tyrosine phosphorylation of STAT protein, regulation of vascular endothelial growth factor production, response to bacterium, response to corticosteroid, response to glucocorticoid, response to hydrogen peroxide, response to inorganic substance, response to interleukin-6, response to lipopolysaccharide, response to molecule of bacterial origin, response to oxidative stress, response to protozoan, response to reactive oxygen species, response to steroid hormone, response to virus, salivary gland morphogenesis, single-organism nuclear import, smooth muscle cell proliferation, T cell activation involved in immune response, T cell differentiation, T cell differentiation involved in immune response, T cell proliferation, T cell selection, T-helper 2 cell differentiation, type 2 immune response, tyrosine phosphorylation of STAT protein, vascular endothelial growth factor production | http://www.ncbi.nlm.nih.gov/sites/entrez?db=gene&cmd=search&term=16193 |
| Jun |  | 0.5714772849687233 | activating transcription factor binding, apoptotic mitochondrial changes, axon development, blood vessel morphogenesis, cellular response to calcium ion, cellular response to external stimulus, cellular response to extracellular stimulus, cellular response to inorganic substance, cellular response to metal ion, cellular response to nutrient levels, cellular response to starvation, cellular response to transforming growth factor beta stimulus, chromatin, cognition, DNA replication, double-stranded DNA binding, endothelial cell proliferation, epithelial cell proliferation, establishment of protein localization to organelle, euchromatin, fibroblast proliferation, heart morphogenesis, hepaticobiliary system development, interspecies interaction between organisms, learning, learning or memory, liver development, membrane depolarization, mitochondrion organization, modification of morphology or physiology of other organism, modification of morphology or physiology of other organism involved in symbiotic interaction, monocyte differentiation, multi-organism cellular process, muscle cell proliferation, myeloid cell differentiation, myeloid leukocyte activation, myeloid leukocyte differentiation, negative regulation of binding, negative regulation of DNA binding, negative regulation of neuron apoptotic process, negative regulation of neuron death, negative regulation of phosphorylation, negative regulation of protein phosphorylation, neuron apoptotic process, neuron death, neuron projection regeneration, nuclear chromatin, nuclear chromosome part, nuclear euchromatin, nuclear import, positive regulation of DNA metabolic process, positive regulation of DNA replication, positive regulation of epithelial cell proliferation, positive regulation of fibroblast proliferation, positive regulation of leukocyte differentiation, positive regulation of multi-organism process, positive regulation of myeloid cell differentiation, positive regulation of myeloid leukocyte differentiation, positive regulation of neuron apoptotic process, positive regulation of neuron death, positive regulation of smooth muscle cell proliferation, protein import, protein import into nucleus, protein localization to nucleus, protein targeting, protein targeting to nucleus, R-SMAD binding, regeneration, regulation of binding, regulation of DNA binding, regulation of DNA metabolic process, regulation of DNA replication, regulation of endothelial cell proliferation, regulation of epithelial cell proliferation, regulation of fibroblast proliferation, regulation of leukocyte differentiation, regulation of multi-organism process, regulation of myeloid cell differentiation, regulation of myeloid leukocyte differentiation, regulation of neuron apoptotic process, regulation of neuron death, regulation of smooth muscle cell proliferation, regulation of symbiosis, encompassing mutualism through parasitism, release of cytochrome c from mitochondria, response to axon injury, response to calcium ion, response to drug, response to extracellular stimulus, response to inorganic substance, response to metal ion, response to nutrient levels, response to radiation, response to starvation, response to transforming growth factor beta, RNA polymerase II activating transcription factor binding, RNA polymerase II core promoter proximal region sequence-specific DNA binding transcription factor activity involved in positive regulation of transcription, RNA polymerase II distal enhancer sequence-specific DNA binding, RNA polymerase II distal enhancer sequence-specific DNA binding transcription factor activity, RNA polymerase II regulatory region DNA binding, RNA polymerase II regulatory region sequence-specific DNA binding, RNA polymerase II transcription factor binding, sequence-specific DNA binding RNA polymerase II transcription factor activity, single-organism nuclear import, SMAD binding, SMAD protein import into nucleus, SMAD protein signal transduction, smooth muscle cell proliferation, structure-specific DNA binding, symbiosis, encompassing mutualism through parasitism, transcription regulatory region sequence-specific DNA binding, transforming growth factor beta receptor signaling pathway, transmembrane receptor protein serine/threonine kinase signaling pathway, viral process | http://www.ncbi.nlm.nih.gov/sites/entrez?db=gene&cmd=search&term=16476 |
| Src |  | 0.5707249409867434 | activation of protein kinase activity, anatomical structure homeostasis, anoikis, bone remodeling, bone resorption, branching involved in mammary gland duct morphogenesis, branching morphogenesis of an epithelial tube, canonical Wnt signaling pathway, caveola, cell adhesion molecule binding, cell junction assembly, cell junction organization, cell-cell junction organization, cell-matrix adhesion, cell-substrate adhesion, cellular carbohydrate metabolic process, cellular response to hormone stimulus, cellular response to insulin stimulus, cellular response to ketone, cellular response to lipid, cellular response to nitrogen compound, cellular response to organic cyclic compound, cellular response to organonitrogen compound, cellular response to peptide, cellular response to peptide hormone stimulus, cytokine secretion, DNA biosynthetic process, epithelial tube morphogenesis, ERK1 and ERK2 cascade, extrinsic apoptotic signaling pathway, forebrain development, gland development, gland morphogenesis, glucose metabolic process, heme binding, hexose metabolic process, insulin receptor signaling pathway, intrinsic apoptotic signaling pathway, late endosome, mammary gland development, mammary gland duct morphogenesis, mammary gland epithelium development, mammary gland morphogenesis, membrane depolarization, membrane raft, monosaccharide metabolic process, morphogenesis of a branching epithelium, morphogenesis of a branching structure, multicellular organismal homeostasis, muscle cell migration, negative regulation of anoikis, negative regulation of apoptotic signaling pathway, negative regulation of cell adhesion, negative regulation of cysteine-type endopeptidase activity, negative regulation of cysteine-type endopeptidase activity involved in apoptotic process, negative regulation of endopeptidase activity, negative regulation of extrinsic apoptotic signaling pathway, negative regulation of intracellular signal transduction, negative regulation of intrinsic apoptotic signaling pathway, negative regulation of peptidase activity, organelle assembly, peptidyl-serine modification, peptidyl-serine phosphorylation, peptidyl-tyrosine modification, peptidyl-tyrosine phosphorylation, phosphoprotein binding, positive regulation of canonical Wnt signaling pathway, positive regulation of carbohydrate metabolic process, positive regulation of cell adhesion, positive regulation of cell cycle, positive regulation of cell migration, positive regulation of cell motility, positive regulation of cellular component movement, positive regulation of cytokine secretion, positive regulation of DNA biosynthetic process, positive regulation of DNA metabolic process, positive regulation of ERK1 and ERK2 cascade, positive regulation of lipid kinase activity, positive regulation of lipid metabolic process, positive regulation of MAP kinase activity, positive regulation of phosphatidylinositol 3-kinase activity, positive regulation of protein complex assembly, positive regulation of protein kinase B signaling, positive regulation of protein secretion, positive regulation of protein serine/threonine kinase activity, positive regulation of protein transport, positive regulation of secretion, positive regulation of Wnt signaling pathway, protein C-terminus binding, protein homooligomerization, protein kinase B signaling, protein kinase C binding, protein secretion, regulation of anoikis, regulation of binding, regulation of canonical Wnt signaling pathway, regulation of carbohydrate metabolic process, regulation of cell adhesion, regulation of cell-matrix adhesion, regulation of cell-substrate adhesion, regulation of cellular carbohydrate metabolic process, regulation of cellular response to insulin stimulus, regulation of cysteine-type endopeptidase activity, regulation of cysteine-type endopeptidase activity involved in apoptotic process, regulation of cytokine secretion, regulation of DNA biosynthetic process, regulation of DNA metabolic process, regulation of endopeptidase activity, regulation of ERK1 and ERK2 cascade, regulation of extrinsic apoptotic signaling pathway, regulation of glucose metabolic process, regulation of homeostatic process, regulation of insulin receptor signaling pathway, regulation of intrinsic apoptotic signaling pathway, regulation of ion homeostasis, regulation of lipid kinase activity, regulation of lipid metabolic process, regulation of MAP kinase activity, regulation of membrane depolarization, regulation of mitochondrial membrane potential, regulation of phosphatidylinositol 3-kinase activity, regulation of protein binding, regulation of protein complex assembly, regulation of protein homooligomerization, regulation of protein kinase B signaling, regulation of protein oligomerization, regulation of protein secretion, regulation of Wnt signaling pathway, reproductive structure development, reproductive system development, response to insulin, response to interleukin-1, response to ketone, response to peptide, response to peptide hormone, response to steroid hormone, steroid hormone receptor binding, tetrapyrrole binding, tissue homeostasis, tissue remodeling, Wnt signaling pathway | http://www.ncbi.nlm.nih.gov/sites/entrez?db=gene&cmd=search&term=20779 |
| Tgfb1 |  | 0.5697157431989248 | adaptive immune response, adaptive immune response based on somatic recombination of immune receptors built from immunoglobulin superfamily domains, ameboidal cell migration, aminoglycan biosynthetic process, aminoglycan catabolic process, aminoglycan metabolic process, ATP biosynthetic process, B cell activation, B cell activation involved in immune response, B cell mediated immunity, blood vessel endothelial cell migration, branching involved in mammary gland duct morphogenesis, branching involved in ureteric bud morphogenesis, branching morphogenesis of an epithelial tube, calcium ion homeostasis, calcium ion transport, calcium ion transport into cytosol, carbohydrate biosynthetic process, cartilage development, cell activation involved in immune response, cell cycle arrest, cell junction organization, cell-cell adhesion, cell-cell junction organization, cellular calcium ion homeostasis, cellular divalent inorganic cation homeostasis, cellular process involved in reproduction in multicellular organism, cellular protein complex assembly, cellular response to biotic stimulus, cellular response to hormone stimulus, cellular response to ketone, cellular response to lipid, cellular response to lipopolysaccharide, cellular response to molecule of bacterial origin, cellular response to organic cyclic compound, cellular response to transforming growth factor beta stimulus, collagen metabolic process, connective tissue development, cytokine production involved in immune response, cytokine receptor binding, cytosolic calcium ion homeostasis, cytosolic calcium ion transport, defense response to other organism, dendritic cell differentiation, development of secondary sexual characteristics, developmental growth involved in morphogenesis, DNA replication, endoderm development, endothelial cell migration, epithelial cell migration, epithelial cell proliferation, epithelial to mesenchymal transition, epithelial tube morphogenesis, establishment of protein localization to organelle, extracellular matrix assembly, extrinsic apoptotic signaling pathway, fat cell differentiation, gland development, gland morphogenesis, glycosaminoglycan biosynthetic process, glycosaminoglycan catabolic process, glycosaminoglycan metabolic process, hematopoietic progenitor cell differentiation, homeostasis of number of cells, hyaluronan biosynthetic process, hyaluronan metabolic process, immunoglobulin mediated immune response, immunoglobulin production, innate immune response, interaction with host, interleukin-17 production, interspecies interaction between organisms, kidney development, leukocyte activation involved in immune response, leukocyte homeostasis, leukocyte mediated immunity, leukocyte proliferation, lipopolysaccharide-mediated signaling pathway, lymph node development, lymphocyte activation involved in immune response, lymphocyte differentiation, lymphocyte homeostasis, lymphocyte mediated immunity, lymphocyte proliferation, macrophage cytokine production, mammary gland development, mammary gland duct morphogenesis, mammary gland epithelium development, mammary gland morphogenesis, mesenchymal cell development, mesenchymal cell differentiation, mesenchyme development, mitosis, modification by symbiont of host morphology or physiology, modification of morphology or physiology of other organism, modification of morphology or physiology of other organism involved in symbiotic interaction, modulation by virus of host morphology or physiology, mononuclear cell proliferation, morphogenesis of a branching epithelium, morphogenesis of a branching structure, multi-organism cellular process, multicellular organismal macromolecule metabolic process, multicellular organismal metabolic process, muscle cell development, muscle cell differentiation, muscle organ development, myeloid cell differentiation, myeloid dendritic cell activation, myeloid dendritic cell differentiation, myeloid leukocyte activation, myeloid leukocyte cytokine production, myeloid leukocyte differentiation, myotube differentiation, negative regulation of blood vessel endothelial cell migration, negative regulation of calcium ion transport, negative regulation of carbohydrate metabolic process, negative regulation of cell activation, negative regulation of cell adhesion, negative regulation of cell cycle, negative regulation of cell development, negative regulation of cytokine production, negative regulation of cytokine production involved in immune response, negative regulation of intracellular transport, negative regulation of ion transport, negative regulation of leukocyte activation, negative regulation of muscle cell differentiation, negative regulation of muscle organ development, negative regulation of muscle tissue development, negative regulation of myoblast differentiation, negative regulation of neural precursor cell proliferation, negative regulation of neurogenesis, negative regulation of ossification, negative regulation of phosphorylation, negative regulation of production of molecular mediator of immune response, negative regulation of protein phosphorylation, negative regulation of sequestering of calcium ion, negative regulation of striated muscle tissue development, neural precursor cell proliferation, neuroblast proliferation, nuclear division, nuclear import, odontogenesis, ossification, peptidyl-serine modification, peptidyl-serine phosphorylation, peptidyl-threonine modification, peptidyl-threonine phosphorylation, positive regulation of B cell activation, positive regulation of behavior, positive regulation of binding, positive regulation of blood vessel endothelial cell migration, positive regulation of cell activation, positive regulation of cell cycle arrest, positive regulation of cell cycle process, positive regulation of cell development, positive regulation of cell migration, positive regulation of cell motility, positive regulation of cellular component movement, positive regulation of chemotaxis, positive regulation of cytokine production, positive regulation of cytosolic calcium ion concentration, positive regulation of DNA metabolic process, positive regulation of DNA recombination, positive regulation of endothelial cell migration, positive regulation of epithelial cell migration, positive regulation of epithelial cell proliferation, positive regulation of epithelial to mesenchymal transition, positive regulation of immune effector process, positive regulation of intracellular protein transport, positive regulation of intracellular transport, positive regulation of isotype switching, positive regulation of leukocyte activation, positive regulation of lipid kinase activity, positive regulation of lipid metabolic process, positive regulation of lymphocyte activation, positive regulation of MAP kinase activity, positive regulation of NF-kappaB transcription factor activity, positive regulation of nucleocytoplasmic transport, positive regulation of organelle organization, positive regulation of peptidyl-serine phosphorylation, positive regulation of peptidyl-threonine phosphorylation, positive regulation of phosphatidylinositol 3-kinase activity, positive regulation of protein complex assembly, positive regulation of protein import into nucleus, positive regulation of protein kinase B signaling, positive regulation of protein secretion, positive regulation of protein serine/threonine kinase activity, positive regulation of protein transport, positive regulation of reactive oxygen species metabolic process, positive regulation of response to external stimulus, positive regulation of secretion, positive regulation of sequence-specific DNA binding transcription factor activity, production of molecular mediator of immune response, protein import, protein import into nucleus, protein import into nucleus, translocation, protein kinase B signaling, protein localization to nucleus, protein N-terminus binding, protein secretion, protein targeting, protein targeting to nucleus, proteinaceous extracellular matrix, purine nucleoside triphosphate biosynthetic process, purine ribonucleoside triphosphate biosynthetic process, reactive oxygen species metabolic process, receptor metabolic process, regeneration, regulation of adaptive immune response, regulation of B cell activation, regulation of behavior, regulation of binding, regulation of blood vessel endothelial cell migration, regulation of calcium ion transport, regulation of calcium ion transport into cytosol, regulation of carbohydrate biosynthetic process, regulation of carbohydrate metabolic process, regulation of cell adhesion, regulation of cell division, regulation of cell morphogenesis involved in differentiation, regulation of cell-cell adhesion, regulation of chemotaxis, regulation of collagen biosynthetic process, regulation of collagen metabolic process, regulation of cytokine production involved in immune response, regulation of DNA binding, regulation of DNA metabolic process, regulation of DNA replication, regulation of endocytosis, regulation of endothelial cell migration, regulation of epithelial cell migration, regulation of epithelial cell proliferation, regulation of fat cell differentiation, regulation of histone deacetylation, regulation of homeostatic process, regulation of immune effector process, regulation of immunoglobulin production, regulation of interleukin-17 production, regulation of intracellular protein transport, regulation of ion homeostasis, regulation of isotype switching, regulation of kidney development, regulation of leukocyte mediated immunity, regulation of leukocyte proliferation, regulation of lipid kinase activity, regulation of lipid metabolic process, regulation of lymphocyte activation, regulation of lymphocyte proliferation, regulation of macrophage cytokine production, regulation of MAP kinase activity, regulation of metal ion transport, regulation of mitosis, regulation of mitotic cell cycle, regulation of mononuclear cell proliferation, regulation of morphogenesis of a branching structure, regulation of muscle organ development, regulation of muscle tissue development, regulation of neural precursor cell proliferation, regulation of neuroblast proliferation, regulation of nucleocytoplasmic transport, regulation of odontogenesis, regulation of organ morphogenesis, regulation of ossification, regulation of peptidyl-serine phosphorylation, regulation of peptidyl-threonine phosphorylation, regulation of phosphatidylinositol 3-kinase activity, regulation of production of molecular mediator of immune response, regulation of protein complex assembly, regulation of protein deacetylation, regulation of protein import into nucleus, regulation of protein kinase B signaling, regulation of protein localization to nucleus, regulation of protein secretion, regulation of reactive oxygen species metabolic process, regulation of sequestering of calcium ion, regulation of skeletal muscle tissue development, regulation of stem cell proliferation, regulation of striated muscle cell differentiation, regulation of striated muscle tissue development, regulation of superoxide metabolic process, regulation of T cell activation, regulation of T cell proliferation, regulation of transcription regulatory region DNA binding, regulation of transmembrane receptor protein serine/threonine kinase signaling pathway, regulation of vesicle-mediated transport, release of sequestered calcium ion into cytosol, response to alcohol, response to bacterium, response to corticosteroid, response to estrogen, response to glucocorticoid, response to ketone, response to lipopolysaccharide, response to molecule of bacterial origin, response to steroid hormone, response to transforming growth factor beta, ribonucleoside triphosphate biosynthetic process, secretory granule, sex differentiation, single-organism nuclear import, skeletal muscle organ development, skeletal muscle tissue development, SMAD protein import into nucleus, stem cell development, stem cell differentiation, stem cell proliferation, striated muscle cell development, striated muscle cell differentiation, superoxide metabolic process, symbiosis, encompassing mutualism through parasitism, T cell differentiation, T cell homeostasis, T cell proliferation, transforming growth factor beta receptor signaling pathway, transmembrane receptor protein serine/threonine kinase signaling pathway, viral process, wound healing | http://www.ncbi.nlm.nih.gov/sites/entrez?db=gene&cmd=search&term=21803 |
| Stat3 |  | 0.5671736714657156 | astrocyte differentiation, CCR chemokine receptor binding, cellular carbohydrate catabolic process, cellular carbohydrate metabolic process, cellular response to hormone stimulus, cellular response to interleukin-6, cellular response to nitrogen compound, cellular response to organonitrogen compound, cellular response to peptide, cellular response to peptide hormone stimulus, cytokine receptor binding, cytokine-mediated signaling pathway, establishment of protein localization to organelle, glial cell differentiation, gliogenesis, glucocorticoid receptor binding, glucose catabolic process, glucose metabolic process, glycolysis, hexose catabolic process, hexose metabolic process, interleukin-6-mediated signaling pathway, JAK-STAT cascade, monosaccharide catabolic process, monosaccharide metabolic process, multicellular organismal homeostasis, negative regulation of carbohydrate metabolic process, negative regulation of catabolic process, negative regulation of cellular carbohydrate metabolic process, neuron migration, nuclear import, protein import, protein import into nucleus, protein localization to nucleus, protein targeting, protein targeting to nucleus, regulation of carbohydrate catabolic process, regulation of carbohydrate metabolic process, regulation of cellular carbohydrate catabolic process, regulation of cellular carbohydrate metabolic process, regulation of generation of precursor metabolites and energy, regulation of glucose metabolic process, regulation of glycolysis, repressing transcription factor binding, response to alcohol, response to estrogen, response to interleukin-6, response to peptide, response to peptide hormone, response to steroid hormone, RNA polymerase II transcription factor binding, sequence-specific DNA binding RNA polymerase II transcription factor activity, single-organism carbohydrate catabolic process, single-organism nuclear import, stem cell development, stem cell differentiation, steroid hormone receptor binding, temperature homeostasis | http://www.ncbi.nlm.nih.gov/sites/entrez?db=gene&cmd=search&term=20848 |
| Hif1a |  | 0.5669802639712578 | ameboidal cell migration, amide transport, anatomical structure homeostasis, B cell homeostasis, biomineral tissue development, blood vessel morphogenesis, body fluid secretion, bone mineralization, cartilage development, cell maturation, cellular carbohydrate catabolic process, cellular carbohydrate metabolic process, cellular iron ion homeostasis, cellular response to oxidative stress, cellular response to oxygen levels, cerebral cortex development, cognition, collagen metabolic process, connective tissue development, cytoskeleton-dependent intracellular transport, determination of bilateral symmetry, determination of heart left/right asymmetry, determination of left/right symmetry, developmental maturation, embryonic epithelial tube formation, embryonic heart tube development, embryonic heart tube morphogenesis, embryonic organ morphogenesis, embryonic placenta development, epithelial cell maturation, epithelial cell migration, epithelial to mesenchymal transition, epithelial tube formation, epithelial tube morphogenesis, forebrain development, gland development, glucose catabolic process, glucose metabolic process, glycolysis, heart looping, heart morphogenesis, heat shock protein binding, hexose catabolic process, hexose metabolic process, histone acetyltransferase binding, histone deacetylase binding, homeostasis of number of cells, hormone secretion, hormone transport, Hsp90 protein binding, insulin secretion, iron ion homeostasis, lactation, learning, learning or memory, leukocyte apoptotic process, leukocyte homeostasis, lymphocyte apoptotic process, lymphocyte homeostasis, mammary gland alveolus development, mammary gland development, mammary gland epithelial cell differentiation, mammary gland epithelium development, mammary gland lobule development, mesenchymal cell development, mesenchymal cell differentiation, mesenchyme development, microtubule-based transport, monosaccharide catabolic process, monosaccharide metabolic process, morphogenesis of embryonic epithelium, multicellular organismal macromolecule metabolic process, multicellular organismal metabolic process, muscle cell cellular homeostasis, muscle cell proliferation, myeloid cell differentiation, negative regulation of intracellular signal transduction, negative regulation of leukocyte apoptotic process, negative regulation of lymphocyte apoptotic process, negative regulation of neuron apoptotic process, negative regulation of neuron death, negative regulation of ossification, neural precursor cell proliferation, neural tube development, neural tube formation, neuroblast proliferation, neuron apoptotic process, neuron death, ossification, pallium development, peptide hormone secretion, peptide secretion, peptide transport, placenta development, positive regulation of cell development, positive regulation of cell migration, positive regulation of cell motility, positive regulation of cellular component movement, positive regulation of cytokine production, positive regulation of epithelial cell migration, positive regulation of myeloid cell differentiation, positive regulation of neuroblast proliferation, positive regulation of neurogenesis, positive regulation of secretion, positive regulation of smooth muscle cell proliferation, positive regulation of stem cell proliferation, positive regulation vascular endothelial growth factor production, primary neural tube formation, receptor biosynthetic process, receptor metabolic process, regulation of blood vessel size, regulation of body fluid levels, regulation of carbohydrate catabolic process, regulation of carbohydrate metabolic process, regulation of cellular carbohydrate catabolic process, regulation of cellular carbohydrate metabolic process, regulation of DNA-templated transcription in response to stress, regulation of epithelial cell migration, regulation of generation of precursor metabolites and energy, regulation of glucose metabolic process, regulation of glycolysis, regulation of homeostatic process, regulation of hormone secretion, regulation of insulin secretion, regulation of leukocyte apoptotic process, regulation of lymphocyte apoptotic process, regulation of myeloid cell differentiation, regulation of neural precursor cell proliferation, regulation of neuroblast proliferation, regulation of neuron apoptotic process, regulation of neuron death, regulation of ossification, regulation of peptide hormone secretion, regulation of peptide secretion, regulation of peptide transport, regulation of receptor biosynthetic process, regulation of smooth muscle cell proliferation, regulation of stem cell proliferation, regulation of thymocyte apoptotic process, regulation of transcription elongation from RNA polymerase II promoter, regulation of transcription from RNA polymerase II promoter in response to stress, regulation of transforming growth factor beta production, regulation of tube size, regulation of vascular endothelial growth factor production, regulation of vasoconstriction, response to decreased oxygen levels, response to hypoxia, response to light stimulus, response to oxidative stress, response to oxygen levels, response to radiation, RNA polymerase II core promoter proximal region sequence-specific DNA binding transcription factor activity involved in positive regulation of transcription, RNA polymerase II distal enhancer sequence-specific DNA binding transcription factor activity, secretion by tissue, sequence-specific DNA binding RNA polymerase II transcription factor activity, single-organism carbohydrate catabolic process, small conjugating protein ligase binding, smooth muscle cell proliferation, specification of symmetry, stem cell development, stem cell differentiation, stem cell proliferation, T cell apoptotic process, telencephalon development, thymocyte apoptotic process, tissue remodeling, transforming growth factor beta production, transition metal ion homeostasis, tube formation, ubiquitin protein ligase binding, vascular endothelial growth factor production, vascular process in circulatory system, vasoconstriction, wound healing | http://www.ncbi.nlm.nih.gov/sites/entrez?db=gene&cmd=search&term=15251 |
| Cebpb |  | 0.5648273771680631 | cellular response to nitrogen compound, cellular response to organonitrogen compound, chromatin, cytokine biosynthetic process, cytokine metabolic process, embryonic placenta development, epithelial cell proliferation, fat cell differentiation, gland development, glucocorticoid receptor binding, interleukin-6 biosynthetic process, interleukin-6 production, mammary gland development, mammary gland epithelial cell differentiation, mammary gland epithelial cell proliferation, mammary gland epithelium development, negative regulation of neuron apoptotic process, negative regulation of neuron death, neuron apoptotic process, neuron death, nuclear chromatin, nuclear chromosome part, ossification, osteoblast differentiation, placenta development, positive regulation of fat cell differentiation, regulation of cytokine biosynthetic process, regulation of fat cell differentiation, regulation of interleukin-6 biosynthetic process, regulation of interleukin-6 production, regulation of neuron apoptotic process, regulation of neuron death, regulation of ossification, response to bacterium, response to endoplasmic reticulum stress, response to lipopolysaccharide, response to molecule of bacterial origin, RNA polymerase II core promoter proximal region sequence-specific DNA binding transcription factor activity involved in positive regulation of transcription, RNA polymerase II distal enhancer sequence-specific DNA binding transcription factor activity, sequence-specific DNA binding RNA polymerase II transcription factor activity, steroid hormone receptor binding | http://www.ncbi.nlm.nih.gov/sites/entrez?db=gene&cmd=search&term=12608 |
| Tbk1 |  | 0.5546917815290975 | activation of immune response, cytokine biosynthetic process, cytokine metabolic process, defense response to bacterium, defense response to other organism, innate immune response, leukocyte proliferation, lymphocyte proliferation, mononuclear cell proliferation, phosphoprotein binding, positive regulation of cytokine biosynthetic process, positive regulation of cytokine production, positive regulation of defense response, positive regulation of immune response, regulation of cytokine biosynthetic process, response to bacterium | http://www.ncbi.nlm.nih.gov/sites/entrez?db=gene&cmd=search&term=56480 |
| Myc |  | 0.5457825192636163 | acid secretion, activation of cysteine-type endopeptidase activity, activation of cysteine-type endopeptidase activity involved in apoptotic process, amino acid transport, anatomical structure homeostasis, ATP biosynthetic process, B cell apoptotic process, branching involved in ureteric bud morphogenesis, branching morphogenesis of an epithelial tube, canonical Wnt signaling pathway, carboxylic acid transport, cell cycle arrest, cellular carbohydrate catabolic process, cellular carbohydrate metabolic process, cellular iron ion homeostasis, core promoter proximal region DNA binding, core promoter proximal region sequence-specific DNA binding, DNA biosynthetic process, double-stranded DNA binding, embryonic organ morphogenesis, epithelial cell proliferation, epithelial tube morphogenesis, fibroblast proliferation, glucose catabolic process, glucose metabolic process, glucose transport, glycolysis, hexose catabolic process, hexose metabolic process, hexose transport, inner mitochondrial membrane organization, interaction with host, interspecies interaction between organisms, intrinsic apoptotic signaling pathway, intrinsic apoptotic signaling pathway in response to DNA damage, iron ion homeostasis, kidney development, leukocyte apoptotic process, lymphocyte apoptotic process, mitochondrial membrane organization, mitochondrion organization, modification by symbiont of host morphology or physiology, modification of morphology or physiology of other organism, modification of morphology or physiology of other organism involved in symbiotic interaction, modulation by virus of host morphology or physiology, monocarboxylic acid transport, monocyte differentiation, monosaccharide catabolic process, monosaccharide metabolic process, morphogenesis of a branching epithelium, morphogenesis of a branching structure, multi-organism cellular process, muscle organ development, myeloid cell differentiation, myeloid leukocyte differentiation, negative regulation of binding, negative regulation of cell cycle, negative regulation of fibroblast proliferation, negative regulation of glucose transport, negative regulation of protein binding, organic acid transport, organic anion transport, organic hydroxy compound transport, pigmentation, positive regulation of apoptotic signaling pathway, positive regulation of carbohydrate metabolic process, positive regulation of catabolic process, positive regulation of cell cycle, positive regulation of cellular catabolic process, positive regulation of cysteine-type endopeptidase activity, positive regulation of cysteine-type endopeptidase activity involved in apoptotic process, positive regulation of DNA biosynthetic process, positive regulation of DNA metabolic process, positive regulation of endopeptidase activity, positive regulation of epithelial cell proliferation, positive regulation of fibroblast proliferation, positive regulation of leukocyte apoptotic process, positive regulation of lymphocyte apoptotic process, positive regulation of membrane potential, positive regulation of peptidase activity, positive regulation of stem cell proliferation, purine nucleoside triphosphate biosynthetic process, purine ribonucleoside triphosphate biosynthetic process, regulation of B cell apoptotic process, regulation of binding, regulation of carbohydrate catabolic process, regulation of carbohydrate metabolic process, regulation of cell division, regulation of cellular carbohydrate catabolic process, regulation of cellular carbohydrate metabolic process, regulation of cysteine-type endopeptidase activity, regulation of cysteine-type endopeptidase activity involved in apoptotic process, regulation of DNA biosynthetic process, regulation of DNA metabolic process, regulation of endopeptidase activity, regulation of epithelial cell proliferation, regulation of fibroblast proliferation, regulation of generation of precursor metabolites and energy, regulation of glucose metabolic process, regulation of glucose transport, regulation of glycolysis, regulation of homeostatic process, regulation of kidney development, regulation of leukocyte apoptotic process, regulation of leukocyte differentiation, regulation of lymphocyte apoptotic process, regulation of metanephros development, regulation of mitochondrial membrane potential, regulation of mitotic cell cycle, regulation of myeloid cell differentiation, regulation of myeloid leukocyte differentiation, regulation of organ morphogenesis, regulation of protein binding, regulation of stem cell proliferation, regulation of wound healing, renal system development, repressing transcription factor binding, response to drug, response to mechanical stimulus, response to radiation, ribonucleoside triphosphate biosynthetic process, single-organism carbohydrate catabolic process, skeletal muscle cell differentiation, skeletal muscle organ development, skeletal muscle tissue development, stem cell proliferation, structure-specific DNA binding, symbiosis, encompassing mutualism through parasitism, transcription regulatory region sequence-specific DNA binding, transition metal ion homeostasis, urogenital system development, viral process, Wnt signaling pathway, zymogen activation | http://www.ncbi.nlm.nih.gov/sites/entrez?db=gene&cmd=search&term=17869 |
| Rela |  | 0.534435893763858 | activating transcription factor binding, activation of immune response, cartilage development, cellular response to hormone stimulus, cellular response to hydrogen peroxide, cellular response to insulin stimulus, cellular response to nitrogen compound, cellular response to organonitrogen compound, cellular response to oxidative stress, cellular response to peptide, cellular response to peptide hormone stimulus, cellular response to reactive oxygen species, cellular response to tumor necrosis factor, connective tissue development, cytokine biosynthetic process, cytokine metabolic process, cytokine-mediated signaling pathway, epidermis development, extrinsic apoptotic signaling pathway, glial cell differentiation, gliogenesis, hair cycle, hair cycle process, hair follicle development, hepaticobiliary system development, I-kappaB kinase/NF-kappaB signaling, immune response-activating signal transduction, innate immune response, insulin receptor signaling pathway, interleukin-12 biosynthetic process, interleukin-12 production, liver development, molting cycle, molting cycle process, negative regulation of apoptotic signaling pathway, negative regulation of catabolic process, negative regulation of extrinsic apoptotic signaling pathway, negative regulation of insulin receptor signaling pathway, negative regulation of protein catabolic process, positive regulation of cell development, positive regulation of cytokine biosynthetic process, positive regulation of defense response, positive regulation of glial cell differentiation, positive regulation of gliogenesis, positive regulation of I-kappaB kinase/NF-kappaB signaling, positive regulation of immune response, positive regulation of neurogenesis, positive regulation of NF-kappaB transcription factor activity, positive regulation of sequence-specific DNA binding transcription factor activity, protein N-terminus binding, regulation of cellular response to insulin stimulus, regulation of cytokine biosynthetic process, regulation of extrinsic apoptotic signaling pathway, regulation of glial cell differentiation, regulation of gliogenesis, regulation of I-kappaB kinase/NF-kappaB signaling, regulation of inflammatory response, regulation of insulin receptor signaling pathway, regulation of interleukin-12 biosynthetic process, regulation of interleukin-12 production, regulation of protein catabolic process, repressing transcription factor binding, response to bacterium, response to hydrogen peroxide, response to inorganic substance, response to insulin, response to interleukin-1, response to light stimulus, response to muramyl dipeptide, response to oxidative stress, response to peptide, response to peptide hormone, response to radiation, response to reactive oxygen species, response to tumor necrosis factor, response to UV, RNA polymerase II distal enhancer sequence-specific DNA binding transcription factor activity, sequence-specific DNA binding RNA polymerase II transcription factor activity, skin development, small conjugating protein ligase binding, ubiquitin protein ligase binding | http://www.ncbi.nlm.nih.gov/sites/entrez?db=gene&cmd=search&term=19697 |
| Bax |  | 0.5274989589539565 | activation of cysteine-type endopeptidase activity, activation of cysteine-type endopeptidase activity involved in apoptotic process, activation of immune response, anatomical structure homeostasis, antigen receptor-mediated signaling pathway, apoptotic mitochondrial changes, apoptotic process involved in development, apoptotic process involved in morphogenesis, appendage development, appendage morphogenesis, B cell activation, B cell apoptotic process, B cell homeostasis, blood vessel morphogenesis, blood vessel remodeling, branching morphogenesis of an epithelial tube, calcium ion homeostasis, calcium ion transport, calcium ion transport into cytosol, cellular calcium ion homeostasis, cellular divalent inorganic cation homeostasis, cellular process involved in reproduction in multicellular organism, cellular response to topologically incorrect protein, cellular response to unfolded protein, cerebral cortex development, cytosolic calcium ion homeostasis, cytosolic calcium ion transport, development of secondary sexual characteristics, developmental programmed cell death, embryonic appendage morphogenesis, embryonic digit morphogenesis, embryonic limb morphogenesis, endoplasmic reticulum calcium ion homeostasis, endoplasmic reticulum unfolded protein response, epithelial cell proliferation, epithelial tube morphogenesis, ER-nucleus signaling pathway, establishment of protein localization to membrane, establishment of protein localization to mitochondrion, establishment of protein localization to organelle, execution phase of apoptosis, extrinsic apoptotic signaling pathway, extrinsic apoptotic signaling pathway in absence of ligand, extrinsic apoptotic signaling pathway via death domain receptors, fibroblast proliferation, forebrain development, heat shock protein binding, homeostasis of number of cells, homeostasis of number of cells within a tissue, immune response-activating signal transduction, inner mitochondrial membrane organization, intrinsic apoptotic signaling pathway, intrinsic apoptotic signaling pathway by p53 class mediator, intrinsic apoptotic signaling pathway in response to DNA damage, kidney development, leukocyte apoptotic process, leukocyte homeostasis, leukocyte proliferation, limb development, limb morphogenesis, lymphocyte apoptotic process, lymphocyte differentiation, lymphocyte homeostasis, lymphocyte proliferation, male gamete generation, mammary gland epithelial cell proliferation, mitochondrial fission, mitochondrial fragmentation involved in apoptotic process, mitochondrial fusion, mitochondrial membrane organization, mitochondrial outer membrane, mitochondrial outer membrane permeabilization, mitochondrial outer membrane permeabilization involved in programmed cell death, mitochondrial transport, mitochondrion organization, mononuclear cell proliferation, morphogenesis of a branching epithelium, morphogenesis of a branching structure, multicellular organismal homeostasis, myeloid cell homeostasis, necrotic cell death, negative regulation of apoptotic signaling pathway, negative regulation of binding, negative regulation of fibroblast proliferation, negative regulation of neuron apoptotic process, negative regulation of neuron death, negative regulation of peptidyl-serine phosphorylation, negative regulation of phosphorylation, negative regulation of protein binding, negative regulation of protein phosphorylation, negative regulation of sequestering of calcium ion, neuron apoptotic process, neuron death, neuron migration, odontogenesis, odontogenesis of dentin-containing tooth, organelle outer membrane, outer membrane, pallium development, peptidyl-serine modification, peptidyl-serine phosphorylation, pigmentation, positive regulation of apoptotic signaling pathway, positive regulation of catabolic process, positive regulation of cellular catabolic process, positive regulation of cysteine-type endopeptidase activity, positive regulation of cysteine-type endopeptidase activity involved in apoptotic process, positive regulation of cytosolic calcium ion concentration, positive regulation of DNA metabolic process, positive regulation of endopeptidase activity, positive regulation of extrinsic apoptotic signaling pathway, positive regulation of extrinsic apoptotic signaling pathway in absence of ligand, positive regulation of homeostatic process, positive regulation of immune response, positive regulation of intracellular transport, positive regulation of intrinsic apoptotic signaling pathway, positive regulation of ion transport, positive regulation of leukocyte apoptotic process, positive regulation of lymphocyte apoptotic process, positive regulation of mitochondrial membrane permeability, positive regulation of mitochondrial membrane permeability involved in apoptotic process, positive regulation of mitochondrion organization, positive regulation of neuron apoptotic process, positive regulation of neuron death, positive regulation of organelle organization, positive regulation of peptidase activity, positive regulation of protein complex assembly, positive regulation of protein oligomerization, positive regulation of release of cytochrome c from mitochondria, positive regulation of tissue remodeling, post-embryonic development, post-embryonic morphogenesis, post-embryonic organ development, programmed necrotic cell death, protein homooligomerization, protein insertion into membrane, protein localization to membrane, regulation of B cell apoptotic process, regulation of binding, regulation of calcium ion transport, regulation of calcium ion transport into cytosol, regulation of cysteine-type endopeptidase activity, regulation of cysteine-type endopeptidase activity involved in apoptotic process, regulation of developmental pigmentation, regulation of DNA metabolic process, regulation of endopeptidase activity, regulation of epithelial cell proliferation, regulation of extrinsic apoptotic signaling pathway, regulation of extrinsic apoptotic signaling pathway in absence of ligand, regulation of fibroblast proliferation, regulation of homeostatic process, regulation of intrinsic apoptotic signaling pathway, regulation of ion homeostasis, regulation of leukocyte apoptotic process, regulation of lymphocyte apoptotic process, regulation of metal ion transport, regulation of mitochondrial membrane permeability, regulation of mitochondrial membrane permeability involved in apoptotic process, regulation of mitochondrion organization, regulation of neuron apoptotic process, regulation of neuron death, regulation of peptidyl-serine phosphorylation, regulation of protein binding, regulation of protein complex assembly, regulation of protein oligomerization, regulation of release of cytochrome c from mitochondria, regulation of sequestering of calcium ion, regulation of tissue remodeling, release of cytochrome c from mitochondria, release of sequestered calcium ion into cytosol, renal system development, reproductive structure development, reproductive system development, response to axon injury, response to endoplasmic reticulum stress, response to light stimulus, response to radiation, response to topologically incorrect protein, response to unfolded protein, response to UV, sex differentiation, signal transduction by p53 class mediator, signal transduction in absence of ligand, single-organism cellular localization, single-organism localization, spermatogenesis, T cell apoptotic process, T cell homeostasis, T cell proliferation, telencephalon development, thymocyte apoptotic process, tissue homeostasis, tissue remodeling, urogenital system development, zymogen activation | http://www.ncbi.nlm.nih.gov/sites/entrez?db=gene&cmd=search&term=12028 |
| Sqstm1 |  | 0.5212919719387331 | autophagic vacuole, autophagy, I-kappaB kinase/NF-kappaB signaling, protein kinase C binding, regulation of I-kappaB kinase/NF-kappaB signaling, ribonucleoprotein granule | http://www.ncbi.nlm.nih.gov/sites/entrez?db=gene&cmd=search&term=18412 |
| Nfkb1 |  | 0.5180681223470625 | alcohol biosynthetic process, aminoglycan biosynthetic process, aminoglycan metabolic process, canonical Wnt signaling pathway, carbohydrate biosynthetic process, cellular response to biotic stimulus, cellular response to lipid, cellular response to lipopolysaccharide, cellular response to molecule of bacterial origin, cytokine biosynthetic process, cytokine metabolic process, double-stranded DNA binding, fat-soluble vitamin biosynthetic process, fat-soluble vitamin metabolic process, glycosaminoglycan biosynthetic process, glycosaminoglycan metabolic process, heat shock protein binding, hyaluronan biosynthetic process, hyaluronan metabolic process, interleukin-12 biosynthetic process, interleukin-12 production, lymph node development, negative regulation of cytokine biosynthetic process, negative regulation of cytokine production, negative regulation of monooxygenase activity, negative regulation of oxidoreductase activity, organic hydroxy compound biosynthetic process, positive regulation of canonical Wnt signaling pathway, positive regulation of carbohydrate metabolic process, positive regulation of Wnt signaling pathway, regulation of canonical Wnt signaling pathway, regulation of carbohydrate biosynthetic process, regulation of carbohydrate metabolic process, regulation of cytokine biosynthetic process, regulation of inflammatory response, regulation of interleukin-12 biosynthetic process, regulation of interleukin-12 production, regulation of lipid biosynthetic process, regulation of lipid metabolic process, regulation of monooxygenase activity, regulation of oxidoreductase activity, regulation of steroid biosynthetic process, regulation of steroid metabolic process, regulation of vitamin metabolic process, regulation of Wnt signaling pathway, response to bacterium, response to lipopolysaccharide, response to molecule of bacterial origin, response to oxidative stress, steroid biosynthetic process, structure-specific DNA binding, transcription regulatory region sequence-specific DNA binding, vitamin biosynthetic process, vitamin D biosynthetic process, vitamin D metabolic process, vitamin metabolic process, Wnt signaling pathway | http://www.ncbi.nlm.nih.gov/sites/entrez?db=gene&cmd=search&term=18033 |
